# Genome-wide expression analysis upon constitutive activation of the HacA bZIP transcription factor in *Aspergillus niger* reveals a coordinated cellular response to counteract ER stress

**DOI:** 10.1186/1471-2164-13-350

**Published:** 2012-07-30

**Authors:** Neuza DSP Carvalho, Thomas R Jørgensen, Mark Arentshorst, Benjamin M Nitsche, Cees AMJJ van den Hondel, David B Archer, Arthur FJ Ram

**Affiliations:** 1 Institute of Biology Leiden, Leiden University, Molecular Microbiology and Biotechnology, Sylviusweg 72, 2333, BE Leiden, The Netherlands; 2 Kluyver Centre for Genomics of Industrial Fermentation, P.O box 5057, 2600, GA Delft, The Netherlands; 3 School of Biology, University of Nottingham, University Park, Nottingham, NG7 2RD, United Kingdom; 4 Present address: Protein Expression, Novo Nordisk, Novo Nordisk Park, 2760, Måløv, Denmark; 5 Present address: Applied and Molecular Microbiology, Institute of Biotechnology, Berlin University of Technology, Gustav-Meyer-Allee 25, 13355, Berlin, Germany

**Keywords:** HacA, Unfolded protein response, Secretion stress, RESS, XBP1, *Aspergillus niger*, Protein secretion

## Abstract

**Background:**

HacA/Xbp1 is a conserved bZIP transcription factor in eukaryotic cells which regulates gene expression in response to various forms of secretion stress and as part of secretory cell differentiation. In the present study, we replaced the endogenous *hacA* gene of an *Aspergillus niger* strain with a gene encoding a constitutively active form of the HacA transcription factor (HacA^CA^). The impact of constitutive HacA activity during exponential growth was explored in bioreactor controlled cultures using transcriptomic analysis to identify affected genes and processes.

**Results:**

Transcription profiles for the wild-type strain (HacA^WT^) and the HacA^CA^ strain were obtained using Affymetrix GeneChip analysis of three replicate batch cultures of each strain. In addition to the well known HacA targets such as the ER resident foldases and chaperones, GO enrichment analysis revealed up-regulation of genes involved in protein glycosylation, phospholipid biosynthesis, intracellular protein transport, exocytosis and protein complex assembly in the HacA^CA^ mutant. Biological processes over-represented in the down-regulated genes include those belonging to central metabolic pathways, translation and transcription. A remarkable transcriptional response in the HacA^CA^ strain was the down-regulation of the AmyR transcription factor and its target genes.

**Conclusions:**

The results indicate that the constitutive activation of the HacA leads to a coordinated regulation of the folding and secretion capacity of the cell, but with consequences on growth and fungal physiology to reduce secretion stress.

## Background

The secretion of extracellular proteins is very important to the natural saprophytic lifestyle of *Aspergillus niger*. The inherent ability of efficient protein secretion, found among several *Aspergillus* species such as *A. niger * and *A. oryzae*, has led to their biotechnological exploitation as hosts for homologous and heterologous protein production [1-5]. As protein yields for heterologous proteins are often reported as low, efforts have been made in order to describe and understand the processes that limit their secretion [6,7], as well as efforts to prevent proteolytic activity outside the cell [4,8,9].

Secretory proteins begin their journey by entering the endoplasmic reticulum (ER) where they are assembled, folded and modified. Then, they are packed into COPII coated vesicles and transported into the Golgi-like structures where further modifications take place. Proteins destined for secretion are packed into secretory vesicles to be transported to the tip of the growing hyphae, where the proteins are released extracellularly [6,10,11]. Among the factors that disturb efficient secretion of heterologous proteins is the mis-folding of these proteins in the ER and the consequence that those proteins are recognized as mis-folded by the Quality Control system present in the ER [12,13]. The presence or accumulation of aberrant proteins in the ER may become fatal to the cell and to deal with the presence of mis-folded proteins in the ER, eukaryotic cells react with the expression of several genes related to protein folding and degradation, a response termed the Unfolded Protein Response (UPR) [14]. The basic sensing pathway to detect ER stress or an increase in folding load is highly conserved from yeast to man. In *Saccharomyces cerevisiae*, the sensor protein is Ire1p which is an ER-resident trans-membrane protein that contains a luminal domain that functions as the sensor of the folded state of the proteins, and has a site-specific endoribonuclease (RNase) domain at the cytoplasmic C-terminus [15,16]. The accumulation of unfolded proteins is sensed through a dynamic interaction between Ire1p and the chaperone Kar2p (also known as Binding Protein - BiP) [17,18] or by direct sensing by Ire1p [19]. As BiP/Kar2p is recruited to help with the folding of the ER accumulating proteins, its release from Ire1p leads to the oligomerization of Ire1p proteins. In turn, the formed Ire1p oligomer is activated by autophosphorylation and the RNase domain is responsible for the splicing of a 252 nt intron present in mRNA of the bZIP transcription factor Hac1p (HacA in filamentous fungi and XBP-1 in the mammalian system), a process well characterized in fungi [20-22] and higher eukaryotes [23-26]. Alternatively, from the known structures of the yeast and human lumenal and cytoplasmic domains of Ire1p, a model for direct binding of Ire1p to unfolded proteins has been postulated that leads to structural changes in Ire1p, oligomerization and activation of the kinase and endoribonuclease domains [16,18,27,28]. In *A. niger *, the *hacA* mRNA splicing event results in the excision of a 20 nt intron [29], releasing it from a translational block [30]. Although it has not yet been shown in the *S. cerevisiae* or mammalian homologues, in addition to the intron splicing, the *hacA* mRNA of *A. niger Aspergillus nidulans* and *Trichoderma reesei* is truncated at the 5’-end during UPR induction [31,32]. However, Mulder and Nikolaev [30] showed that in *A. niger * truncation of *hacA* is not a requirement for induction of the pathway. Once translated, HacA migrates into the nucleus where it binds to palindromic UPR elements at the promoter regions of UPR targets [32]. Transcriptome analysis under UPR inducing conditions in both fungi and mammalian cells has revealed the induced expression of subsets of genes involved in folding, secretion, phospholipid biosynthesis and protein degradation [14,33-35]. Most of the UPR studies performed have induced this pathway through the presence of harsh chemicals (DTT or tunicamycin), which by itself may impose collateral responses that might provoke ER stress, and by expressing heterologous proteins such as tPA and chymosin [35-37]. However, a recent study has illustrated that the induction of UPR-target genes may not be a stress response only induced by the presence of mis-folded proteins, but may represent a more physiologically natural mechanism required and induced under conditions where there is a demand for an increased secretion capacity [38].

Manipulation of the UPR pathway and its components, like BiP1 and PDI [39-41], has been a common approach to improve the secreted production of heterologous proteins. Valkonen et al. [42] have shown, in *S. cerevisiae*, that controlling Hac1p expression has effects on native and foreign protein production; *hac1* deletion led to a decrease of heterologous α-amylase and endoglucanase production whereas overexpression of this transcription factor resulted in an increase in the production of these proteins when compared to the respective parental strains. Similar results have been demonstrated in *A. niger * var *awamori*, where a constitutive induction of the UPR pathway enhanced the production of heterologous laccase and of bovine preprochymosin [43]. The UPR is activated to alleviate the stress caused by the accumulation of mis-folded protein in the ER lumen by improving protein folding, degrading unwanted proteins [14,37] and reducing the entry of secretory proteins into the ER, a mechanism known as REpression under Secretion Stress (RESS) [44]. Studies have shown that there is a selective down-regulation of genes coding extracellular enzymes in the presence of chemicals which inhibit protein folding [44-46].

In this study, we present a genome-wide overview of the HacA responsive genes by comparing the transcriptomic profiles of two genetically engineered *A. niger * strains expressing either the wild-type *hacA* gene or the active form of the HacA transcription factor. The comparison suggests HacA as a master regulator, coordinating several processes within the secretory pathway such as the induction of protein folding, protein glycosylation and intracellular transport. Additionally, we discovered that constitutive activation of HacA results in the down regulation of the AmyR transcription factor and the AmyR regulon, which includes the most abundantly produced extracellular glycoproteins, thereby reducing import of new proteins into the ER. The down-regulation of the AmyR regulon revealed by the genome wide expression analysis was phenotypically confirmed as the HacA^CA^ mutant displayed a strongly reduced growth phenotype on starch plates.

## Results

### Construction and analysis of a strain expressing a constitutively activated form of *hacA*

**Figure 1:**
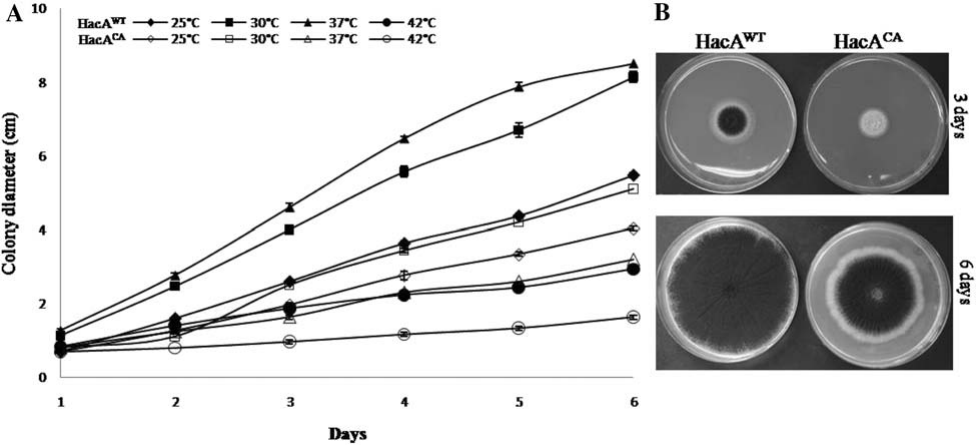
**Growth and phenotypic profiles of HacA**^**WT **^**and HacA**^**CA **^**strains.** (**A**) Differences on colony size (diameter) of HacA^WT^ and HacA^CA^ strains growing at different temperatures. 10^4^ spores were spotted on solid CM plates and growth was monitored for 6 days. (**B**) Strains phenotype on CM after 3 and 6 days of growth at 30 °C. HacA^CA^ phenotype is characterized by a slower growth/colony size as well as a delay in sporulation compared to the HacA^WT^. Bars indicate standard deviations from three individual measurements.

To obtain an *A. niger * strain with a constitutively activated HacA (HacA^CA^) transcription factor, the wild-type *hacA* gene was replaced by the spliced form of *hacA* that lacks the 20 nucleotide intron. For the construction of a reference strain and a strain only expressing the *hacA* induced form, plasmids pHacWT and pHacCA were used [Additional file 1 (A and B)]. Transformants with the correct integration pattern for each plasmid were selected after Southern blot analysis (data not shown) and the absence of the intron was confirmed in the HacA^CA^ strain [Additional file 1 (C and D)]. Growth assays were performed with both strains at different temperatures (Figure 1A and B). At each temperature tested, radial growth rate (colony size) of the HacA^CA^ strain was reduced compared to the HacA^WT^ strain, and this growth impairment was more pronounced at 37 and 42 °C (Figure 1A). Differences in phenotype between both strains were also apparent as HacA^CA^ showed a delay in growth and conidiation in comparison to HacA^WT^ (Figure 1B). As no phenotypic differences were found between our reference strain HacA^WT^ and N402 (data not shown), we conclude that the phenotypic effects observed in HacA^CA^ are due to the presence of only the UPR-induced form of *hacA*. The effects of having a constitutive activation of the UPR are different from the absence of a functional UPR. The deletion of the HacA transcription factor in *A. niger * has a profound effect on growth and morphology of the fungus, resulting in smaller and more compact colonies that hardly form conidia [30,47].

### Physiological consequences of the constitutive *hacA* activation in batch cultivations

**Figure 2:**
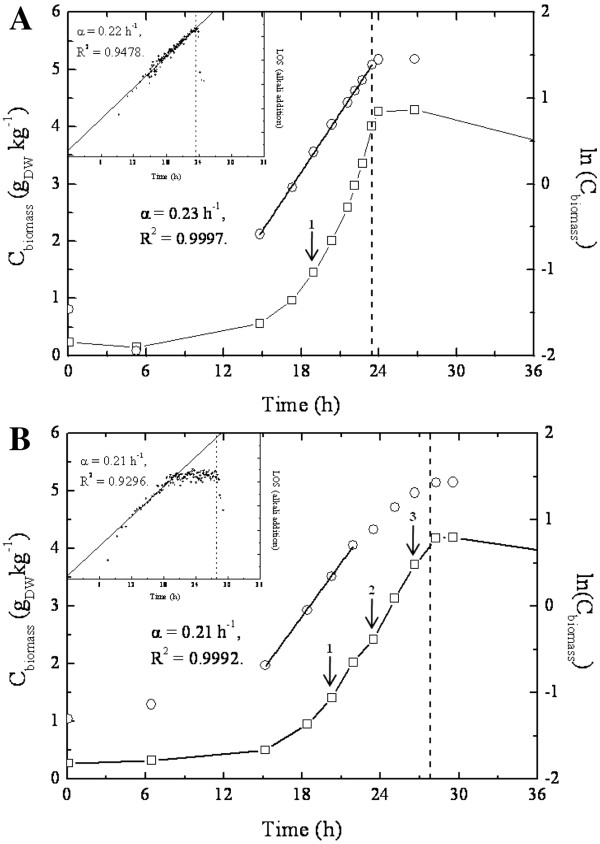
**Growth profiles of one of the triplicate *A. niger *HacA**^**WT **^**(A) and HacA **^**CA **^**(B) batch cultures.** Dry weight biomass concentration (g_DW_kg^-1^) as a function of time (h) illustrates the growth of the cultures. The maximum specific growth rate for each culture was determined from the slope (α) of the ln transformation of biomass (C_biomass_) in the exponential growth phase as a function of time (h), as well from log transformation of alkali addition as a function of time (h). Dash-line represents the end of the exponential growth phase (depletion of glucose). Arrows indicate time-points where mycelium was harvested for transcriptomic analysis.

Growth of batch cultures of the *A. niger * HacA^WT^ and HacA^CA^ strains was characterized as filamentous and highly reproducible. The growth kinetics of a representative culture of each strain is shown in Figure 2 and results from all cultures are given in the supplemental material [Additional file 2. Cultures of the HacA^WT^ strain exhibited exponential growth with a specific growth rate (μ) of 0.22 ±0.01 h-1 (n = 4) from exit of lag phase to depletion of glucose (Figure 2A). Initial growth of HacA^CA^ was similar to that of the HacA^WT^; it was exponential with a μ of 0.21 ±0.01 h-1 (n = 3). However, after 21–22 h of batch cultivation, when half of the glucose was consumed, the growth kinetics shifted from exponential to apparently linear (Figure 2B). It was not clear from the relatively few determinations of biomass concentration whether growth was truly linear in the second phase but this was strongly supported by analysis of the growth-dependent alkali addition (inset Figure 2A, B). We established a concordance between growth and alkali added to maintain constant pH in the cultures (not shown), and used this as an indirect measure of growth as described previously by Iversen et al. [48]. Linearity was then confirmed by log-transformation of alkali addition rates using the computer recorded titrant addition data and the LOS program [49]. During exponential growth, growth yield on substrate (Y_xs_) was comparable in both strains: 0.53 ± 0.02 for HacA^WT^ and 0.52 ± 0.04 for HacA^CA^.

### Impact of the constitutive activation of *hacA* on the transcriptome of *A. niger *

Three independent bioreactor cultures with the HacA^WT^ strain were performed. From each cultivation experiment, biomass was harvested from the mid-exponential growth phase (biomass concentration 1.5 gr/kg (Figure 2A)) and used for RNA extraction and subsequent microarray analysis (time point 1; HacA^WT-1^; [glucose] = 5.8 g/L). Likewise, for the HacA^CA^ strain three bioreactor cultivations were performed and biomass was harvested from each culture and RNA was isolated from the mid-exponential time point (time point 1; HacA^CA-1^; [glucose] = 5.7 g/L) (Figure 2B). Global transcription profiles were determined in triplicate for mid-exponential growth phase of HacA^WT^ strain cultures and at the corresponding biomass concentration for the HacA^CA^ strain cultures, represented by the arrows in Figure 2A and 2B. For the HacA^CA^ cultures, RNA was extracted from two additional time points subsequent to the shift to linear growth and the RNA was also analyzed (time point 2 and 3; HacA^CA-2^; [glucose] = 3.5 g/L and HacA^CA-3^; [glucose] = 1.2 g/L) (Figure 2B). Thus, the data set in this study consists of four groups of triplicate biological replicates of HacA^WT^ and HacA^CA^ at three time points (HacA^CA-1^, HacA^CA-2^ and HacA^CA-3^). The reproducibility of triplicate array analyses was high with a mean coefficient of variation (CV) ranging from 0.12 to 0.14 for transcripts rated as present or marginal.

**Figure 3:**
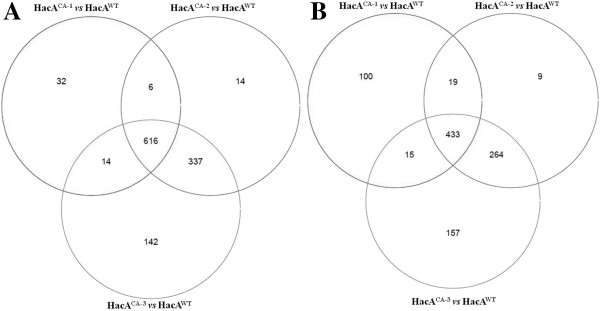
**Analysis of differentially expressed genes in HacA**^**CA **^**at all three time points in comparison to HacA**^**WT **^**.** Venn diagrams of the number of overlapping and non-overlapping induced (**A**) or repressed (**B**) genes on *A. niger * HacA^CA^ mutant strain at different time points in comparison to HacA^WT^ strain

The number of differentially-expressed genes (FDR <0.005) in a pair-wise comparison are given in Table 1. In response to constitutive activation of *hacA* at time point 1 (HacA^CA-1^), 1235 genes were differentially expressed. The number of differentially expressed genes increased when comparing the later time points (HacA^CA-2^ and HacA^CA-3^) to the wild-type strain to give a total number of 1698 and 1978 differentially expressed genes. Table 1 also shows that the transcriptomic differences between the different time points of the constitutive HacA strain were relatively minor (48 and 179 differentially expressed genes comparing HacA^CA-2^ vs. HacA^CA-1^ and HacA^CA-3^ vs. HacA^CA-1^ respectively). Comparison of HacA^CA-2^ with HacA^CA-3^ revealed very similar transcriptomes and with the stringent FDR of <0.005, no differentially expressed genes were detected. As a start to analyse the expression data, Venn diagrams were made to identify genes that were differentially expressed in HacA^CA^ at all three time points when compared to the wild-type strain. As shown in Figure 3A, 616 genes were up-regulated in the constitutive HacA strain at all three time points and 433 genes were down-regulated (Figure 3B). A complete list of all expression data and the FDR-values for the pair-wise comparison of the different strains and time points is given in [Additional file 3].

**Table 1:** Overview of the number of differentially expressed genes

	**HacA**^**WT**^		**HacA**^**CA-1**^		**HacA**^**CA-2**^	
**HacA**^**CA-1**^	1235	668 ↑				
		567 ↓				
**HacA**^**CA-2**^	1698	973 ↑	48	43 ↑		
		725 ↓		5 ↓		
**HacA**^**CA-3**^	1978	1109 ↑	179	155 ↑	0	0 ↑
		869 ↓		24 ↓		0 ↓

↑ up-regulated; ↓ down-regulated.

From the 616 up-regulated genes [Additional file 4 we were able to retrieve 598 upstream regions. These upstream regions were analysed for the presence of UPRE sequences (5’-CAN(G/A)NTGT/GCCT-3’, [32]). From the up-regulated genes in the HacA^CA^ strain, we found 47 genes that contained at least one UPRE sequence within the 400 bp region up-stream their start codon [Additional file 5. Compared to the frequency of UPRE in the 400 bp up-stream region of the remaining non up-regulated genes (457 out of 13156) a statistical significant enrichment (p ≤ 5.4 × 10^-7^) was assessed with the Fisher's exact test (one-sided). Although this analysis indicates a statistical enrichment for genes containing a HacA binding site in the promoter region of HacA induced genes, it shows that only about 10% of the HacA^CA^ induced genes contain a putative HacA binding site. It suggests that either the currently used HacA binding consensus site is too stringent and that additional sequences allow HacA to bind, or that additional transcription factors are involved in the induction in response to the constitutive activation of HacA. The data set of HacA induced genes with a putative UPRE site include genes related to protein folding (as previously described [32]), lipid metabolism, transport within the cell, glycosylation, ER quality control as well as a large set of genes that code for hypothetical and unknown function proteins [Additional file 5.

### Identification of biological processes enriched in the transcriptomic profiles of the HacA^CA^ strain

**Figure 4:**
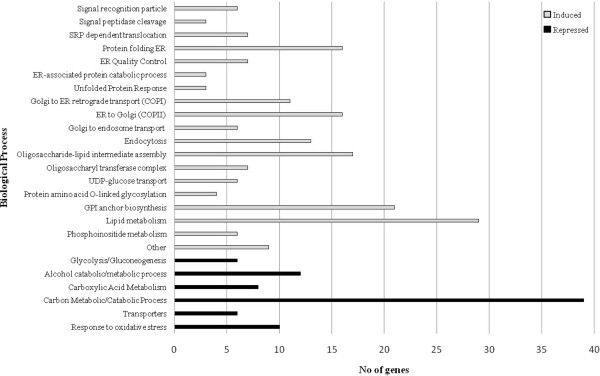
**Functional classification of differentially expressed genes in HacA**^**CA**^**.** Representation of the main significant induced and repressed biological processes in the HacA^CA^ mutant strain in comparison to HacA^WT^ strain.

To obtain an overview of the processes affected at the transcriptional level between the HacA^WT^ and the HacA^CA-1^ mutant, overrepresented GO-terms among differentially expressed genes were identified. For this analysis, we used the Fisher's exact test Gene Ontology annotation tool (FetGOat) [50]. Network maps of related GO-terms (Biological Processes), over- or under-represented in the HacA^CA^ strain, are given in Additional file 6 and Additional file 7. In Additional file 8 and Additional file 9, the results of the GO-enrichment analysis are given. To analyse the results, two complementary approaches were taken. Firstly, we rationally defined GO-terms of higher order that include several GO-terms. Secondly, we looked specifically at GO-terms that are terminal in the network, as these annotations are the most detailed (see Additional file 6). These approaches enabled us to identify four major categories of genes to describe the most relevant up-regulated biological processes in the HacA^CA^ strain (Figure 4). The four main categories of genes included those related to I) ER translocation and protein folding [Additional file 10, II) intracellular vesicle trafficking [Additional file 11, III) protein glycosylation [Additional file 12 and IV) lipid metabolism [Additional file 13. These four main categories are further described in the following section.

In the HacA^CA^ strain we found enriched GO-terms linked to ER processes, such as those related to entry in the ER: signal particle recognition, cleavage of signal sequence, and translocation (e. g. Sec61 and related subunits). In addition to the processes that mediate the recognition, targeting and entering of proteins into the ER, enriched GO-terms also included a large number of genes involved in the subsequent events of protein folding and quality control. The genes related to protein folding included the well known HacA targets such as *bipA pdiA tigA * and *prpA *[32]. After being synthesized and folded properly in the ER, proteins are packed in vesicles and transported to the Golgi and from there on, further transported to reach their final intra- or extra-cellular destination. Our analysis identified a number of genes that encode proteins that take part in the vesicle/trafficking machinery such as those involved in ER-to-Golgi (COPII associated components), Golgi-to-ER (COPI transport vesicles, Sec components) and Golgi to endosome transport. Additionally, genes involved in exocytosis were also induced (Figure 4). GO-terms related to processes involving protein glycosylation, were up-regulated in the HacA^CA^ strain. The processes include genes involved in sugar nucleotide synthesis, oligosaccharyl synthesis (ALG-genes) and transfer (OST-complex) of the preassembled oligosaccharide to certain asparagine residues (N-glycosylation). In addition, genes related to the addition of O-glycans (genes homologous to the *S. cerevisiae* Pmt-family and Kre2-family of mannosyltransferases) were up-regulated. Finally, several genes related to the synthesis and transfer of glycosylphosphatidylinositol (GPI) anchors to proteins were found to be up-regulated. Additional file 12 lists the differentially expressed genes with a proposed function in relation to protein glycosylation or GPI-anchor attachment. In addition, the constitutive activation of HacA has a pronounced effect on the transcription of genes involved in phospholipid metabolism and includes proteins that are homologous to proteins involved in ergosterol biosynthesis as well as proteins involved in the metabolism of fatty acids and inositol [Additional file 13. Categories containing fewer GO-terms included terms related to intracellular pH regulation and terms related to glutathione catabolic processes [Additional file 14.

Concerning the biological processes over-represented in the down-regulated set of genes we found one major category linked to the central metabolic pathways (Figure 4 and Additional file 15). This category includes genes within glycolysis/gluconeogenesis; alcohol catabolic/metabolic process; carboxylic acid cycle and carbon metabolic/catabolic metabolism. Categories containing fewer GO-terms included terms related to transporters and response to oxidative stress. The down-regulation of genes in central metabolic pathways may reflect the growth limitation observed in the HacA^CA^ mutant (Figures. 1 and 2).

### Common and different features of the constitutive activation of HacA and the UPR induction by chemicals or heterologous protein expression

**Figure 5:**
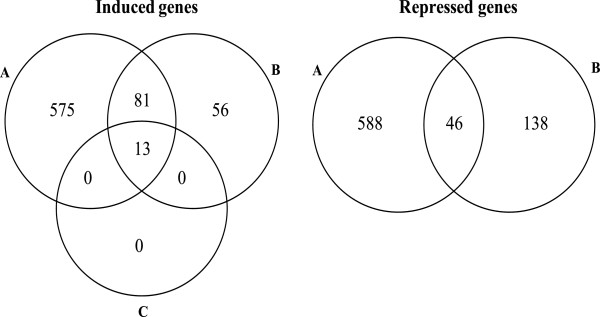
**Analysis of differentially expressed genes in HacA**^**CA **^**(this study) and Guillemette’ study[**[37]**].** Venn diagrams of the number of overlapping and non-overlapping induced or repressed genes of *A. niger * HacA^CA-1^/HacA^WT^(**A**) and genes from Guillemette et al. (2007) induced (or repressed) at least in two conditions (**B**) or induced in all conditions (**C**).

To gain a broader overview of the impact of a constitutive activation of HacA on *A. niger * we compared our data set (HacA^CA-1^/HacA^WT^) with the data of Guillemette and co-workers ([37]; [Additional file 3 and Additional file 16) in which the genome-wide transcriptional protein secretion-related stress responses was analyzed. In this study [37], transcriptional targets of the UPR pathway were identified by treatment of *A. niger * with the ER-disturbing chemical agents tunicamycin and dithiothreitol (DTT) and using a strain producing the recombinant tissue plasminogen activator (t-PA) as a model for heterologous protein production. As shown in Figure 5, in the induced set of genes, 13 genes are commonly unregulated in both studies (all conditions) and 81 genes are differentially expressed in HacA^CA-1^/HacA^WT^ in at least two of the three conditions performed by Guillemette et al. [37]. These 94 commonly induced genes include all the genes identified in the Guillemette et al.’ study related to protein folding, translocation/signal peptidase complex and glycosylation and most of the genes that belong to the categories of vesicle trafficking and lipid metabolism [Additional file 16. However, more genes belonging to each of these categories have been identified in the HacA^CA-1^/HacA^WT^ comparison (Figure 4 and Additional file 10, Additional file 11, Additional file 12, Additional file 13). Unique genes found in at least two of the conditions tested (56) and not in our data set relate mainly to the categories of cellular transport, stress related, amino acid metabolism, carbohydrate metabolism and unclassified genes.

For the repressed set of genes we found 45 common genes to our study and Guillemette et al. [37] which are evenly distributed throughout the categories established by the authors (Additional file 6 in [37]). The fact that the number of commonly down-regulated genes is small between the two studies suggests important differences and heterogeneous responses to the induction of the UPR indirectly (chemicals and heterologous protein) and the manipulation of the transcription factor that regulates this pathway in the overall cell metabolism.

### The constitutive activation of HacA triggers the induction of ERAD genes

Secretory proteins that fail to fold properly usually accumulate in the ER and are sooner or later targeted to destruction by the proteasome, a process termed ER-associated degradation (ERAD) [51]. Genes encoding proteins that are putatively involved in ERAD have been identified in the *A. niger * genome [52,53] and the expression of these genes was examined in the microarray data set. As highlighted in Table 2, the expression of several putative ERAD components was induced in the HacA^CA^ mutant. For instance, the *der1* homologue (*derA*, An01g00560), involved in transport of unfolded proteins out of the ER [54], is 4.0-fold induced; *hrd3* (*hrdC*, An03g04600), involved in recognition and presentation of the substrate for degradation [55], is 3.3-fold induced. The *mifA* (An01g14100) gene, a homologue of mammalian *herp1/mif1* protein and suggested as the link between the UPR and ERAD pathways [56], is 3.1-fold induced. Furthermore, *mns1* (*mnsA*, An18g06220), a mannosidase that by removal of 1,2 α-mannose units targets the substrate to degradation [57], is 4.2-fold induced. In comparison to Travers et al. [14], our study allowed us to unravel the regulation of other ERAD related genes in relation to UPR, such as *mns1mif1*, a DSK2 homologue An08g09000, putatively encoding a ubiquitin-like protein) (1.8-fold induction) and another putative α-mannosidase (An12g00340, 3.2-fold induced).

**Table 2:** **Expression values of *A. niger *ERAD genes**

**Gene ID**	**Gene name**	**Description**	**Fold change**		
			**HacA**^**CA-1**^**/HacA**^**WT**^	**HacA**^**CA-2**^**/HacA**^**WT**^	**HacA**^**CA-3**^**/HacA**^**WT**^
An15g00640	*derA*	strong similarity to hypothetical protein GABA-A receptor epsilon subunit – *C. elegans*	**4.0**	**6.0**	**6.4**
An01g12720	*hrdC*	similarity to tumour suppressor TSA305 protein of patent WO9928457-A1 – *H. sapiens*	**3.3**	**3.9**	**4.0**
An01g14100	*mifA*	weak similarity to stress protein Herp – *M. musculus*	**3.1**	**4.3**	**4.6**
An18g06220	*mnsA*	strong similarity to alpha-mannosidase MNS1 – *S. cerevisiae*	**4.2**	**4.7**	**5.0**
An08g09000		strong similarity to ubiquitin-like protein DSK2 – *S. cerevisiae*	**1.8**	**1.7**	**1.9**
An16g07970		similarity to autocrine motility factor receptor Amfr – *M. musculus*	**2.9**	**2.9**	**3.1**
An03g04340		strong similarity to ER membrane translocation facilitator Sec61 – *Y. lipolytica*	**2.6**	**2.6**	**2.6**
An04g01720		similarity to DnaJ protein SIS1 – *C. curvatus*	**1.8**	**2.3**	**2.2**
An12g00340		similarity to alpha 1,2-mannosidase IB – *H. sapiens*	**3.2**	**2.9**	**3.1**
An04g00360		strong similarity to transport vesicle formation protein Sec13p – *S. cerevisiae*	**2.1**	**2.1**	**2.1**
An09g06110		strong similarity to ubiquitin conjugating enzyme ubcp3p – *S. pombe*	1.4*	**1.6**	**1.7**

* Not significantly differentially expressed.

### Constitutive activation of HacA leads to the down-regulation of the AmyR regulon

Although an increase in expression of secretion related processes (folding, glycosylation, vesicle transport) is observed in the HacA^CA^ strain, the expression of several genes encoding secreted proteins is down-regulated [Additional file 15. In addition, expression of the AmyR transcription factor was repressed under these conditions (−3.3 fold, FDR < 10^-5^). Starch is a polymeric carbon source consisting of glucose units joined together by alpha1,4- and alpha1,6-glycosidic bonds and naturally synthesized by plants. *A. niger * is able to degrade starch by secreting various amylases that convert starch into maltose and glucose [58]. The transcription of these amylolytic enzymes is mediated by AmyR [59]. The AmyR regulon has been defined and consists of several alpha-glucosidases as well as two sugar transporters [58,60]. Our transcriptome profiles show that the enzymes and sugar transporters in the AmyR regulon are commonly down-regulated (Table 3).

**Table 3:** Expression values of genes involved in starch metabolism

**Gene ID**	**Gene name**	**Description**	**Fold change**		
			**HacA**^**CA-1**^**/HacA**^**WT**^	**HacA**^**CA-2**^**/HacA**^**WT**^	**HacA**^**CA-3**^**/HacA**^**WT**^
**Starch regulation**					
An04g06910	*amyR*	transcription regulator of maltose utilization AmyR – *A. niger *	**−3.3**	**−3.3**	**−3.3**
An01g06900		weak similarity to transcription activator AmyR – *A. oryzae*	−1.7*	1.4*	**2.1**
An09g03100	*amyA*	strong similarity to alpha-amylase precursor AMY – *A. shirousamii*	**−5**	**−5**	**−5**
**Starch degradation**					
An11g03340	*aamA*	acid alpha-amylase – *A. niger *	**−370**	**−50**	**−50**
An04g06920	*agdA*	extracellular alpha-glucosidase – *A. niger *	**−5**	**−10**	**−10**
An01g10930	*agdB*	extracellular alpha-glucosidase – *A. niger *	**−10**	**−10**	**−10**
An03g06550	*glaA*	glucan 1,4-alpha-glucosidase – *A. niger *	**−10**	**−25**	**−25**
An04g06930	*amyC*	extracellular alpha-amylase – *A. niger *	**−10**	**−25**	**−25**
**Sugar uptake**					
An02g03540	*mstC*	strong similarity to hexose transport protein HXT3 – *S. cerevisiae*	**−2**	**−2**	**−2**
An15g03940		strong similarity to monosaccharide transporter Mst-1 – *A. muscaria*	**−2.5**	**−2**	**−1.7**
An09g04810		strong similarity to high affinity glucose transporter HGT1 - *K. lactis*	**−5**	**−10**	**−10**
An11g01100		strong similarity to high-affinity glucose transporter HGT1 - *K. lactis*	**−5**	**−5**	**−5**
An12g07450	*mstA*	Sugar/H + symporter	**−5**	**−10**	**−10**

*Not significantly differentially expressed.

**Figure 6:**
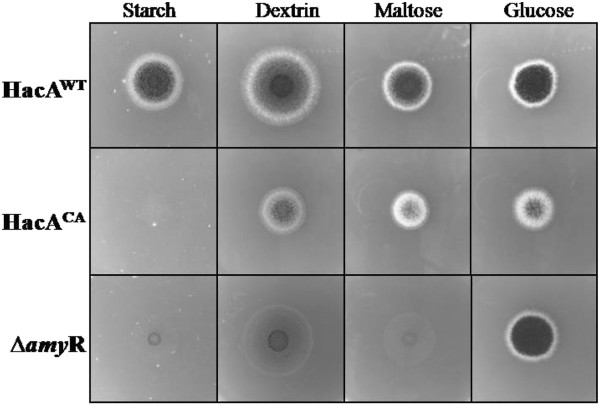
**Effects of the constitutive activation of the UPR on the utilization of starch and starch related carbon sources.** The wild-type strain (HacA^WT^), the strain containing a constitutive active form of *hacA* (HacA^CA^) and the AmyR disruptant (Δ*amyR*) strain were grown on MM containing 1% of the different carbon sources indicated at 30 °C for 3 days.

The down-regulation of genes involved in starch degradation and uptake suggested that the HacA^CA^ mutant’s growth may be severely affected on starch as sole carbon source. In order to test this, we performed growth tests of HacA^CA^ together with HacA^WT^ and a *ΔamyR* strain in which the AmyR-encoding gene has been deleted [58] on solid media containing starch or its derivatives in a range of different complexity (Figure 6).

**Figure 7:**
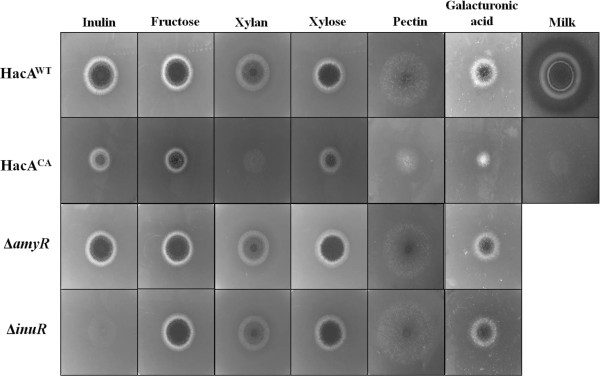
**Effects of the constitutive activation of the UPR on the utilization of different polimeric and monomeric carbon sources.** The wild-type strain (HacA^WT^), the strain containing a constitutive active form of *hacA* (HacA^CA^) the *amyR* disruptant (Δ*amyR*) and *inuR* disruptant (Δ*inuR*) strains were grown on MM containing 1% of the different carbon sources or 1% dried skim milk at 30 °C for 4 days.

As predicted from the transcriptomic data and similar to the *ΔamyR* strain, HacA^CA^ was unable to grow on the plate containing starch as sole carbon source. With the aim of testing if this reduced growth was specific for growth on starch or if it would apply to other complex carbohydrates, we performed a similar test on other polymers, inulin, xylan and pectin and respective monomeric substrates, fructose, xylose and galacturonic acid (Figure 7). In addition, growth of the HacA^CA^ strain was analysed on milk-plates (Figure 7). These results show that the HacA^CA^ strain is growth impaired when challenged to assimilate nutrients from complex substrates. Although this was not so evident when grown on inulin, growth of the HacA^CA^ strain was clearly further reduced on xylan, pectin and milk-plates suggesting that the down-regulation of extracellular enzyme expression is not limited to the amylolytic genes, but also for xylanolytic, pectinolytic and proteolytic genes.

## Discussion

### Genome-wide gene expression variations upon constitutive activation of HacA

Using a defined *A. niger * strain bearing a constitutively active form of HacA (HacA^CA^), the key regulator of the UPR pathway in eukaryotic cells, together with Affymetrix GeneChips technology, we have defined a large set of HacA-responsive genes. Unlike other studies, in which the *hacA* mRNA splicing is stimulated by the presence of unfolded proteins in the ER by chemicals or by expression of heterologous proteins [29,37], we used a different approach by creating a strain lacking the 20 nt intron in the *hacA* gene. To minimize additional effects of expressing the constitutive form of *hacA*, the *hacA*^CA^ gene was targeted to its endogenous locus. This contrasts to previous studies in which the constitutive *hacA* was expressed from a highly-expressed promoter [43] or expressed from the *pyrG* locus [30]. The microarray data revealed, even under stringent criteria (False Discovery Rate at q < 0.005), a large number of differentially-expressed genes (1235 to 1978) upon HacA activation (Table 1). The transcriptomic data obtained in our study reflects the consequences of a constitutive activation of the HacA transcription factor that results in the induction of many genes associated with the secretory pathway (Figure 4) and related to ER translocation, glycosylation, folding, quality control, ERAD, GPI anchor biosynthesis, vesicle-mediated transport between organelles (ER-Golgi), lipid metabolism, endocytosis and vacuolar sorting. Because of the highly defined conditions (both the defined mutants and the bioreactor controlled cultivations), this study revealed new categories of differentially-expressed genes as well as a much larger number of genes related to each category. Our data are however consistent with previous UPR-related studies in fungal and mammalian cells where many secretory functions are up-regulated by Hac proteins, either directly or indirectly [14,33-35,37].

Our results from the transcriptomic study also revealed that constitutive activation had a negative effect on central metabolism as well as on the production of extracellular enzymes. Although a clear growth reduction was observed for the HacA^CA^ strain on milk-plates (Figure 7), none of the main extracellular proteases (PepA, PepB, PepD or PepF)) [8] was shown to be transcriptionally down-regulated under the bioreactor growth conditions (glucose and ammonium) (Additional file 17). Possibly, the effect of downregulation of these enzymes in the HacA^CA^ strain is only occurring during inducing conditions, which might explain the reduced growth on milk-plates. The expression level of *prtT*, which encodes the transcriptional activator of extracellular proteases [8] was significantly down-regulated in the HacA^CA^ strain (Additional file 17), but this has apparently no effect of the four target genes indicated above.

As the global mechanisms for energy generation and cell development are arrested or directed towards up-regulation of the protein secretion machinery, this might account for the unbalanced growth observed in HacA^CA^ in comparison to the HacA^WT^ (Figure 2). These results suggest an implication for heterologous protein production if the protein causes ER stress. Studies on increasing heterologous protein production by enhancing UPR targets are contradictory and vary according to the protein expressed. Although protein-specific effects are likely, most studies were not controlled for the levels of chaperones or foldases co-expressed and it has been shown that there is an optimum level of both BipA [61] and PdiA [62].

GO-enrichment analysis on the induced set of genes showed that all the well-known UPR target genes related to folding are represented in the HacA^CA^ data set, and include genes encoding the chaperone BipA, and homologues of LhS1p (An01g13220), P58PK (An11g11250) and Scj1p (An05g00880), as well as the protein disulfide isomerases PdiA, PrpA and TigA . Glycosylation also appeared as one of the enriched categories. Several aspects of protein glycosylation including the categories of oligosaccharide-lipid assembly, oligosaccharyl transferase complex, UDP-glucose transport, O-linked glycosylation and GPI anchor biosynthesis (Figure 4), were up-regulated indicating that the cell responds to ER stress by increasing the capacity to glycosylate proteins. The induction of genes associated with lipid metabolism [Additional file 13 suggests a proliferation of the ER to bear the increase of proteins that reside in this organelle, as also indicated in UPR studies of *S. cerevisiae *[14].

The elimination of unfolded proteins from the ER involves the ERAD pathway [51]. Travers et al. [14] demonstrated that up-regulation of ERAD-related genes in *S. cerevisiae* is part of the UPR. These ERAD genes include DER1 and HRD3, UBC7, the ubiquitin-related DOA4, the proteasome-related PEX4 and translocon-related SEC61 [14]. From the ERAD components defined in *A. niger *[52], 11 out of 20 genes are induced in the HacA^CA^ strain (Table 2). Furthermore, analysis of the 400 bp of the up-stream regions of *derA* (An15g00640), *sec61* (An03g04340) and An04g06990 (high similarity with a human 1,2-mannosidase) revealed that these genes contain at least one UPRE sequence [Additional file 5. These results support the connection between the two pathways, as previously suggested [14,53,63,64], although the mechanistic connection between the two pathways is unresolved. We compared our data sets with those in Guillemette et al. [37] and found broad agreement with a wide range of up-regulated genes under ER stress conditions. However, Guillemette et al. [37] showed trigger-specific responses that do not complicate our analyses with HacA^CA^. Additionally, we find several putative translation initiation factors [Additional file 4, An18g06260 (highly homologous to the mammalian eIF3), repressed in HacA^CA-1^ and putative elongation factors An11g10630, An14g01030, An16g06850, An16g05260, An01g06230, An06g01710, An02g12320, An02g12420 and An04g01940 repressed in the other time points (HacA^CA-2^ and/or HacA^CA-3^).

### New leads on the RESS mechanism

The accumulation of misfolded protein in the ER leads to a selective down-regulation of genes encoding secreted proteins in fungi and plants [44-46,64]. This phenomenon is termed REpression under Secretion Stress (RESS). In these studies, associated with the UPR activation by chemical induction is the down-regulation of transcription encoding extracellular enzymes that include cellulases and xylanases in *T. reesei *[44] and glucoamylase in *A. niger *[46] amongst other genes encoding secreted proteins [37]. The mechanism by which the down-regulation is mediated is unknown, but *glaA* promoter studies in *A. niger * indicated that a promoter region between 1 and 2 kb upstream of translational start is important and a direct mediation of RESS through the UPR was questioned [46]. RESS has been recognized as an effort from the cells to prevent the entry and overload of newly synthesized proteins into the already “full” ER [44,46,64]. In our study, the activation the UPR by introducing the constitutive active form of the HacA transcription factor lead to the down-regulation not only of glucoamylase (*glaA*), but also other genes coding for starch-degrading enzymes that include acid α-amylase (*aamA*), α-glucosidases A and B (*agdA* and *agdB*) and α-amylase C (*amyC*), and additional sugar transporters (Table 3). In addition, the expression of the transcriptional activator of starch degrading enzymes is down-regulated (3.3-fold) in the HacA^CA^ strain. It has been shown previously that the AmyR transcription factor is induced (2.6-fold) upon the shift from xylose to maltose medium, suggesting that this down-regulation is biologically relevant. The down-regulation of the AmyR regulon and sugar transporters (Table 3) had a clear phenotypic effect resulting in the inability of the HacA^CA^ strain to grow on starch (Figure 6). Growth assays on other polymeric substrates (Figure 7) suggested that the down-regulation might not to be specific for starch but is relevant to other sugar polymers including xylan (Figure 7). Several scenarios can be envisioned by which the constitutive activation of HacA could result in down-regulation of secreted enzymes. We speculate that HacA activation leads to inactivation of the transcriptional factor such as AmyR (starch regulator), and possibly XlnR (xylan regulator). The inactivation results in down-regulation of the entire regulon of the transcription factor. However, a direct effect of HacA-mediated effects on individual promoters cannot be excluded. It will be of interest for future studies to determine the molecular mechanism that results in the down-regulation of AmyR and AmyR target genes in response to *HacA* activation.

### Relation between yeast, filamentous fungi and mammalian UPR counterparts

The mammalian ER contains three types of transmembrane proteins – IRE1P, PERK and ATF6 – which sense the accumulation of unfolded proteins and are responsible to activate three different branches of the UPR pathway (reviewed in [65]). Most of the players in the IRE1P pathway are conserved in fungi [66] in which, by activation of the transcription factor Hac1p/HacA, there is an induction of expression of UPR target genes related to the folding machinery [20,29], but proteins homologous to PERK and ATF6 seem to be absent from fungal systems.

To prevent the influx of proteins into the ER in mammalian cells, a mechanism of translation attenuation is activated that is mediated by PERK. This transcription factor mediates the phosphorylation of eIF2 (eukarytotic translation initiation factor) which in turn leads to the arrest of protein translation. The eIF2 is also required for the translation of selective mRNAs such as the Activating Transcription Factor-4 (ATF4) [67]. ATF4 is involved in the regulation of UPR genes involved in ERAD, metabolism and apoptosis [68]. Gcn4p/CpcA are the ATF4 homologues of *S. cerevisiae* and filamentous fungi, respectively. Both *S. cerevisiae* and *A. niger * lack an obvious PERK homologue. Gcn2p phosphorylates eIF2 leading to a global reduction on protein synthesis and stimulation of Gcn4 translation, that has been shown to control amino acid biosynthesis [69]. Although this resembles the PERK function, Gcn2p–eIF2 phosphorylation is only attributed to amino acid starvation and not to ER stress [70,71]. In *S. cerevisiae*, the involvement of Gcn2p and Gcn4p in the UPR has been shown [72]. In our transcriptomic profiles, a *gcn2* homologue (An17g00860) is not differentially expressed, whereas *cpcA* (An01g07900) shows ≈ 2 fold higher expression in comparison with the wild-type strain. According to our results the activation of *cpcA* is likely to occur in a Gcn2p-independent way and it is tempting to speculate that in filamentous fungi a similar PERK-eIF2-ATF4 pathway may exist. ATF4 is involved in glutathione biosynthesis [73] and glutathione-S-transferases have been shown to be up-regulated under ER stress conditions [74]. According to our data, the homologue to human glutathione-S-transferase 3 (An12g03580) is 2-fold induced in HacA^CA-1^ and 2.6 fold induced at the later time points. What we also observe is that as in the case of ATF4-regulated genes, not all the genes involved in glutathione metabolism are affected under secretion stress situation [73], as for example asparagine synthase (An01g07910) or glutathione reductase (An03g03660) that are not differentially expressed. Similar results have been observed in *T. reesei*[35].

Another interesting observation is the 4-fold induction of the human homologue RNA-activated protein kinase inhibitor P58 (An11g11250). In mammals, P58 is induced via ATF6, a transcription factor also involved in the regulation of UPR chaperones and apoptosis (no homologue in fungi), and it is an important component on the regulation of PERK-eIF2-ATF4 pathway, attenuating the UPR [75]. The up-regulation of P58 has been shown in studies characterizing the UPR under different conditions [37,38]; however, the role and (putative) involvement of a fungi P58 homologue in this pathway remains to be elucidated. ATF6, that induces XBP1 (HacA homologue), also possesses the ability to enhance lipid biosynthesis and expansion of the ER [76]. The identification of these potential regulatory genes involved in mediating the HacA response in this study has given multiple new leads for further research to better understand the mechanism of how *A. niger * reacts to secretion stress.

## Conclusions

The combination of a genetic defined constitutively activated HacA transcription factor mutant and controlled bioreactor cultivation conditions have provided a solid basis for a genome-wide expression analysis to study the response of *A. niger * towards ER stress. Comparison of the transcriptome obtained form the constitutive HacA mutant to previous studies in which ER stress was induced by chemical treatments or the expression of a heterologous protein revealed a consistent up-regulation of genes associated with the secretory pathway. Because of the highly defined conditions and reduced heterogeneity in our cultures, this study revealed new categories of differentially expressed genes as well as a larger number of genes related to individual categories. We also show that constitutively activation of the HacA transcription factor has a negative effect on the expression and consequently the production of extracellular enzymes. We conclude that activation of HacA induces a dual response to cope with ER stress: increasing the folding capacity of the cell by the up-regulation of genes related to secretion processes in the ER on the one hand and reducing the import of new proteins into the ER by reducing the expression of genes encoding secreted proteins on the other hand.

## Methods

### Strains and culture conditions

*Aspergillus niger* strains used throughout study (Table 4) were cultivated in minimal medium (MM) [77] containing 1% (w/v) of glucose (or other as indicated) as a carbon source, 7 mM KCl, 11 mM KH_2_PO_4_, 70 mM NaNO_3_, 2 mM MgSO_4_, 76 nM ZnSO_4_, 178 nM H_3_BO_3_, 25 nM MnCl_2_, 18 nM FeSO_4_, 7.1 nM CoCl_2_, 6.4 nM CuSO_4_, 6.2 nM Na_2_MoO_4_, 174 nM EDTA; or in complete medium (CM) containing, in addition to MM, 0.1% (w/v) casamino acids and 0.5% (w/v) yeast extract. When required, 10 mM uridine was added. The glucose minimal medium used for bioreactor cultivations has been previously described [78]. For the protease assay, strains were cultivated in MM containing 1% (w/v) dried skim milk and 0.05% Triton X100. Plates were incubated for 4 days at 30° and protease activity was verified by the appearance of a clear halo around the colony.

**Table 4:** ***Aspergillus niger *strains used in this study**

**Strain**	**Genotype**	**Reference**
N402	*cspA1 derivative of ATCC9029*	[79]
MA70.15	*ΔkusA::amdS*^*+*^*in AB4.1 pyrG*^*-*^	[80]
NC1.1	Wild type *hacA* in MA70.15, *pyrG*^*+*^	This study
NC2.1	Constitutive active *hacA* in MA70.15, *pyrG*^*+*^	This study
YvdM1.1	Δ*amy*R *in AB4.1 pyrG*^*+*^	[58]
XY3.1	Δ*inu*R *in AB4.1 pyrG*^*+*^	[81]

### Construction of the constitutive active *hacA* strain and the *hacA* reference strain

To replace to endogenous *hacA* gene on the *hacA* locus with a constitutive activated allele of the *hacA* gene, a replacement cassette was constructed. As a control, a similar replacement cassette was made with the wild-type *hacA* gene. To construct the *hacA* reference strain, three PCR fragments consisting of the *hacA* gene including promoter and terminator regions, the *Aspergillus oryzae pyrG* selection marker and a *hacA* terminator region were cloned into pBluescript-SK. Subsequently, this plasmid was used as template to introduce the mutations that led to a constitutive active *hacA* allele by site directed mutagenesis (according to Quick Change II site directed mutagenesis protocol, Stratagene). To construct the wild-type *hacA* replacement construct the *A. niger hacA* gene (accession number: AY303684), including about 0.6 kb promoter and 0.6 kb of terminator regions, was amplified by PCR using N402 genomic DNA as template and primers NC8 and NC11 (Table 5) to which *Not*I and *Xho*I restriction sites were added, respectively. The amplified gene was cloned into pTZ57R/T (Fermentas) and sequenced. The *hacA* terminator region (≈1 kb) was amplified by PCR using N402 genomic DNA as template and primers NC1 and NC2, to which *Sal*I and *Kpn*I restriction enzymes were added, respectively. The fragment was cloned into pGEM-T easy (Promega) and sequenced. For PCR amplification, Phusion™ High-Fidelity PCR Kit (Finnzymes) was used according to manufacturer’s instructions. The *AopyrG* gene (≈2 kb) was PCR amplified using pAO4-13 [80] as template DNA and primers NC7 and pAOpyrG-GA5rev, to which *Xho*I and *Sal*I restriction sites were added, respectively. The fragment was cloned into pGEM-T easy (Promega) and sequenced. The fragments corresponding to the *hacA* terminal region and *pyrG* were digested from the plasmids using the respective restriction enzymes mentioned above and cloned in a 3-way ligation step into pBlue-SK, previously digested with *Xho*I-*Kpn*I to give pBS-pyrG-3’hac. To obtain the final construct, the *hacA* gene was digested from pTZ57R/T using *Not*I-*Xho*I and cloned into pBS-pyrG-3’hac, previously digested with the same enzymes. The final construct, named pHAC, was linearized with *Not*I and transformed into the *A. niger * MA70.15 strain. Transformants with a targeted integration of the construct at the *hacA* locus were screened by Southern blot analysis. To obtain a strain only expressing the constitutively active *hacA* gene, a construct was made lacking the 20 nucleotide intron (see introduction for details) using the site-directed mutagenesis technique. Mutagenic oligonucleotide primers NC31 and NC32 (Table 5) were designed, surrounding each side of the intron region. PCR was performed using PfuUltra HF DNA polymerase (Stratagene), the pHAC (≈10 ng) as template and conditions as follows: initial denaturation of 1 min at 95 °C, 18 cycles of 30 sec denaturation at 95 °C, annealing at 55 °C for 30 sec and elongation for 8 min and 30 sec at 68 °C. Afterwards, PCR products were digested with *Dpn*I for one hour at 37 °C, for destruction of parental methylated and hemi-methylated plasmid DNA. The mixture was directly used for *E. coli * transformation. Plasmid pConstHac was analyzed by restriction enzymes and sequencing, confirming the absence of the 20 nt intron. This construct was linearized with *Not*I and then transformed into *A. niger * MA70.15. Southern analysis of putative transformants carrying the wild-type *hacA* and the constitutively active *hacA* was performed by digesting the genomic DNA with *Nhe*I and probing with a 0.6 kb probe corresponding to the *hacA* 3’-flanking region. Transformants NC1.1 containing expressing the wild-type *hacA* and NC2.1 expressing the activated *hacA* form at the endogeneous *hacA* locus were chosen for further studies and these strains are here referred as the HacA^WT^ (wild-type) and HacA^CA^ (Constitutive Active) strains, respectively. The absence of the intron in the NC2.1 strain was further confirmed by PCR analysis using genomic DNA as template, together with primers phac1 and phac2 (Table 5) using Taq polymerase (Fermentas).

**Table 5:** Primers used throughout this study

**Name**	**Sequence**
pNC1	ACGCGTCGACGCTGTTGAGGTTCCGGCTGTA
pNC2	GGGGTACCAATCTTCAGAGCGCGCCAG
pNC7	CCGCTCGAGGGATCTCAGAACAATATACCAG
pAOpyrG-GA5rev	ACGCGTCGACCCGCTGTCGGATCAGGATTA
pNC8	ATAAGAATGCGGCCGCCTCCATACCACTTTGTGCTAG
pNC11	CCGCTCGAGGGCGCATGAGAGAGTTAGG
pNC31	CGTGACACAACATCCTCCAGCGGTGTTGTGCGACCTCCAGTGTCCGTCGCTGG
pNC32	CCAGCGACGGACACTGCAGGTCGCACAACACCGCTCCAGGATGTTGTGTCACG
phac1	CTTCTCCTACCCTAACTCCT
phac2	TCAAAGAGAGAGAGGGCA

### Bioreactor cultivation conditions

Conidia for inoculation of bioreactor cultures were harvested from solidified CM with a sterile detergent solution containing 0.05% (w/v) Tween80 and 0.9% (w/v) NaCl. Batch cultivation of HacA^WT^ and HacA^CA^ was initiated by inoculating 5 L MM with conidial suspension to give 10^9^ conidia L^-1^. Glucose was sterilized separately and added to sterile MM to give a final concentration of 0.75% (w/v). During cultivation at 30 °C, pH 3 was maintained by computer-controlled addition of 2 M NaOH or 1 M HCl. Sterile air was supplied at 1 L min^-1^ through a ring-sparger. Dissolved oxygen tension was above 40% of air saturation at any time, ensuring sufficient oxygen for growth. After spore germination 0.01% (v/v) polypropyleneglycol P2000 was added as antifoam agent. Submerged cultivation was performed with 6.6 L BioFlo3000 bioreactors (New Brunswick Scientific, NJ, USA). A more detailed description of the medium and batch cultivation protocol is given in Jørgensen et al. [78].

### Biomass concentration and substrate determination

Dry weight biomass concentration was determined by weighing lyophilized mycelium separated from a known mass of culture broth. Culture broth was filtered through GF/C glass microfibre filters (Whatman). The filtrate was collected and frozen for use in solute analyses. The mycelium was washed with demineralised water, rapidly frozen in liquid nitrogen and stored at −80 °C until lyophilization. Glucose was determined according to the method of Bergmeyer et al. [83] with a slight modification: 250 mM triethanolamine (TEA) was used as buffer (pH7.5).

### RNA isolation and quality control

Mycelium intended for gene-expression analyses was separated from culture medium and frozen in liquid nitrogen within 15–20 s from sampling RNA was extracted from mycelium and quickly frozen in liquid nitrogen using Trizol reagent (Invitrogen). Frozen ground mycelium (≈200 mg) was directly suspended in 800 μl Trizol reagent and vortexed vigorously for 1 min. After centrifugation for 5 min at 10000 × g, 450 μl of the supernatant was transferred to a new tube. Chloroform (150 μl) was added and after 3 min incubation at room temperature, samples were centrifuged and the upper aqueous phase was transferred to a new tube to which 400 μl of isopropanol was added, followed by 10 min incubation at room temperature and centrifugation for 10 min at 10000 × g. The pellet was washed with 75% (v/v) ethanol and finally dissolved in 100 μl H_2_O. RNA samples for micro-array analysis were additionally purified on NucleoSpin RNA II columns (Machery-Nagel) according to the manufacturer’s instructions. RNA quantity and quality was determined on a Nanodrop spectrophotometer.

### Microarray analysis

Probe synthesis and fragmentation were performed at ServiceXS (Leiden, The Netherlands) according to the GeneChip Expression Analysis Technical Manual [82]. DSM (Delft, The Netherlands) proprietary *A. niger * GeneChips were hybridised, washed, stained and scanned as described in the GeneChip Expression Analysis Technical Manual [84]. The 3’ to 5’ signal ratio of probe sets of internal control genes, like *gpdA* (glyceraldehyde-3-phosphate dehydrogenase), *pkiA* (pyruvate kinase), *hxk* (hexokinase) and actin, were below 3 on all 12 arrays.

### Transcriptomic data analysis

Bioconductor, a collection of open source and open development packages for the statistical programming language R, was used for data analyses [85,86]. The transcriptomic data set comprises 12 arrays representing independent triplicates for each of the following four conditions: HacA^WT^, HacA^CA-1^, HacA^CA-2^ and HacA^CA-3^. Using the robust multi-array analysis (RMA) package [87], RMA expression values were computed from the perfect match probes only. Background correction, normalization and probe summarization steps were performed according to the default settings of the RMA package. Defining the following contrast matrix (HacA^CA-1^ - HacA^WT^, HacA^CA-2^ - HacA^WT^, HacA^CA-3^ - HacA^WT^), three sets of differentially expressed genes were determined by moderated t-statistics using the Limma package [88]. The Benjamini and Hochberg False Discovery Rate [89] (FDR) was controlled at q < 0.005. A minimal fold change criterion was not applied for the identification of differentially expressed genes, as fold changes are not necessarily related to biological relevance [90,91]. RMA expression values (log2 scale) for each array, mean expression values (normal scale) for each condition, fold-changes and FDR q-values for each of the three comparisons as well as classifiers for the moderated t-statistics are summarized in [Additional file 3. Results are presented as the relative fold change in a linear scale. To make the interpretation more intuitive, we have expressed the relative reduction in transcript level (down-regulated genes) with a “-” (minus). Microarray data described in this study is available at the GEO database under accession number GSE39070.

### Enrichment analysis of Gene Ontology (GO) terms

Controlling the FDR at q < 0.05, over-represented GO terms in sets of differentially expressed genes were determined with the Fisher's exact test Gene Ontology Annotation tool (FetGOat) [50].

## Competing interests

The authors declare that they have no competing interests.

## Authors’ contributions

NDSPC constructed the *Aspergillus* strains used in this study. NDSPC, TJR and MA carried out and analysed the bioreactor cultivations. BMN carried out the statistical analysis of the transcriptomic data. NDSPC, TRJ, BMN, DBA and AFJR interpreted the transcriptomic data. NDSPC, TRJ, MA, CAMJJvdH, and AFJR designed the experiments. All authors contributed to the writing of the manuscript. All authors read and approved the final manuscript.

## Supplementary Material

Additional file 1 **Construction plasmids and confirmation of a reference strain and a strain only expressing the *hacA *induced form.** Schematic representation of the plasmids pHAC (A) and pConstHac (B) (Note: fragment sizes are not on scale). (C) Sequence alignment of pHAC and pConstHAC showing the absence of the 20 nt intron on pConstHac. (D) PCR amplification of gDNA of HacA^WT^ (NC1.1) and HacA^CA^ (NC2.1) transformants. Primers were designed about 100 bp upstream and 100 bp downstream of the hacA intron region, giving rise to a band of 200 bp for HacA^CA^ and 220 bp for HacA^WT^. Sizes of the DNA Marker (M) are indicated. Click here for file

Additional file 2 **Growth profiles of*A. niger *HacA**^**WT **^**(A, B, C) and HacA**^**CA **^**(D, E, F) triplicate batch cultures.** Dry weight biomass concentration (g_DW_kg^-1^) as a function of time (h) illustrates the growth of the cultures. The maximum specific growth rate for each culture was determined from the slope (α) of the ln transformation of biomass (C_biomass_) (lnX) in the exponential growth phase as a function of time (h), as well from log transformation of alkali addition as a function of time (h). Dash-line represents the end of the exponential growth phase (depletion of glucose). Click here for file

Additional file 3 **Complete list of all differentially expressed genes in HacA**^**CA**^**.** Expression data and the FDR-values for the pair wise comparison of the different strains and time points. Click here for file

Additional file 4 **Overview of the 616 HacA**^**CA **^**up-regulated genes in the 3 time points.** Subset of all differentially expressed genes (Additional file 3). Click here for file

Additional file 5 **HacA**^**CA **^**up-regulated genes that contain at least one UPRE sequence.** Subset of all differentially expressed genes (Additional file 3). Click here for file

Additional file 6 **Network maps of related up-regulated GO-terms.** Results of the GO-enrichment analysis of biological processes of all differentially expressed genes in HacA^CA-1^/HacA^WT^. Click here for file

Additional file 7 **Network maps of related down-regulated GO-terms.** Results of the GO-enrichment analysis of biological processes of all differentially expressed genes in HacA^CA-1^/HacA^WT^. Click here for file

Additional file 8 **GO analysis of biological processes enriched in the up-regulated set of genes in HacA**^**CA**^**.** Subset of all differentially expressed genes (Additional file 3). Click here for file

Additional file 9 **GO analysis of biological processes enriched in the down-regulated set of genes in HacA**^**CA**^**.** Subset of all differentially expressed genes (Additional file 3). Click here for file

Additional file 10 **Expression values of selected genes related to enriched GO terms of ER associated processes.** Subset of all differentially expressed genes (Additional file 3). Click here for file

Additional file 11 **Expression values of selected genes related to enriched GO terms associated with vesicle transport within the cell.** Subset of all differentially expressed genes (Additional file 3). Click here for file

Additional file 12 **Expression values of selected genes related to enriched GO terms associated with glycosylation processes.** Subset of all differentially expressed genes (Additional file 3) Click here for file

Additional file 13 **Expression values of selected genes related to enriched GO terms associated with lipid metabolic processes.** Subset of all differentially expressed genes (Additional file 3). Click here for file

Additional file 14 **Expression values of selected genes related to the GO terms “hydrolase activity”, “glutathione catabolic processes” and “vacuolar acidification”.** Subset of all differentially expressed genes (Additional file 3). Click here for file

Additional file 15 **Expression values of selected down-regulated genes related to enriched GO terms.** Subset of all differentially expressed genes (Additional file 3). Click here for file

Additional file 16 **Commonly induced and repressed genes in the HacA**^**CA **^**strain and *A. niger *strains treated with DTT and Tunicamycin and expressing tPA.** Subset of all differentially expressed genes (Additional file 3) and Guillemette’ study [37]. Click here for file

Additional file 17 Expression values of genes related to extracellular proteases production. Click here for file
