# Supplementary figures and images for: Genome-wide expression analysis upon constitutive activation of the HacA bZIP transcription factor in Aspergillus niger reveals a coordinated cellular response to counteract ER stress
Source: BMC Genomics. 2012 Jul 30;13:350. doi: 10.1186/1471-2164-13-350 (PMC3472299; doi:10.1186/1471-2164-13-350)

## Slide 1
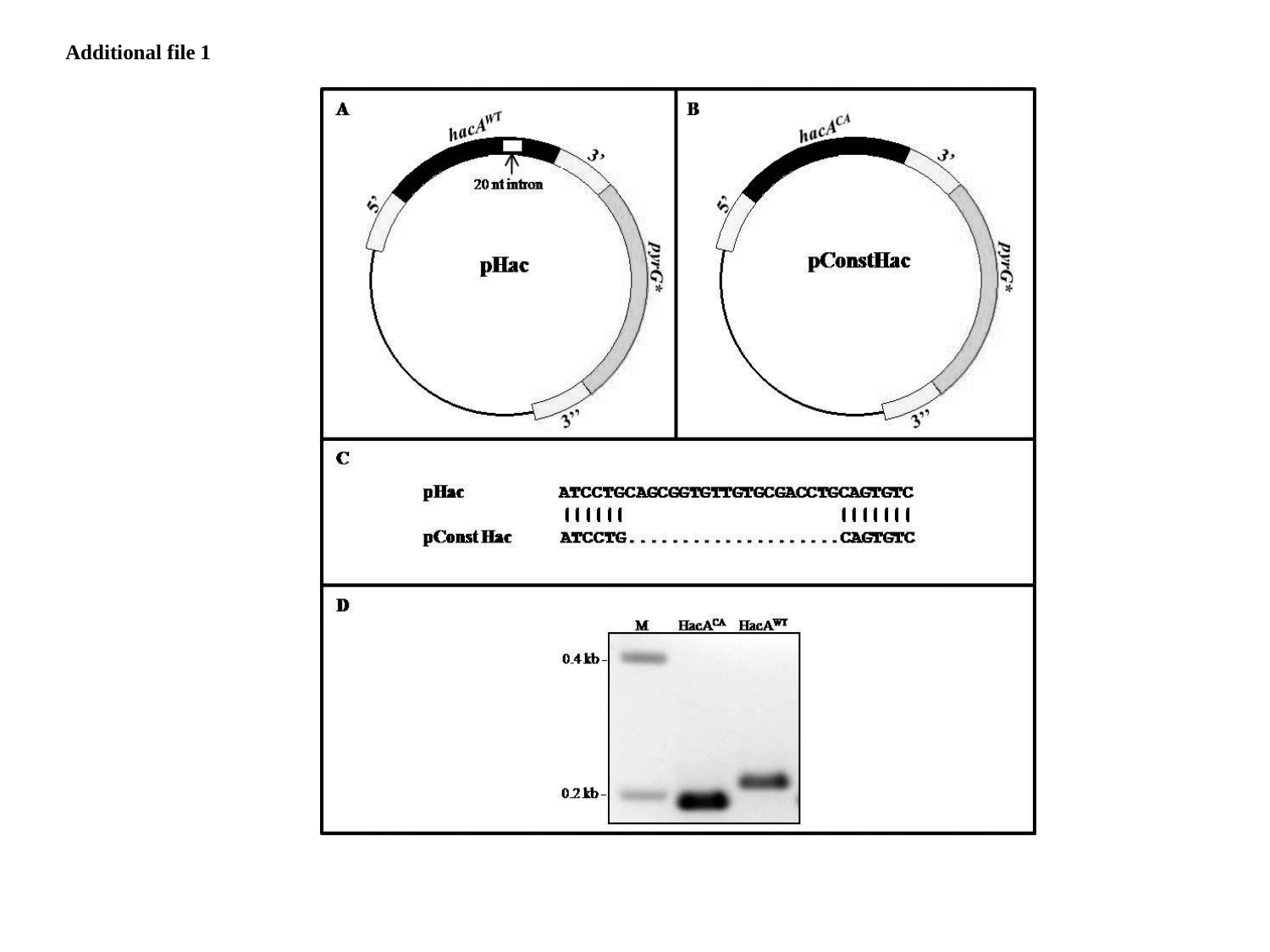

Additional file 1

Supplement: Additional file 1 — Construction plasmids and confirmation of a reference strain and a strain only expressing the hacA induced form. Schematic representation of the plasmids pHAC (A) and pConstHac (B) (Note: fragment sizes are not on scale). (C) Sequence alignment of pHAC and pConstHAC showing the absence of the 20 nt intron on pConstHac. (D) PCR amplification of gDNA of HacAWT (NC1.1) and HacACA (NC2.1) transformants. Primers were designed about 100 bp upstream and 100 bp downstream of the hacA intron region, giving rise to a band of 200 bp for HacACA and 220 bp for HacAWT. Sizes of the DNA Marker (M) are indicated. [file 1471-2164-13-350-S1.ppt]

## Slide 1
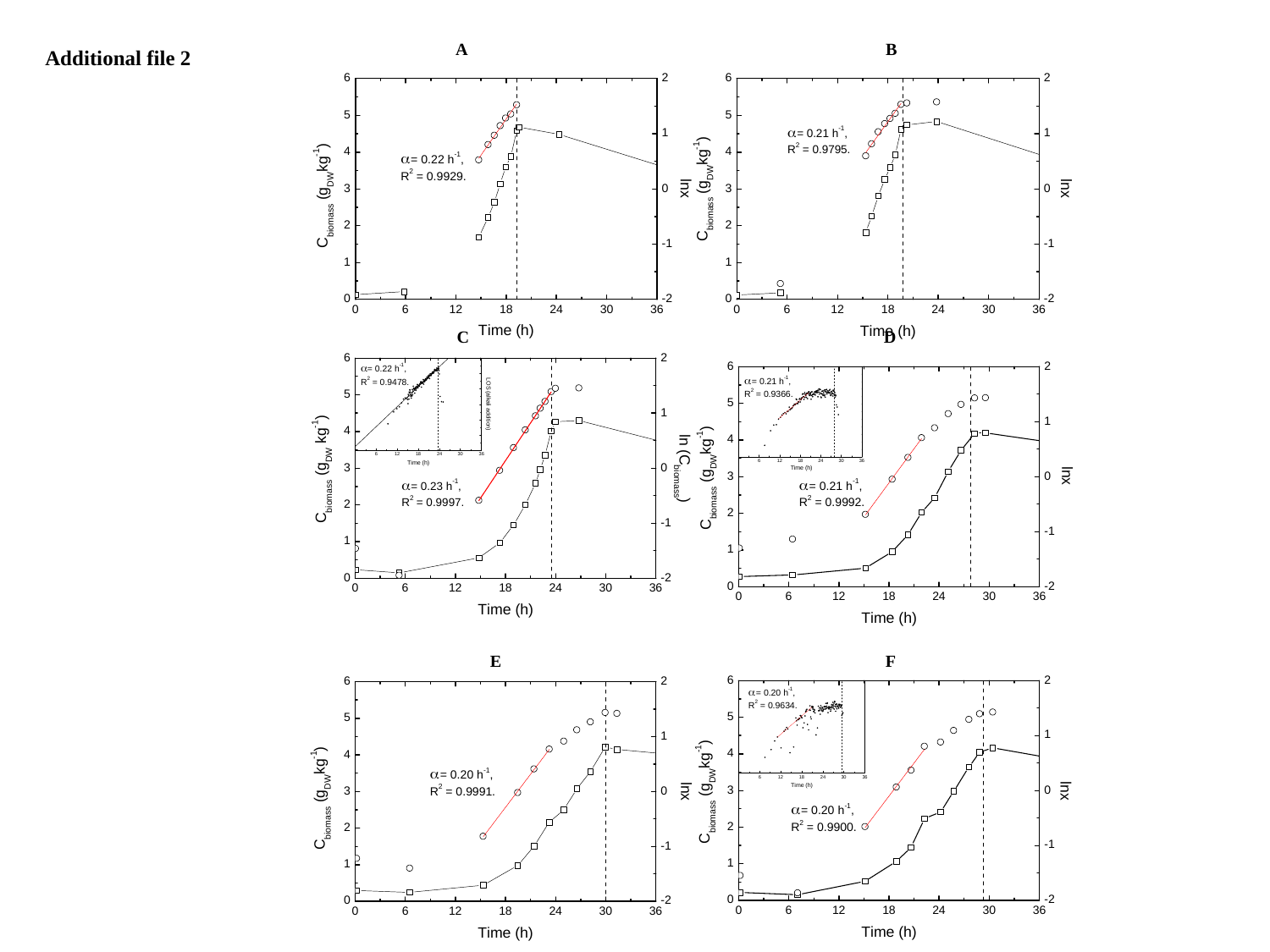

Additional file 2

Supplement: Additional file 2 — Growth profiles ofA. niger HacAWT (A, B, C) and HacACA (D, E, F) triplicate batch cultures. Dry weight biomass concentration (gDWkg-1) as a function of time (h) illustrates the growth of the cultures. The maximum specific growth rate for each culture was determined from the slope (α) of the ln transformation of biomass (Cbiomass) (lnX) in the exponential growth phase as a function of time (h), as well from log transformation of alkali addition as a function of time (h). Dash-line represents the end of the exponential growth phase (depletion of glucose). [file 1471-2164-13-350-S2.ppt]

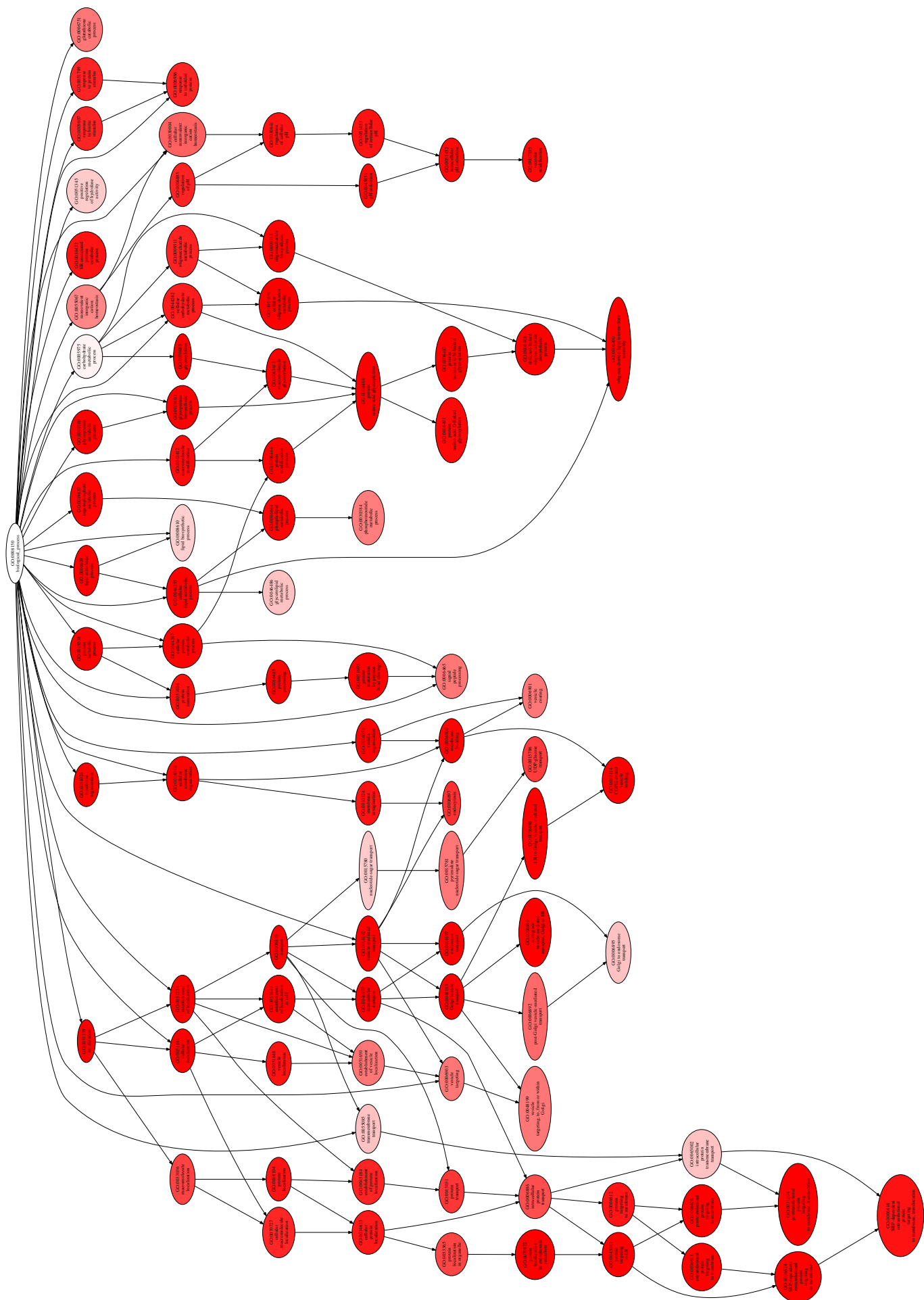

Supplement: Additional file 6 — Network maps of related up-regulated GO-terms. Results of the GO-enrichment analysis of biological processes of all differentially expressed genes in HacACA-1/HacAWT. [file 1471-2164-13-350-S6.pdf]

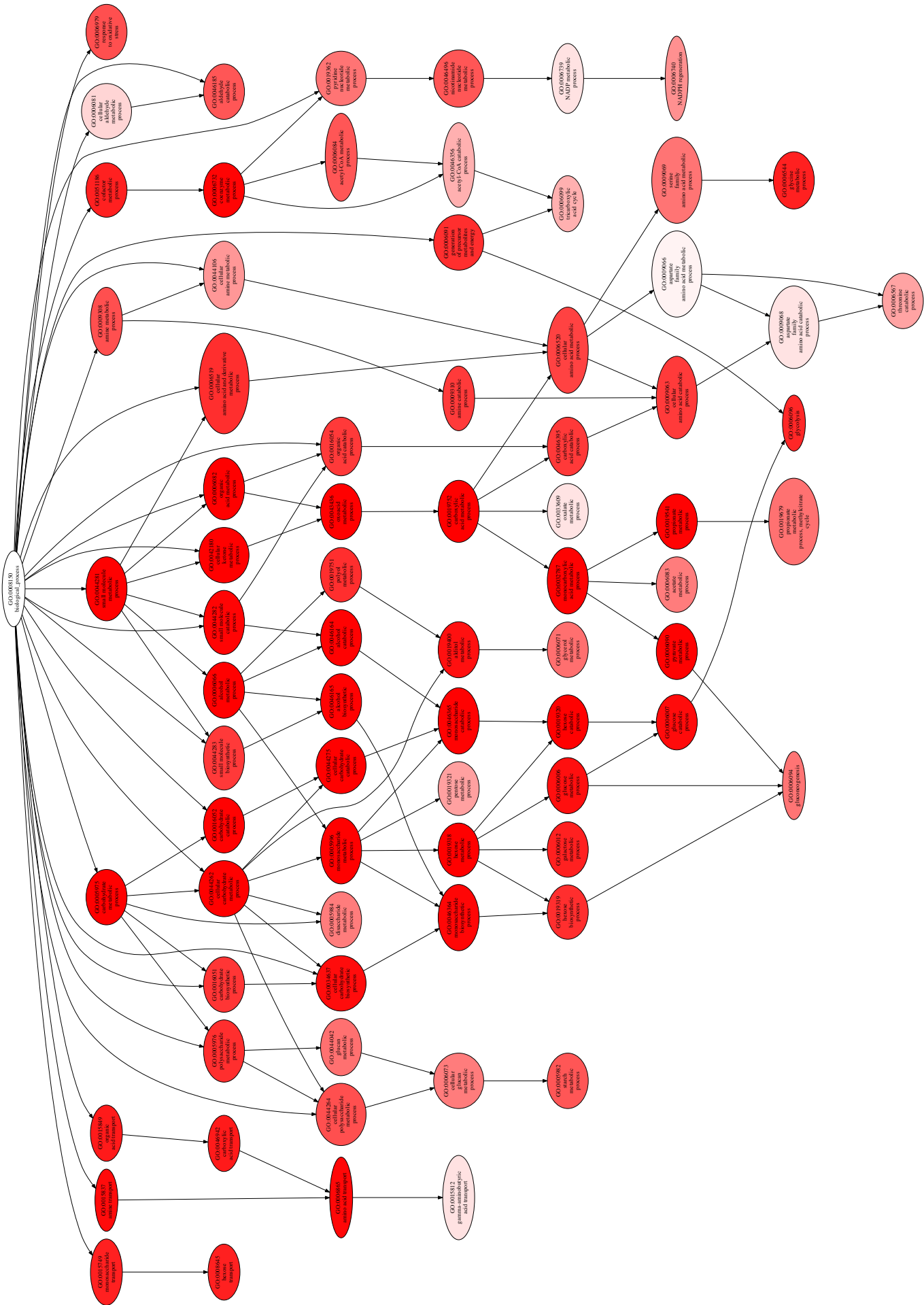

Supplement: Additional file 7 — Network maps of related down-regulated GO-terms. Results of the GO-enrichment analysis of biological processes of all differentially expressed genes in HacACA-1/HacAWT. [file 1471-2164-13-350-S7.pdf]
