# Supplementary material for: Genome-wide expression analysis upon constitutive activation of the HacA bZIP transcription factor in Aspergillus niger reveals a coordinated cellular response to counteract ER stress
Source: BMC Genomics. 2012 Jul 30;13:350. doi: 10.1186/1471-2164-13-350 (PMC3472299; doi:10.1186/1471-2164-13-350)
Supplement: Additional file 4 — Overview of the 616 HacACA up-regulated genes in the 3 time points. Subset of all differentially expressed genes (Additional file 3). [file 1471-2164-13-350-S4.doc]

Additional file 4: Overview of the 616 HacA^CA^ up-regulated genes in the 3 time points.

| **Gene ID** | **Description** |
| --- | --- |
| An01g00290 | similarity to hypothetical protein encoded by An17g00430 - *Aspergillus niger* |
| An01g00560 | strong similarity to signal peptidase subunit Sec11 - *Saccharomyces cerevisiae* |
| An01g01300 | hypothetical protein |
| An01g02280 | hypothetical protein |
| An01g02640 | strong similarity to O-methyltransferase B omtB - *Aspergillus parasiticus* |
| An01g02660 | strong similarity to hypothetical protein encoded by An03g05810 - *Aspergillus niger* |
| An01g02870 | similarity to hypothetical protein SPAC227.06 - *Schizosaccharomyces pombe* |
| An01g02910 | strong similarity to hypothetical ARE1-like protein F3I17.5 - *Arabidopsis thaliana* |
| An01g03100 | strong similarity to manganese resistance protein 1 Mnr1 - *Saccharomyces cerevisiae* |
| An01g04040 | secretion-associated GTP-binding protein sarA - *Aspergillus niger* |
| An01g04320 | strong similarity to COPII vesicle coat component protein Erv46 - *Saccharomyces cerevisiae* |
| An01g04600 | PDI related protein A prpA - *Aspergillus niger* |
| An01g04760 | similarity to hypothetical protein B12K8.10 - *Neurospora crassa* |
| An01g05200 | strong similarity to DPM2 - *Mus musculus* |
| An01g05540 | similarity to hypothetical protein T27E13.9 - *Arabidopsis thaliana* |
| An01g05670 | strong similarity to H+-transporting ATPase Pma1 - *Saccharomyces cerevisiae* |
| An01g06550 | strong similarity to protein kinase Ire1 - *Saccharomyces cerevisiae* |
| An01g06670 | strong similarity to peptidyl-prolyl isomerase FKBP-21 - *Neurospora crassa* |
| An01g06800 | strong similarity to alkaline ceramidase Ypc1 - *Saccharomyces cerevisiae* |
| An01g07320 | strong similarity to embryogenesis protein H beta 58 - *Mus musculus* |
| An01g07330 | similarity to brefeldin A-inhibited guanine nucleotide-exchange protein 2 - *Homo sapiens* |
| An01g07640 | similarity to hypothetical membrane protein YPL087w - *Saccharomyces cerevisiae* |
| An01g07900 | leucine zipper cpcA - *Aspergillus niger* [putative frameshift] |
| An01g07930 | strong similarity to hypothetical protein encoded by An11g05030 - *Aspergillus niger* |
| An01g08170 | strong similarity to DNA repair endonuclease rad1p - *Schizosaccharomyces pombe* |
| An01g08300 | similarity to hypothetical conserved protein VC1938 - *Vibrio cholerae* |
| An01g08420 | strong similarity to calcium-binding protein precursor cnx1p - *Schizosaccharomyces pombe* |
| An01g08460 | strong similarity to the mannosyltransferase Alg12 - *Saccharomyces cerevisiae* |
| An01g08530 | kexin precursor kexB - *Aspergillus niger* |
| An01g08550 | strong similarity to aminotriazole resistance protein Atr1 - *Saccharomyces cerevisiae* |
| An01g08620 | strong similarity to hypothetical protein SPBC2A9.05c - *Schizosaccharomyces pombe* |
| An01g08660 | similarity to ankyrin Ank - *Drosophila melanogaster* [truncated ORF] |
| An01g08670 | similarity to nucleolin C23 - *Cricetulus griseus* |
| An01g08870 | strong similarity to component of COPII-coated vesicles Erv25 - *Saccharomyces cerevisiae* |
| An01g09260 | strong similarity to aldehyde dehydrogenase (NADP+) - *Homo sapiens* |
| An01g09480 | strong similarity to phosphatidylserine synthase pS232 - *Triticum aestivum* |
| An01g09800 | strong similarity to hypothetical protein encoded by ORF G4P06 - *Aspergillus nidulans* |
| An01g10070 | strong similarity to signal recognition particle chain Sec65 - *Saccharomyces cerevisiae* |
| An01g10730 | similarity to plasma membrane bound receptor from patent DE19627237-A1 *- Sus scrofa* |
| An01g10900 | similarity to hypothetical la costa protein lcs - *Drosophila melanogaster* |
| An01g10970 | strong similarity to quinate transporter qa-y - *Neurospora crassa* |
| An01g11140 | similarity to acetyl transferase of an indole-3-acetic acid producing gene cluster ORF5 - *Azospirillum brasilense* |
| An01g11420 | hypothetical protein |
| An01g11630 | strong similarity to translocation complex component Sss1 - *Saccharomyces cerevisiae* |
| An01g12290 | similarity to CCAAT displacement protein CASP - *Homo sapiens* |
| An01g12370 | similarity to hypothetical protein SPCC285.11 - *Schizosaccharomyces pombe* |
| An01g12430 | strong similarity to hypothetical protein CAF32157.1 - *Neurospora crassa* |
| An01g12520 | strong similarity to beta-ketoacyl reductase rhlG - *Pseudomonas aeruginosa* |
| An01g12720 | similarity to tumour suppressor protein TSA305 from patent WO9928457-A1 - *Homo sapiens* |
| An01g12810 | similarity to BAX-associated protein fragment SEQ ID NO:242 from patent WO200264766-A2 - *Saccharomyces cerevisiae* |
| An01g12990 | strong similarity to hypothetical membrane protein YJL091c - *Saccharomyces cerevisiae* |
| An01g13070 | strong similarity to signal recognition particle receptor Sec63 - *Saccharomyces cerevisiae* |
| An01g13220 | strong similarity to 150 kDa oxygen regulated protein ORP150 - *Rattus norvegicus* |
| An01g13390 | similarity to hypothetical protein CAD21062.1 - *Neurospora crassa* |
| An01g13640 | similarity to nitrate assimilation regulatory protein nirA - *Aspergillus nidulans* |
| An01g14100 | weak similarity to stress protein Herp - *Mus musculus* |
| An01g14140 | strong similarity to CDPdiacylglycerol--inositol 3-phosphatidyltransferase - *Rattus norvegicus* |
| An01g14250 | strong similarity to delta subunit of the coatomer delta-coat protein CopD - *Bos taurus* |
| An01g14260 | strong similarity to delta subunit of the coatomer delta-coat protein CopD - *Bos taurus* [deleted ORF] |
| An01g14420 | similarity to soluble epoxide hydrolase SEH - *Homo sapiens* |
| An01g14810 | weak similarity to hypothetical protein SPAC19B12.02c - *Schizosaccharomyces pombe* |
| An01g14820 | similarity to hypothetical membrane protein YNL283c - *Saccharomyces cerevisiae* |
| An01g14840 | similarity to hypothetical protein SC4B10.33 - *Streptomyces coelicolor* |
| An02g01410 | hypothetical protein |
| An02g01510 | strong similarity to component of the endoplasmic reticulum protein translocation machinery Sec62 - *Saccharomyces cerevisiae* |
| An02g01690 | strong similarity to p150 component of the COPII coat of secretory pathway vesicles Sec31 - *Saccharomyces cerevisiae* |
| An02g02020 | strong similarity to vacuolar H(+)-transporting ATPase subunit B Vma2 - *Saccharomyces cerevisiae* |
| An02g02260 | strong similarity to hypothetical membrane protein YMR155w - *Saccharomyces cerevisiae* |
| An02g02640 | similarity to Golgi membrane protein Yip1 - *Saccharomyces cerevisiae* |
| An02g02980 | strong similarity to protein influencing Itr1 expression Die2 - *Saccharomyces cerevisiae* |
| An02g03130 | hypothetical protein |
| An02g03240 | strong similarity to UDP-N-acetylglucosamine--dolichyl-phosphate N-acetylglucosaminephosphotransferase Alg7 - *Saccharomyces cerevisiae* |
| An02g03300 | strong similarity to vacuolar ATPase subunit F - *Neurospora crassa* |
| An02g03460 | similarity to hypothetical protein YIL041w - *Saccharomyces cerevisiae* |
| An02g03600 | hypothetical protein |
| An02g04050 | similarity to protein-tyrosine phosphatase P-TEN - *Homo sapiens* |
| An02g04120 | similarity to protein SEQ ID NO:13148 from patent EP1074617-A2 - *Homo sapiens* |
| An02g04210 | strong similarity to chloride channel CLC-3 - *Tilapia mossambica* |
| An02g04250 | similarity to protein p58 - *Rattus norvegicus* |
| An02g04530 | strong similarity to cyclopropane-fatty-acyl-phospholipid synthase - *Escherichia coli* |
| An02g04580 | strong similarity to hypothetical protein YAL049c - *Saccharomyces cerevisiae* |
| An02g05120 | hypothetical protein |
| An02g05150 | strong similarity to C-8,7 sterol isomerase - *Arabidopsis thaliana* |
| An02g05180 | hypothetical protein |
| An02g05610 | strong similarity to branched-chain alpha-ketoacid dehydrogenase kinase BCKDK - *Rattus norvegicus* |
| An02g05870 | strong similarity to coatomer beta subunit copB2 - *Homo sapiens* [putative frameshift] |
| An02g05890 | strong similarity to protein disulfide-isomerase pdi1 - *Caenorhabditis elegans* |
| An02g06300 | similarity to naaladase II - *Homo sapiens* |
| An02g06870 | strong similarity to RAD50-interacting protein 1 RINT-1 - *Homo sapiens* |
| An02g07090 | strong similarity to ASNA1 product arsenite translocating ATPase - *Homo sapiens* |
| An02g07100 | hypothetical protein |
| An02g07610 | strong similarity to mannitol transporter mat1 - *Apium graveolens* |
| An02g07730 | similarity to growth regulator ING1 - *Mus musculus* [truncated ORF] |
| An02g07780 | strong similarity to ADP-ribosylation factor 6 ARF6 - *Homo sapiens* |
| An02g08060 | similarity to hypothetical protein YGL144c - *Saccharomyces cerevisiae* |
| An02g08450 | secretory gene nsfA - *Aspergillus niger* |
| An02g08840 | similarity to rabkinesin-6 - *Mus musculus* |
| An02g09180 | similarity to mucin-like protein Muc1 - *Saccharomyces cerevisiae* |
| An02g09290 | hypothetical protein |
| An02g09350 | strong similarity to sulfate transport protein shst2 - *Stylosanthes hamata* |
| An02g09470 | strong similarity to hypothetical Ca2+-transporting ATPase Spf1 - *Saccharomyces cerevisiae* [truncated ORF] |
| An02g09550 | strong similarity to protein het-c2 - *Podospora anserina* |
| An02g09910 | strong similarity to fatty acid elongase Fen1 - *Saccharomyces cerevisiae* |
| An02g10270 | strong similarity to protein involved in vacuolar protein sorting Vps54 - *Saccharomyces cerevisiae* |
| An02g10490 | strong similarity to endo-1,3(4)-beta-glucanase bg1 - *Phaffia rhodozyma* |
| An02g10560 | similarity to protein kinase/ankyrin homologue SEQ ID NO:334 from patent WO9955865-A1 - *Mus musculus* |
| An02g11290 | similarity to phosphoinositide-specific phospholipase C lc1p - *Schizosaccharomyces pombe* |
| An02g11420 | dipeptidyl aminopeptidase type IV dapB - *Aspergillus niger* |
| An02g11810 | strong similarity to hypothetical conserved protein 12F11.50 - *Neurospora crassa* |
| An02g11990 | similarity to protein involved in protein secretion sec20 - *Candida albicans* |
| An02g12050 | similarity to V-ATPase assembly protein Vma21 - *Saccharomyces cerevisiae* |
| An02g12630 | strong similarity to glucosyltransferase Alg6 - *Saccharomyces cerevisiae* |
| An02g13080 | similarity to N epsilon-(indole-3-acetyl)-L-lysine synthase iaaL - *Pseudomonas syringae* |
| An02g13410 | similarity to acetyl-coenzyme A transporter AT-1 - *Homo sapiens* |
| An02g13570 | strong similarity to protein involved in glycosylphosphatidylinositol biosynthesis pig-C - *Homo sapiens* |
| An02g13670 | similarity to human secreted protein fragment from patent WO9909155-A1 - *Homo sapiens* |
| An02g14070 | weak similarity to hypothetical protein encoded by An11g01490 - *Aspergillus niger* |
| An02g14450 | secretory pathway Ca2+-ATPase pmrA - *Aspergillus niger* |
| An02g14560 | oligosaccharyltransferase alpha subunit ostA - *Aspergillus niger* |
| An02g14800 | protein disulfide isomerase A pdiA - *Aspergillus niger* |
| An02g14930 | strong similarity to dolichyl-diphosphooligosaccharide-protein glycotransferase gamma chain Ost3 - *Saccharomyces cerevisiae* |
| An02g14940 | strong similarity to human transmembrane protein HTMPN-23 from patent WO9961471-A2 - *Homo sapiens* |
| An03g02270 | similarity to dihydropyridine receptor Fgalpha1S - *Rana catesbeiana* |
| An03g02430 | strong similarity to mannitol dehydrogenase mtlD - *Pseudomonas fluorescens* |
| An03g02530 | similarity to hypothetical proteinase encoded by SCD8A.14c - *Streptomyces coelicolor* |
| An03g02590 | hypothetical protein |
| An03g02770 | strong similarity to hypothetical protein YLR361c - *Saccharomyces cerevisiae* |
| An03g02820 | strong similarity to hypothetical lipase Cvt17 - *Saccharomyces cerevisiae* |
| An03g02990 | similarity to alphaN-acetylglucosamine transferase GNT1 - *Kluyveromyces lactis* |
| An03g03940 | similarity to potential regulator of leucine permease gene expression Sac3 - *Saccharomyces cerevisiae* |
| An03g04190 | similarity to cellulase #2 from patent US2003036176-A1 - *Xanthomonas campestris* |
| An03g04210 | similarity to component of the exocyst Sec8 - *Saccharomyces cerevisiae* |
| An03g04340 | strong similarity to ER membrane translocation facilitator Sec61 - *Yarrowia lipolytica* |
| An03g04410 | strong similarity to UDP-glucose:dolichyl-phosphate glucosyltransferase Alg5 - *Saccharomyces cerevisiae* |
| An03g04430 | strong similarity to hypothetical protein EAA61229.1 - *Aspergillus nidulans* |
| An03g04720 | strong similarity to serine threonine protein kinase SNF1 - *Cochliobolus carbonum* |
| An03g04770 | similarity to hypothetical protein CAD11383.1 - *Neurospora crassa* |
| An03g04910 | strong similarity to clathrin coat assembly protein AP19 - *Camptotheca acuminata* |
| An03g04940 | strong similarity to Erv41 - *Saccharomyces cerevisiae* |
| An03g05960 | strong similarity to epoxide hydrolase cEH - *Corynebacterium sp.* |
| An03g06330 | strong similarity to 2,3-dihydroxybiphenyl-1,2-dioxygenase bphC - *Pseudomonas stutzeri* |
| An03g06410 | strong similarity to methyl sterol oxidase Erg25 - *Saccharomyces cerevisiae* |
| An03g06420 | similarity to hypothetical protein encoded by An08g08870 - *Aspergillus niger* |
| An03g06540 | strong similarity to arylsulfatase ars-1 - *Neurospora crassa* |
| An03g06760 | strong similarity to hypothetical transcription regulator protein CAA91958.1 - *Schizosaccharomyces pombe* |
| An03g06840 | hypothetical protein |
| An03g06880 | strong similarity to carboxypeptidase Y-sorting protein Pep1 - *Saccharomyces cerevisiae* |
| An03g06900 | strong similarity to exocytosis protein Sec10 - *Saccharomyces cerevisiae* |
| An03g06910 | strong similarity to hypothetical 32.0k protein - *Neurospora crassa* |
| An03g06940 | strong similarity to UPD-GlcNAc transporter MNN2-2 - *Kluyveromyces lactis* |
| An04g00340 | strong similarity to myo-inositol transport protein Itr2 - *Saccharomyces cerevisiae* |
| An04g00350 | hypothetical protein |
| An04g00360 | strong similarity to transport vesicle formation protein Sec13 - *Saccharomyces cerevisiae* |
| An04g00500 | similarity to transcription activator prnA - *Aspergillus nidulans* |
| An04g00600 | strong similarity to hypothetical ER-to-Golgi transporter Lag1 - *Saccharomyces cerevisiae* |
| An04g00610 | strong similarity to hydroxymethylglutaryl-coenzyme A reductase hmg1p - *Schizosaccharomyces pombe* [truncated ORF] |
| An04g00630 | strong similarity to hypothetical protein CAC28557.1 - *Neurospora crassa* |
| An04g00670 | strong similarity to hypothetical coiled-coil protein similar to human congenital heart disease protein CAC05252.1 - *Schizosaccharomyces pombe* |
| An04g00970 | similarity to hypothetical transmembrane protein orf375 - *Bradyrhizobium japonicum* |
| An04g01260 | strong similarity to golgi alpha-1,6 mannosyltransferase subunit Anp1 - *Saccharomyces cerevisiae* |
| An04g01320 | strong similarity to fatty acid desaturase from patent WO9846764-A1 - *Homo sapiens* |
| An04g01390 | strong similarity to amino acid transporter ATA1 - *Homo sapiens* |
| An04g01600 | strong similarity to hypothetical protein B1D1.80 - *Neurospora crassa* |
| An04g01720 | similarity to DnaJ protein SIS1 - *Cryptococcus curvatus* |
| An04g01780 | strong similarity to hypothetical protein YAR002c-a - *Saccharomyces cerevisiae* |
| An04g01950 | strong similarity to zinc-metalloprotease Ste24 - *Saccharomyces cerevisiae* |
| An04g01990 | similarity to protein ZW10 homolog HZW10 - *Homo sapiens* |
| An04g02020 | strong similarity to cyclophilin cypB - *Aspergillus nidulans* |
| An04g02050 | similarity to hypoxia-induced protein #21 from patent WO200246465-A2 - *Homo sapiens* |
| An04g02240 | similarity to RanGTP-binding protein NXT1 - *Homo sapiens* |
| An04g02250 | questionable ORF |
| An04g02420 | strong similarity to ornithine decarboxylase ODC - *Paracoccidioides brasiliensis* [putative frameshift] |
| An04g02480 | strong similarity to lipid phosphoinositide phosphatase Sac1 - *Saccharomyces cerevisiae* |
| An04g02650 | strong similarity to cell cycle regulation protein-4 (CECRP-4) from patent WO9964593-A2 - *Homo sapiens* |
| An04g02870 | weak similarity to histone H1 - *Nicotiana tabacum* |
| An04g02880 | strong similarity to glutaminyl-peptide cyclotransferase QPCT - *Homo sapiens* |
| An04g02970 | similarity to dimethylaniline monooxygenase FMO - *Sus scrofa* |
| An04g03460 | phytase phyA - *Aspergillus niger* |
| An04g03580 | similarity to hypothetical protein BAA97223.1 - *Arabidopsis thaliana* |
| An04g03620 | similarity to 2-dehydro-3-deoxyphosphoheptonate aldolase - *Arabidopsis thaliana* |
| An04g03650 | hypothetical protein |
| An04g03870 | weak similarity to phosphatidic acid phosphatase ATPAP2 from patent WO200005385-A1 - *Arabidopsis thaliana* |
| An04g04080 | similarity to hypothetical negative acting factor related protein - *Neurospora crassa* |
| An04g04230 | hypothetical protein |
| An04g04240 | strong similarity to phosphate transport protein GvPT - *Glomus versiforme* |
| An04g04800 | strong similarity to hypothetical protein SPAC1486.02c - *Schizosaccharomyces pombe* [truncated ORF] |
| An04g05250 | strong similarity to undecaprenyl phosphate synthase Rer2 from patent WO200121650-A2 - *Saccharomyces cerevisiae* |
| An04g05310 | strong similarity to vacuolar H(+)-transporting ATPase subunit Vph1 - *Saccharomyces cerevisiae* |
| An04g05740 | similarity to arylamine N-acetyltransferase NAT1 - *Homo sapiens* |
| An04g05820 | similarity to transcription regulator Rfg1 - *Candida albicans* |
| An04g05840 | weak similarity to phospholipid-cholesterol acyltransferase - *Aeromonas hydrophila* |
| An04g05980 | strong similarity to v-SNARE Vti1 - *Saccharomyces cerevisiae* |
| An04g06310 | strong similarity to hypothetical protein CAB91735.2 - *Neurospora crassa* |
| An04g06760 | hypothetical protein |
| An04g06840 | similarity to ATPase Drs2 - *Saccharomyces cerevisiae* |
| An04g06960 | strong similarity to NADPH-ferrihemoprotein reductase 1 CPR1 - *Petroselinum crispum* |
| An04g06990 | strong similarity to alpha 1,2-mannosidase IC - *Homo sapiens* |
| An04g07000 | hypothetical protein |
| An04g07040 | strong similarity to clathrin light chain Clc1 - *Saccharomyces cerevisiae* [putative frameshift] |
| An04g07120 | similarity to protein FRM2 involved in fatty acid regulation - *Saccharomyces cerevisiae* |
| An04g07160 | similarity to hypothetical protein MLD14.3 - *Arabidopsis thaliana* |
| An04g07190 | similarity to hypothetical transcription regulator protein - *Schizosaccharomyces pombe* |
| An04g07240 | similarity to maltose pathway regulatory protein Mal13 - *Saccharomyces cerevisiae* |
| An04g07380 | strong similarity to hypothetical peptidase 48 family protein SPAP14E8.04 - *Schizosaccharomyces pombe* [truncated ORF] |
| An04g07430 | similarity to hypothetical protein encoded by An07g09430 - *Aspergillus niger* |
| An04g08580 | strong similarity to translation initiation factor eIF-2 alpha chain kinase HRI - *Oryctolagus cuniculus* |
| An04g08590 | strong similarity to DNA photolyase phr1 - *Trichoderma harzianum* |
| An04g08690 | similarity to polynucleotide sequence SEQ ID NO:3913 from patent WO200058473-A2 - *Homo sapiens* |
| An04g08820 | strong similarity to glucosyltransferase Alg8 - *Saccharomyces cerevisiae* |
| An04g08830 | similarity to Golgi membrane protein Emp47 - *Saccharomyces cerevisiae* |
| An04g09020 | similarity to twinfilin A Twf1 - *Saccharomyces cerevisiae* |
| An04g10140 | hypothetical protein |
| An05g00130 | similarity to cell wall synthesis protein KRE9 - *Candida albicans* |
| An05g00140 | similarity to signal recognition particle receptor beta chain Srp102 - *Saccharomyces cerevisiae* |
| An05g00200 | strong similarity to ankyrin repeat-containing protein Akr1 - *Saccharomyces cerevisiae* |
| An05g00880 | strong similarity to dnaJ protein homolog Scj1 - *Saccharomyces cerevisiae* |
| An05g01350 | hypothetical protein |
| An05g01890 | strong similarity to hypothetical protein related to host-specific AK-toxin Akt2 B23L21.350 - *Neurospora crassa* |
| An05g02360 | strong similarity to hypothetical protein encoded by An18g01530 - *Aspergillus niger* |
| An06g01100 | strong similarity to mannosyltransferase Alg1 - *Saccharomyces cerevisiae* |
| An06g01200 | strong similarity to endosomal protein Emp70 - *Saccharomyces cerevisiae* |
| An06g01630 | strong similarity to hypothetical protein KIAA1134 - *Homo sapiens* |
| An06g01720 | strong similarity to Ariadne protein ari - *Drosophila melanogaster* |
| An06g01810 | strong similarity to protein PL6 - *Homo sapiens* |
| An07g01800 | hypothetical protein |
| An07g01970 | strong similarity to cytoplasmic metalloproteinase mepB - *Aspergillus fumigatus* |
| An07g02060 | similarity to myo-inositol transporter 2 - *Schizosaccharomyces pombe* |
| An07g02150 | similarity to hypothetical AAA family ATPase Cj0377 - *Campylobacter jejuni* |
| An07g02170 | similarity to transport protein Bos1 - *Saccharomyces cerevisiae* |
| An07g02250 | strong similarity to hypothetical DNA methylase SPAC27D7.08c - *Schizosaccharomyces pombe* |
| An07g02380 | weak similarity to T-2 toxin biosynthesis protein TRI7 - *Fusarium sporotrichioides* |
| An07g03050 | similarity to hypothetical integral membrane protein SCC53.26c - *Streptomyces coelicolor* |
| An07g03130 | strong similarity to mitochondrial cation transporter Mmt1 - *Saccharomyces cerevisiae* |
| An07g03200 | strong similarity to adaptor complex AP-1 medium chain AP47 - *Mus musculus* |
| An07g03380 | similarity to UDP-glucose ceramide glucosyltransferase UGCG - *Mus musculus* |
| An07g03410 | similarity to hypothetical membrane protein YDL237w - *Saccharomyces cerevisiae* |
| An07g03420 | similarity to hypothetical protein SPAC17A5.16 - *Schizosaccharomyces pombe* |
| An07g03440 | similarity to hypothetical protein YHR045w - *Saccharomyces cerevisiae* |
| An07g03680 | strong similarity to repressor protein qutR - *Aspergillus nidulans* |
| An07g03690 | strong similarity to amino acid transporter ata2 - *Homo sapiens* |
| An07g03760 | strong similarity to 100 kDa coactivator snd1 - *Homo sapiens* |
| An07g03880 | serine proteinase pepC - *Aspergillus niger* |
| An07g03880 | serine proteinase pepC - *Aspergillus niger* |
| An07g04190 | strong similarity to dolichyl-diphosphooligosaccharide--protein glycosyltransferase 48kD chain DDOST - *Gallus gallus* |
| An07g04730 | strong similarity to gene #6 associated peptide #4 from patent WO200107459-A1 - *Homo sapiens* |
| An07g04880 | weak similarity to translation initiation factor eIF-2 alpha chain kinase HCR - *Rattus norvegicus* |
| An07g04940 | strong similarity to alpha-1,6-mannosyltransferase Hoc1 - *Saccharomyces cerevisiae* |
| An07g05520 | strong similarity to O-methyltransferase B omtB - *Aspergillus parasiticus* |
| An07g05620 | strong similarity to transcription elongation factor Rtf1 - *Saccharomyces cerevisiae* |
| An07g05620 | strong similarity to transcription elongation factor Rtf1 - *Saccharomyces cerevisiae* |
| An07g05670 | strong similarity to hypothetical membrane protein YOR389w - *Saccharomyces cerevisiae* |
| An07g06030 | strong similarity to coatomer gamma subunit 2 copg2 - *Homo sapiens* |
| An07g06150 | strong similarity to lanosterol synthase erg7p - *Schizosaccharomyces pombe* |
| An07g06190 | similarity to hypothetical related to COP1-interacting protein CIP8 - *Neurospora crassa* |
| An07g06230 | hypothetical protein |
| An07g06280 | strong similarity to maleylacetoacetate isomerase maiA - *Aspergillus nidulans* |
| An07g06430 | strong similarity to glycoprotein glucosyltransferase gpt1p - *Schizosaccharomyces pombe* |
| An07g06770 | strong similarity to delta-6 desaturase - *Mucor rouxii* |
| An07g06990 | strong similarity to hypothetical protein SPCC594.04c - *Schizosaccharomyces pombe* |
| An07g07340 | strong similarity to luminal ER-protein retention receptor ERD2 - *Kluyveromyces marxianus* |
| An07g07500 | strong similarity to ABC-type vacuolar membrane protein hmt1p - *Schizosaccharomyces pombe* |
| An07g08220 | strong similarity to clathrin associated epsin 2A - *Homo sapiens* |
| An07g08350 | strong similarity to hypothetical protein YPL067c - *Saccharomyces cerevisiae* |
| An07g08510 | hypothetical protein |
| An07g08830 | strong similarity to adaptor protein Yap180 - *Saccharomyces cerevisiae* |
| An07g09170 | strong similarity to breast cancer resistance protein 1 BCRP1 - *Mus musculus* |
| An07g09350 | strong similarity to hypothetical protein Rv0303 - *Mycobacterium tuberculosis* |
| An07g09570 | strong similarity to phosphatidate cytidylyltransferase Cds1 - *Saccharomyces cerevisiae* |
| An07g09610 | strong similarity to hypothetical protein B24H17.130 - *Neurospora crassa* |
| An07g09650 | similarity to chitin synthase III like Chs7 - *Saccharomyces cerevisiae* [truncated ORF] |
| An07g09690 | strong similarity to sterol C-24(28) reductase sts1p - *Schizosaccharomyces pombe* |
| An07g09840 | similarity to tetracyclin resistance protein tetA - *Escherichia coli* |
| An07g10280 | strong similarity to hypothetical protein B17C10.170 - *Neurospora crassa* |
| An07g10350 | protein O-mannosyl transferase pmtA - *Aspergillus niger* |
| An07g10370 | strong similarity to high-affinity glucose transporter HGT1 - *Kluyveromyces lactis* |
| An07g10420 | strong similarity to cell division cycle protein Cdc50 - *Saccharomyces cerevisiae* |
| An08g00290 | strong similarity to golgin-160 related protein Rud3 - *Saccharomyces cerevisiae* |
| An08g00430 | strong similarity to precursor of carboxypeptidase Kex1 - *Saccharomyces cerevisiae* |
| An08g00560 | strong similarity to phosphatidyl-N-methylethanolamine N-methyltransferase Opi3 - *Saccharomyces cerevisiae* |
| An08g00750 | strong similarity to hypothetical conserved protein SPBP35G2.02 - *Schizosaccharomyces pombe* |
| An08g00810 | similarity to tetracyclin resistance protein TetH - *Pasteurella multocida* |
| An08g00900 | strong similarity to hypothetical protein BAB22431.1 - *Mus musculus* [putative frameshift] |
| An08g01030 | strong similarity to vacuolar protein sorting protein Vps29 - *Saccharomyces cerevisiae* |
| An08g01210 | strong similarity to hypothetical 105-kDa kinase-like protein - *Mus musculus* |
| An08g01250 | weak similarity to COP1-interacting protein 7 CIP7 - *Arabidopsis thaliana* |
| An08g01360 | similarity to hypothetical protein G11A3.030 - *Neurospora crassa* |
| An08g01410 | strong similarity to ER-Golgi transport protein Sft2 - *Saccharomyces cerevisiae* |
| An08g01440 | similarity to hypothetical protein SCD19.11 - *Streptomyces coelicolor* |
| An08g01480 | strong similarity to tRNA ligase Trl1 - *Saccharomyces cerevisiae* |
| An08g03000 | strong similarity to H+-transporting ATPase chain C Vma5 - *Saccharomyces cerevisiae* |
| An08g03270 | strong similarity to beta-COP Sec26 - *Saccharomyces cerevisiae* |
| An08g03570 | similarity to slow myosin heavy chain MyHC3 - *Coturnix Coturnix* |
| An08g03590 | strong similarity to precursor of protein Emp24 - *Saccharomyces cerevisiae* |
| An08g03640 | strong similarity to HC-toxin efflux pump TOXA - *Cochliobolus carbonum* |
| An08g03960 | strong similarity to hypothetical edoplasmic reticulum associated protein - *Schizosaccharomyces pombe* |
| An08g03970 | strong similarity to hypothetical protein CAE47902.1 - *Aspergillus fumigatus* |
| An08g04000 | strong similarity to hypothetical transcription factor CAE47904.1 - *Aspergillus fumigatus* |
| An08g04120 | similarity to hypothetical mold-specific protein MS8 - *Ajellomyces capsulatus* |
| An08g04260 | similarity to hypothetical protein YNL191w - *Saccharomyces cerevisiae* |
| An08g04400 | similarity to membrane protein Zrg17 - *Saccharomyces cerevisiae* |
| An08g04430 | similarity to cap-binding protein 4EHP - *Homo sapiens* |
| An08g04450 | similarity to hypothetical guanosine-diphosphatase - *Schizosaccharomyces pombe* |
| An08g04870 | similarity to beta transducin-like protein het-e1 - *Podospora anserina* |
| An08g04990 | strong similarity to carnitine acetyl transferase facC - *Aspergillus nidulans* |
| An08g05570 | similarity to secretory protein Sec5 - *Saccharomyces cerevisiae* |
| An08g06030 | strong similarity to hypothetical zinc-finger transcription factor CAD28447.1 - *Aspergillus fumigatus* |
| An08g06200 | similarity to mitochondrial import protein Metaxin - *Homo sapiens* |
| An08g06250 | strong similarity to benzoate 4-monooxygenase cytochrome P450 53 bphA - *Aspergillus niger* |
| An08g06270 | similarity to adenosine deaminase - *Mus musculus* |
| An08g06330 | strong similarity to epsilon-COP - *Cricetulus griseus* |
| An08g06420 | similarity to hypothetical protein SPBC405.03c - *Schizosaccharomyces pombe* |
| An08g06750 | strong similarity to regulator protein Rav1 - *Saccharomyces cerevisiae* |
| An08g06780 | strong similarity to transport protein Uso1 - *Saccharomyces cerevisiae* |
| An08g07020 | similarity to mannosyl transferase Alg9 - *Saccharomyces cerevisiae* |
| An08g07090 | similarity to protein Sim1 - *Saccharomyces cerevisiae* |
| An08g07560 | similarity to hypothetical protein CAD37151.1 - *Aspergillus fumigatus* |
| An08g08020 | similarity to bialaphos acetylhydrolase bah - *Streptomyces hygroscopicus* |
| An08g08090 | similarity to hypothetical protein CAD21290.1 - *Neurospora crassa* |
| An08g09000 | strong similarity to ubiquitin like protein Dsk2 - *Saccharomyces cerevisiae* |
| An08g09430 | similarity to notchless Nle - *Drosophila melanogaster* |
| An08g09500 | similarity to kinesin light chain - Loligo pealei |
| An08g10570 | strong similarity to hypothetical membrane protein YLR386w - *Saccharomyces cerevisiae* |
| An08g10650 | strong similarity to transport protein Sec24 - *Saccharomyces cerevisiae* |
| An08g10730 | similarity to hypothetical protein SPAC19B12.06c - *Schizosaccharomyces pombe* |
| An08g10750 | strong similarity to hypothetical protein SPBC3B8.06 - *Schizosaccharomyces pombe* |
| An08g11120 | weak similarity to protein PRO4400 from patent WO200073348-A2 - *Homo sapiens* |
| An09g00580 | similarity to hypothetical transcription activator SPAC139.03 - *Schizosaccharomyces pombe* |
| An09g00620 | similarity to estradiol 17-beta-dehydrogenase HSD17B1 - *Rattus norvegicus* |
| An09g00630 | strong similarity to hypothetical cipC - *Aspergillus nidulans* |
| An09g00650 | similarity to hypothetical protein F9K20.18 - *Arabidopsis thaliana* |
| An09g00670 | strong similarity to glycosylphosphatidylinositol-anchored beta(1-3)glucanosyltransferase gel3 - *Aspergillus fumigatus* |
| An09g01240 | strong similarity to phospholipase B - *Penicillium notatum* |
| An09g01710 | similarity to epoxide hydrolase from patent EP879890-A - *Grobacterium radiobacter* |
| An09g02530 | strong similarity to hypothetical protein EAA60542.1 - *Aspergillus nidulans* |
| An09g03210 | strong similarity to isobutene-forming enzyme and benzoate 4-hydroxylase P450rm - *Rhodotorula minuta* |
| An09g04170 | strong similarity to protein Sly1 - *Saccharomyces cerevisiae* |
| An09g04580 | strong similarity to palmitoyl-protein thioesterase - *Rattus norvegicus* |
| An09g04660 | strong similarity to meiotic mRNA stability protein kinase Ume5 - *Saccharomyces cerevisiae* |
| An09g04680 | similarity to hepatic glucose transport protein GLUT2 - *Rattus norvegicus* |
| An09g04880 | hypothetical protein |
| An09g05420 | similarity to signal peptidase subunit Spc3 - *Saccharomyces cerevisiae* |
| An09g05490 | strong similarity to COP-coated vesicle membrane protein P24 homolog lbrA - *Polysphondylium pallidum* |
| An09g05600 | similarity to hypothetical protein CAD11369.1 - *Neurospora crassa* |
| An09g05880 | strong similarity to alpha-glucosidase ModA - *Dictyostelium discoideum* |
| An09g06130 | similarity to hypothetical protein CAD21257.1 - *Neurospora crassa* |
| An09g06260 | similarity to mutanase mutA - *Aspergillus oryzae* |
| An09g06340 | strong similarity to immunogenic protein #11392 from patent WO200181581-A2 - Propionibacterium acnes |
| An09g06510 | strong similarity to human transmembrane from patent WO200056891-A2 - *Homo sapiens* |
| An10g00340 | similarity to PTH11 - *Magnaporthe grisea* |
| An11g00010 | similarity to antiviral GTPase Mx1 - *Oncorhynchus mykiss* |
| An11g00220 | similarity to ferric reductase cfl1 - *Candida albicans* |
| An11g01190 | similarity to protein participating in extracellular/cell surface phenomena Ecm33 - *Saccharomyces cerevisiae* |
| An11g01240 | similarity to filamentous growth protein Dfg5 - *Saccharomyces cerevisiae* |
| An11g01260 | weak similarity to hypothetical protein encoded by B1D4.080 - *Neurospora crassa* |
| An11g01270 | similarity to amino acid permease Mmp1 - *Saccharomyces cerevisiae* |
| An11g01550 | strong similarity to cytochrome P450 52A4 - *Candida maltosa* |
| An11g02020 | similarity to glucose-6-phosphate/phosphate-translocator GPT - *Zea mays* [putative sequencing error] |
| An11g02090 | strong similarity to hypothetical protein CAD70545.1 - *Neurospora crassa* |
| An11g02220 | similarity to hypothetical protein SPBC18H10.09 - *Schizosaccharomyces pombe* |
| An11g02230 | strong similarity to lanosterol 14 alpha-demethylase (P450(14DM)) CYP51 - *Penicillium italicum* |
| An11g02380 | strong similarity to GTP:alpha-D-mannose-1-phosphate guanylyltransferase MPG1 - *Hypocrea jecorina* |
| An11g02760 | strong similarity to protein involved in vesicle transport in the secretory pathway YAL048c - *Saccharomyces cerevisiae* |
| An11g02840 | strong similarity to hypothetical protein SPAC637.13c - *Schizosaccharomyces pombe* |
| An11g02990 | strong similarity to astaxanthin synthetase from patent EP1035206 - *Phaffia rhodozyma* |
| An11g03060 | strong similarity to salicylate hydroxylase sal - *Pseudomonas putida* |
| An11g03460 | strong similarity to vegetative incompatibility factor het-e1 - *Podospora anserina* |
| An11g03570 | strong similarity to cytochrome P450 monooxygenase TRI4 - *Fusarium sporotrichioides* |
| An11g03760 | strong similarity to hypothetical protein BAC67939.1 - *Streptomyces avermitilis* |
| An11g04180 | dnaK-type molecular chaperone bipA - *Aspergillus niger* |
| An11g04750 | strong similarity to developmental regulator of asexual and sexual reproduction dopA - *Aspergillus nidulans* |
| An11g04830 | strong similarity to hypothetical protein EAA64933.1 - *Aspergillus nidulans* |
| An11g04880 | weak similarity to hypothetical novel nuclear protein NNP-1 var - *Mus musculus* |
| An11g05110 | similarity to death-associated protein kinase DAPK1 - *Homo sapiens* |
| An11g05300 | strong similarity to transposase of transposable element Tan1 - *Aspergillus niger* [putative pseudogene] |
| An11g05330 | strong similarity to diacylglycerol pyrophosphate phosphatase Dpp1 - *Saccharomyces cerevisiae* |
| An11g05460 | strong similarity to F-actin capping protein alpha-1 subunit CapZ - *Mus musculus* [truncated ORF] |
| An11g05600 | strong similarity to alcohol dehydrogenase alkJ - *Pseudomonas oleovorans* |
| An11g05650 | similarity to golgi peripheral membrane protein p65 GRASP65 - *Rattus norvegicus* |
| An11g05680 | similarity to cytochrome P450 3A13 - *Mus musculus* |
| An11g05970 | weak similarity to muramidase-2 - *Enterococcus hirae* |
| An11g06040 | similarity to choline kinase CK1 - *Glycine max* |
| An11g06420 | strong similarity to sequence 253 from patent WO0100804 - *Corynebacterium glutamicum* |
| An11g06480 | weak similarity to antigenic protein f86.aa from patent WO9859071 - *Borrelia burgdorferi* |
| An11g06770 | strong similarity to skin cell protein from patent WO9955865-A1 - *Rattus sp.* |
| An11g06960 | strong similarity to aminopeptidase II pepP - *Escherichia coli* |
| An11g07530 | similarity to protein Notchless - *Drosophila melanogaster* |
| An11g07610 | weak similarity to transcriptional activator Mut3 - *Pichia angusta* |
| An11g07660 | similarity to exo-1,3-beta-glucanase Xog - *Candida ablicans* |
| An11g07720 | hypothetical protein |
| An11g07780 | strong similarity to multidrug resistance-associated protein Mrp2 - *Oryctolagus cuniculus* |
| An11g08010 | similarity to hypothetical protein SPCC1281.02c - *Schizosaccharomyces pombe* |
| An11g08020 | strong similarity to G-11 integral membrane protein PTH11 - *Magnaporthe grisea* |
| An11g08030 | strong similarity to hypothetical sterigmatocystin biosythesis monooxygenase stcW - *Aspergillus nidulans* |
| An11g08080 | strong similarity to cDNA O-methyltransferase mt-I - *Aspergillus parasiticus* |
| An11g09430 | weak similarity to hypothetical protein encoded by An03g01410 - *Aspergillus niger* |
| An11g09890 | strong similarity to mannosyltransferase 1 PMT1 - *Candida albicans* |
| An11g10080 | weak similarity to hypothetical protein CAE76369.1 - *Neurospora crassa* |
| An11g10210 | similarity to anaphase-promoting complex subunit APC5 - *Homo sapiens* |
| An11g10330 | strong similarity to hypothetical membrane protein YPR091c - *Saccharomyces cerevisiae* |
| An11g10870 | strong similarity to gene expression regulator An13 from patent WO200257456-A2 - Unclassified organism |
| An11g10950 | strong similarity to unc-50 related protein UNCL - *Rattus norvegicus* |
| An11g11180 | strong similarity to hypothetical protein encoded by SPBC1198.08 - *Schizosaccharomyces pombe* |
| An11g11240 | strong similarity to translation releasing factor RF-1 precursor MRF1 - *Kluyveromyces lactis* |
| An11g11250 | strong similarity to interferon-induced double-stranded RNA-activated protein kinase inhibitor P58 - *Homo sapiens* |
| An12g00120 | strong similarity to hypothetical oxysterol binding protein homologue Swh1 - *Saccharomyces cerevisiae* |
| An12g00140 | weak similarity to mucin-like protein Muc1 - *Saccharomyces cerevisiae* |
| An12g00340 | similarity to alpha 1,2-mannosidase IB - *Homo sapiens* |
| An12g00580 | weak similarity to heat shock protein dnaJ - *Brucella ovis* |
| An12g00630 | strong similarity to triacylglycerol lipase Tgl2 - *Saccharomyces cerevisiae* |
| An12g00800 | strong similarity to C2-domain family vesicle protein GLUT4 - *Rattus norvegicus* |
| An12g00980 | similarity to hypothetical protein encoded by An18g01730 - *Aspergillus niger* |
| An12g01360 | strong similarity to hypothetical cytochrome P450 protein 2E4.050 - *Neurospora crassa* |
| An12g01640 | weak similarity to mucin - *Sus scrofa* |
| An12g01650 | similarity to polypeptide SEQ ID NO 29028 from patent WO200171042-A2 - *Drosophila melanogaster* |
| An12g02080 | strong similarity to cytochrome P450 monooxygenase stcS - *Aspergillus nidulans* [putative frameshift] |
| An12g02180 | similarity to hypothetical protein encoded by An12g09970 - *Aspergillus niger* |
| An12g02190 | strong similarity to hypothetical protein encoded by An12g09960 - *Aspergillus niger* |
| An12g02450 | strong similarity to alpha-glucan synthase mok1p - *Schizosaccharomyces pombe* |
| An12g03150 | strong similarity to multidrug resistance protein atrD - *Aspergillus nidulans* |
| An12g03500 | weak similarity to hypothetical protein CAD71089.1 - *Neurospora crassa* |
| An12g03580 | strong similarity to microsomal glutathione S-transferase 3 MGST3 - *Homo sapiens* |
| An12g03880 | similarity to hypothetical protein SPCC4B3.12 - *Schizosaccharomyces pombe* |
| An12g03980 | strong similarity to hypothetical protein AAN52526.1 - *Pichia angusta* |
| An12g04050 | similarity to hypothetical protein b1011 - *Escherichia coli* |
| An12g04510 | strong similarity to benzoate 4-monooxygenase cytochrome P450 53 bphA - *Aspergillus niger* |
| An12g04830 | strong similarity to coatomer protein zeta chain Ret3 - *Saccharomyces cerevisiae* |
| An12g04880 | hypothetical protein |
| An12g05170 | similarity to hypothetical protein AAM35689.1 - *Xanthomonas axonopodis* |
| An12g05360 | strong similarity to cholesterol 24-hydroxylase - *Mus musculus* |
| An12g07770 | similarity to protein fragment SEQ ID NO:6347 from patent EP1033405-A2 - *Arabidopsis thaliana* |
| An12g07940 | similarity to hypothetical protein Y48E1B.1 - *Caenorhabditis elegans* [truncated ORF] |
| An12g08560 | strong similarity to proteinase SlpE - *Streptomyces lividans* |
| An12g08700 | hypothetical protein |
| An12g08750 | similarity to hypothetical protein At2g21120 - *Arabidopsis thaliana* |
| An12g08890 | similarity to hypothetical protein EAA72664.1 - *Gibberella zeae* |
| An12g09780 | weak similarity to alkyl salicylate esterase salE - *Acinetobacter sp*. |
| An12g10280 | similarity to hypothetical protein 1A9.40 - *Neurospora crassa* |
| An12g10330 | weak similarity to hypothetical protein encoded by An02g14500 - *Aspergillus niger* [truncated ORF] |
| An12g10380 | strong similarity to chitin synthase C chsC - *Aspergillus fumigatus* |
| An12g10830 | similarity to hypothetical protein EAA74834.1 - *Gibberella zeae* |
| An12g10840 | hypothetical protein |
| An13g00040 | strong similarity to 1-acylglycerol-3-phosphate O-acyltransferase from patent WO200001713-A2 - Mortierella ramanniana |
| An13g00110 | strong similarity to 1-phosphatidylinositol 4-kinase Stt4 - *Saccharomyces cerevisiae* |
| An13g00140 | similarity to splicing factor YT521 - *Rattus norvegicus* |
| An13g00300 | strong similarity to hypothetical protein B14D6.440 - *Neurospora crassa* |
| An13g00620 | strong similarity to 80K protein H precursor G19P1 - *Homo sapiens* |
| An13g00740 | similarity to cyclopropane-fatty-acyl-phospholipid synthase - *Escherichia coli* |
| An13g00780 | strong similarity to hypothetical protein CAD21419.1 - *Neurospora crassa* |
| An13g01280 | strong similarity to hypothetical protein EAA64035.1 - *Aspergillus nidulans* |
| An13g01300 | strong similarity to gamma-glutamyl transpeptidase GGT1 - *Homo sapiens* |
| An13g01960 | strong similarity to alcohol dehydrogenase alkJ - *Pseudomonas putida* |
| An13g02410 | weak similarity to hypothetical cation transporter DRA0361 - *Deinococcus radiodurans* |
| An13g02990 | similarity to hypothetical protein EAA71271.1 - *Gibberella zeae* |
| An13g04060 | strong similarity to hypothetical protein encoded by An07g08820 - *Aspergillus niger* |
| An13g04070 | strong similarity to FLO11 gene expression regulator At14 from patent WO200257456-A2 - Unclassified organism |
| An13g04080 | strong similarity to cytochrome P450 monooxygenase P450II - *Gibberella fujikuroi* |
| An14g00150 | similarity to hypothetical protein B24H17.110 - *Neurospora crassa* |
| An14g00190 | weak similarity to hypothetical protein At2g17590 - *Arabidopsis thaliana* |
| An14g00210 | similarity to Golgi membrane protein Yip1 - *Saccharomyces cerevisiae* |
| An14g00270 | weak similarity to dolichol-phosphate-mannose synthase DPM3 - *Homo sapiens* |
| An14g00620 | strong similarity to aminopeptidase from patent WO9628542-A1 - *Aspergillus oryzae* |
| An14g00870 | weak similarity to hypothetical protein K06A9.1a - *Caenorhabditis elegans* |
| An14g00900 | strong similarity to glycosylphosphatidylinositol anchor synthesis protein Mcd4 - *Saccharomyces cerevisiae* |
| An14g01170 | similarity to hypothetical protein B7F18.110 - *Neurospora crassa* |
| An14g01180 | similarity to hypothetical protein encoded by An02g08790 - *Aspergillus niger* |
| An14g01440 | strong similarity to succinate-semialdehyde dehydrogenase SSADH - *Rattus norvegicus* |
| An14g01630 | similarity to nitrosoguanidine resistance factor Sng1 - *Saccharomyces cerevisiae* |
| An14g01730 | strong similarity to hypothetical DEAD/DEAH-box RNA helicase SPAC694.02 - *Schizosaccharomyces pombe* |
| An14g02200 | strong similarity to EST an_2365 - *Aspergillus niger* |
| An14g02280 | similarity to hypothetical protein encoded by An02g01160 - *Aspergillus niger* |
| An14g02990 | similarity to hypothetical protein KIAA1715 - *Homo sapiens* |
| An14g03360 | strong similarity to choline-transport mutant SCT1 supressor protein - *Saccharomyces cerevisiae* |
| An14g03910 | strong similarity to alpha-1,2-mannosyltransferase kre2 - *Candida albicans* |
| An14g04040 | strong similarity to hypothetical protein SPAC1093.01 with conserved domain PF01535 duf17p - *Schizosaccharomyces pombe* |
| An14g04040 | strong similarity to hypothetical protein SPAC1093.01 with conserved domain PF01535 duf17p - *Schizosaccharomyces pombe* |
| An14g04490 | weak similarity to hypothetical protein AF1318 - *Archaeoglobus fulgidus* |
| An14g04970 | weak similarity to hypothetical zinc-finger protein SPAC19A8.10 - *Schizosaccharomyces pombe* |
| An14g05100 | strong similarity to hypothetical protein alr2987 - Nostoc sp. |
| An14g05150 | similarity to ubiquitin specific protease Ubp1 - *Saccharomyces cerevisiae* |
| An14g05410 | strong similarity to hypothetical protein CAD21189.1 - *Neurospora crassa* |
| An14g05910 | strong similarity to mannosyltransferase Alg2 - *Saccharomyces cerevisiae* |
| An14g06060 | strong similarity to alpha-1,2-mannosyltransferase Ttp1 - *Saccharomyces cerevisiae* |
| An14g06440 | strong similarity to protein of sequence 1 from patent WO0075305 - *Candida albicans* |
| An14g06550 | strong similarity to hypothetical membrane protein YNL011c - *Saccharomyces cerevisiae* |
| An14g06610 | strong similarity to GTP-binding protein GTPBP1 - *Homo sapiens* |
| An14g06640 | strong similarity to N-acetylglucosaminyl-phosphatidylinositol deacetylase PIGL - *Rattus norvegicus* |
| An14g06830 | strong similarity to hypothetical membrane protein Ptm1 - *Saccharomyces cerevisiae* |
| An14g07030 | strong similarity to p-nitrobenzyl esterase pnbA - *Bacillus subtilis* |
| An15g00520 | strong similarity to hypothetical membrane protein YJR044c - *Saccharomyces cerevisiae* |
| An15g00540 | strong similarity to hypothetical protein B13O20.120 - *Neurospora crassa* |
| An15g00630 | strong similarity to sphingolipid metabolism Sur1 - *Saccharomyces cerevisiae* |
| An15g00640 | strong similarity to hypothetical protein GABA-A receptor epsilon subunit - *Caenorhabditis elegans* |
| An15g01300 | weak similarity to suppressor of telomeric repression-7 STR7 from patent WO9612811-A2 - *Saccharomyces cerevisiae* |
| An15g01350 | strong similarity to hypothetical cleft lip and palate transmembrane protein 1 CLPTM1 - *Homo sapiens* |
| An15g01380 | strong similarity to Synaptobrevin homolog v-SNARE Sec22 - *Saccharomyces cerevisiae* |
| An15g01420 | strong similarity to glucosidase I Cwh41 - *Saccharomyces cerevisiae* |
| An15g01460 | strong similarity to Cwh8 - *Saccharomyces cerevisiae* |
| An15g01510 | strong similarity to P-type ATPase Drs2 - *Saccharomyces cerevisiae* |
| An15g01520 | strong similarity to multidomain vesicle coat protein Sec16 - *Saccharomyces cerevisiae* |
| An15g01680 | strong similarity to signal peptide-containing protein SIGP from patent WO9933981-A2 - *Homo sapiens* |
| An15g02310 | hypothetical protein |
| An15g02400 | similarity to hypothetical protein CG11840 - *Drosophila melanogaster* |
| An15g02680 | strong similarity to tetracyclin resistance protein tetA - *Escherichia coli* |
| An15g02760 | strong similarity to protein involved in actin distribution and bipolar budding Rsv167 - *Saccharomyces cerevisiae* |
| An15g02930 | strong similarity to ABC transporter CDR4 - *Candida albicans* |
| An15g02960 | similarity to hypothetical protein CAD37071.1 - *Neurospora crassa* |
| An15g04370 | similarity to proline dipeptidase from patent JP11318454-A - *Microbacterium esteraromaticum* |
| An15g04380 | strong similarity to phenylacetate 2-hydroxylase phacA - *Aspergillus nidulans* |
| An15g04490 | strong similarity to myosin I myoA - *Aspergillus nidulans* |
| An15g04690 | similarity to protein fragment SEQ ID NO:49734 from patent EP1033405-A2 - *Arabidopsis thaliana* |
| An15g05080 | weak similarity to hypothetical protein CAD21084.1 - *Neurospora crassa* |
| An15g05090 | strong similarity to polyketide synthase FUM5 - *Gibberella moniliformis* |
| An15g05120 | similarity to alkaline ceramidase Ypc1 - *Saccharomyces cerevisiae* |
| An15g05650 | strong similarity to SR-protein-specific serine kinase SRPK2 - *Homo sapiens* |
| An15g05840 | similarity to hypothetical protein CAC28841.2 - *Neurospora crassa* |
| An15g05960 | weak similarity to hypothetical protein AAF54945.1 - *Drosophila melanogaster* |
| An15g06230 | strong similarity to aminonitrophenyl propanediol resistance protein Anp1 - *Saccharomyces cerevisiae* |
| An15g06820 | strong similarity to hypothetical protein SPAC3H5.09c - *Schizosaccharomyces pombe* |
| An15g06890 | hypothetical protein [truncated ORF] |
| An15g06890 | hypothetical protein [truncated ORF] |
| An15g06910 | similarity to educed expression in colorectal cancer protein rec - *Homo sapiens* |
| An15g07720 | strong similarity to carboxyphosphonoenolpyruvate mutase - *Streptomyces hygroscopicus* |
| An15g07730 | strong similarity to 3-isopropylmalate dehydratase leu1 - *Rhizopus niveus* |
| An16g00010 | similarity to alcohol dehydrogenase orfB from patent WO9807867-A2 - *Lactococcus lactis* |
| An16g01200 | strong similarity to choline permease Hnm1 - *Saccharomyces cerevisiae* |
| An16g01290 | strong similarity to hypothetical protein encoded by An02g05930 - *Aspergillus niger* |
| An16g01550 | strong similarity to hypothetical protein B23I11.160 - *Neurospora crassa* |
| An16g01820 | strong similarity to hypothetical inorganic phosphate transporter and regulator of Pho81p Pho88 - *Saccharomyces cerevisiae* |
| An16g01890 | strong similarity to hypothetical protein encoded by An14g02280 - *Aspergillus niger* |
| An16g02040 | strong similarity to Cys2-His2 zinc finger transcription factor ACEI - *Hypocrea jecorina* |
| An16g02460 | strong similarity to alpha subunit of the coatomer complex Ret1 - *Saccharomyces cerevisiae* |
| An16g02500 | strong similarity to tryptophan synthase trpB - *Aspergillus nidulans* |
| An16g03320 | strong similarity to transport protein Sec24A - *Homo sapiens* |
| An16g03450 | similarity to hypothetical protein YNL051w - *Saccharomyces cerevisiae* |
| An16g03530 | similarity to n-acetylglucosaminyl-phosphatidylinositol biosynthetic protein gpi1p - *Schizosaccharomyces pombe* |
| An16g04330 | strong similarity to mannose phospho-dolichol synthase dpm1 - *Hypocrea jecorina* |
| An16g04640 | strong similarity to hypothetical protein SPCC1281.03c - *Schizosaccharomyces pombe* |
| An16g05370 | similarity to zinc-finger protein Glo3 - *Saccharomyces cerevisiae* |
| An16g06350 | strong similarity to delta(6)-desaturase - *Mucor rouxii* |
| An16g06750 | similarity to D-stereospecific aminopeptidase - *Ochrobactrum anthropi* |
| An16g06890 | weak similarity to fibrinogen-binding protein - *Staphylococcus aureus* |
| An16g07180 | strong similarity to vanillin dehydrogenase VDH from patent EP0845532 - Unclassified organism |
| An16g07380 | strong similarity to hypothetical cDNA MGC:7080 - *Mus musculus* |
| An16g07390 | strong similarity to endoplasmatic reticulum signal peptidase subunit Spc2 - *Saccharomyces cerevisiae* |
| An16g07620 | strong similarity to endoplasmatic reticulum oxidising protein Ero1 - *Saccharomyces cerevisiae* |
| An16g07970 | similarity to autocrine motility factor receptor Amfr - *Mus musculus* |
| An16g08090 | strong similarity to hypothetical protein B2J23.120 - *Neurospora crassa* |
| An16g08140 | similarity to salicylate hydroxylase sal - *Pseudomonas putida* |
| An16g08180 | similarity to palmitylated serine/threonine kinase PKL12 - *Mus musculus* [putative frameshift] |
| An16g08420 | similarity to hypothetical integral membrane protein SC10B7.28 - *Streptomyces coelicolor* |
| An16g08470 | similarity to hypothetical cell growth regulator OS-9 - *Homo sapiens* |
| An16g08490 | strong similarity to dolichyl-phosphate-D-mannose--protein O-mannosyltransferase Pmt4 - *Saccharomyces cerevisiae* |
| An16g08570 | strong similarity to translation initiation factor 3 47 kDa subunit stt3p - *Schizosaccharomyces pombe* |
| An16g08610 | similarity to trichothecene 3-O-acetyltransferase TRI101 - *Fusarium sporotrichioides* |
| An16g08830 | strong similarity to component of ER protein-translocation subcomplex Sec71 from patent WO9949028-A1 - *Saccharomyces cerevisiae* |
| An16g09150 | strong similarity to hypothetical protein BM-021 - *Homo sapiens* |
| An16g09180 | strong similarity to low affinity zinc transport protein Zrt2 - *Saccharomyces cerevisiae* |
| An16g09300 | weak similarity to RING finger protein ReMembR-H2 - *Arabidopsis thaliana* |
| An17g00090 | weak similarity to translocation protein Sec72 - *Saccharomyces cerevisiae* |
| An17g00390 | strong similarity to aminopeptidase from patent WO9628542-A1 - *Aspergillus oryzae* |
| An17g00550 | weak similarity to dTDP-glucose 4,6-dehydratase protein_id CAB05932.1 - *Streptococcus pneumoniae* |
| An17g00560 | strong similarity to peripheral membrane protein required for vacuolar protein sorting Vps17 - *Saccharomyces cerevisiae* |
| An17g01100 | similarity to hypothetical protein SPAC637.04 - *Schizosaccharomyces pombe* |
| An17g01170 | strong similarity to hypothetical membrane protein YOR154w - *Saccharomyces cerevisiae* |
| An17g01420 | strong similarity to hypothetical transport protein SPCC830.08c - *Schizosaccharomyces pombe* |
| An17g01440 | strong similarity to peroxisomal membrane protein PEX3 - *Pichia angusta* |
| An17g01550 | strong similarity to Na+-H+ antiporter Nha2 - *Saccharomyces cerevisiae* |
| An17g01560 | strong similarity to sucrose transport protein SUT1 - *Oryza sativa* |
| An17g01955 | similarity to rhoGAP homolog DdRacGAP - *Dictyostelium discoideum* |
| An17g01965 | similarity to heptaprenyl diphosphate synthetase ORFII from patent EP699761-A2 - *Bacillus stearothermophilus* |
| An17g01965 | similarity to heptaprenyl diphosphate synthetase ORFII from patent EP699761-A2 - *Bacillus stearothermophilus* |
| An17g02030 | strong similarity to 2-acyltransferase from patent WO9413814-A - *Zea mays* |
| An17g02180 | similarity to hypothetical eIF-3 p110 subunit - *Caenorhabditis elegans* |
| An17g02290 | strong similarity to myosin Myo2 - *Saccharomyces cerevisiae* |
| An17g02340 | strong similarity to cytosolic serine--tRNA ligase Ses1 - *Saccharomyces cerevisiae* |
| An18g00100 | similarity to hypothetical protein PA1213 - *Pseudomonas aeruginosa* |
| An18g00740 | hypothetical protein |
| An18g01290 | strong similarity to hypothetical protein encoded by An13g01340 - *Aspergillus niger* |
| An18g01320 | strong similarity to extracellular protease precursor Bar1 - *Saccharomyces cerevisiae* |
| An18g01690 | strong similarity to hypothetical protein YOR155c - *Saccharomyces cerevisiae* |
| An18g01840 | similarity to zinc-cluster transcription factor fcr1 - *Candida albicans* |
| An18g02020 | disulfide isomerase tigA - *Aspergillus niger* |
| An18g02150 | similarity to hypothetical protein CAF05965.1 - *Neurospora crassa* |
| An18g02170 | strong similarity to mannosyltransferase Ktr5 - *Saccharomyces cerevisiae* |
| An18g02340 | strong similarity to nitrate assimilation regulatory protein nirA - *Aspergillus nidulans* [truncated ORF] |
| An18g02360 | strong similarity to Dol-P-Man dependent alpha(1-3) mannosyltransferase Alg3 - *Saccharomyces cerevisiae* |
| An18g02490 | strong similarity to ARF guanine-nucleotide exchange factor 2 Gea2 - *Saccharomyces cerevisiae* |
| An18g02600 | hypothetical protein |
| An18g02760 | similarity to hypothetical protein T21J18.40 - *Arabidopsis thaliana* |
| An18g02770 | weak similarity to hypothetical protein encoded by An07g09090 - *Aspergillus niger* |
| An18g03070 | strong similarity to ORF2319 polynucleotide sequence SEQ ID NO:4637 from patent WO200058473-A2 - *Homo sapiens* |
| An18g03200 | strong similarity to actin like protein act2p - *Schizosaccharomyces pombe* |
| An18g03280 | similarity to hypothetical protein B13D24.040 - *Neurospora crassa* |
| An18g03490 | similarity to cation-dependent mannose 6-phosphate receptor precursor CD-MPR - *Bos taurus* |
| An18g03580 | similarity to hypothetical protein SPBC543.08 - *Schizosaccharomyces pombe* |
| An18g03660 | similarity to ser/thr protein kinase Prk1 - *Saccharomyces cerevisiae* |
| An18g03920 | strong similarity to defender against apoptotic cell death DAD1 - *Homo sapiens* |
| An18g04240 | strong similarity to Snf1 interacting protein Sip3 - *Saccharomyces cerevisiae* |
| An18g04260 | similarity to secreted protein HNTME13 from patent WO9839446-A2 - *Homo sapiens* |
| An18g04670 | similarity to UDP-N-acetylglucosamine transporter SLC35A3 - *Homo sapiens* |
| An18g05220 | strong similarity to gamma-butyrobetaine hydroxylase - *Pseudomonas sp.* |
| An18g05470 | similarity to C-3 sterol dehydrogenase Erg26 - *Saccharomyces cerevisiae* |
| An18g05850 | strong similarity to hypothetical protein SPAC30C2.08 - *Schizosaccharomyces pombe* |
| An18g05890 | similarity to pyruvate dehydrogenase phosphatase isoenzyme 1 PDP1 - *Rattus norvegicus* |
| An18g05910 | strong similarity to hypothetical glycosyl transferase SPCC330.08 - *Schizosaccharomyces pombe* |
| An18g06120 | strong similarity to secreted protein vc33_1 from patent WO200011015-A1 - *Homo sapiens* |
| An18g06220 | strong similarity to alpha-mannosidase Mns1 - *Saccharomyces cerevisiae* |
| An18g06290 | strong similarity to calcium P-type ATPase nca-1 - *Neurospora crassa* |
| An18g06430 | weak similarity to chorion gene s18 - Ceratitis capitata |
| An18g06470 | strong similarity to DnaJ-like protein MTJ1 - *Mus musculus* |
| An18g06740 | strong similarity to hypothetical protein YHR181w - *Saccharomyces cerevisiae* |
| An19g00320 | strong similarity to vacuolar H(+)/Ca(2+) exchanger Vcx1 - *Saccharomyces cerevisiae* |
| An19g00330 | strong similarity to vacuolar H(+)/Ca(2+) exchanger Vcx1 - *Saccharomyces cerevisiae* |
| An19g00340 | strong similarity to vacuolar H(+)/Ca(2+) exchanger Vcx1 - *Saccharomyces cerevisiae* |
| An19g00360 | similarity to hypothetical protein encoded by An08g05310 - *Aspergillus niger* |
