# Supplementary material for: Genome-wide expression analysis upon constitutive activation of the HacA bZIP transcription factor in Aspergillus niger reveals a coordinated cellular response to counteract ER stress
Source: BMC Genomics. 2012 Jul 30;13:350. doi: 10.1186/1471-2164-13-350 (PMC3472299; doi:10.1186/1471-2164-13-350)
Supplement: Additional file 5 — HacACA up-regulated genes that contain at least one UPRE sequence. Subset of all differentially expressed genes (Additional file 3). [file 1471-2164-13-350-S5.doc]

Additional file 5: List of HacA^CA^ up-regulated genes that contain at least one UPRE sequence within a region of -400 bp from the start codon.

| **Gene ID** | **Gene name** | **Description** | **Fold change** | | |
| --- | --- | --- | --- | --- | --- |
|  |  |  | **HacA^CA-1^/ HacA^WT^** | **HacA^CA-2^/ HacA^WT^** | **HacA^CA-3^/ HacA^WT^** |
| **Protein folding** | | | | | |
| An01g04600 | *prpA* | PDI related protein A – *A. niger* | **4.6** | **4.3** | **4.3** |
| An02g14800 | *pdiA* | protein disulfide isomerase – *A. niger* | **2.8** | **3.1** | **3.1** |
| An04g02020 | *cypB* | strong similarity to cyclophilin – *A. nidulans* | **2.9** | **3.5** | **3.5** |
| An11g04180 | *bipA* | dnaK-type molecular chaperone – *A. niger* | **4.6** | **4.5** | **4.7** |
| An18g02020 | *tigA* | disulfide isomerase – *A. niger* | **3.2** | **3.2** | **3.3** |
| An16g07620 | *eroA* | strong similarity to endoplasmatic reticulum oxidising protein Ero1 – *S. cerevisiae* | **4.9** | **5.7** | **6.2** |
| **Lipid metabolism** | | | | | |
| An02g04530 |  | strong similarity to cyclopropane-fatty-acyl-phospholipid synthase - E*. coli* | **2.2** | **1.9** | **1.9** |
| An02g05150 |  | strong similarity to C-8,7 sterol isomerase – *A. thaliana* | **1.8** | **1.9** | **1.9** |
| An02g11290 |  | similarity to phosphoinositide-specific phospholipase C lc1p – *S. pombe* | 1.2* | 1.6* | **1.7** |
| An02g13410 |  | similar to acetyl-coenzyme A transporter AT-1 – *H. sapiens* | **6.1** | **6.4** | **6.6** |
| An14g00270 |  | weak similarity to dolichol-phosphate-mannose synthase DPM3 - *H. sapiens* | **1.7** | **2.1** | **2.1** |
| **Transport (related) within the cell** | | | | | |
| An01g04320 | *ERV46* | ERV46 (YAL042w) – involved in COPII vesicle fusion– *S. cerevisiae* | **3.9** | **4.8** | **4.6** |
| An02g01510 | *SEC62* | strong similarity to component of the ER protein translocation machinery Sec62 – *S. cerevisiae* | **1.9** | **2.4** | **2.5** |
| An02g01690 | *SEC31* | strong similarity to the p150 component of the COPII coat of secretory pathway vesicles Sec31 – *S. cerevisiae* | **2.6** | **2.7** | **2.6** |
| An07g02170 | *BOS1* | similarity to transport protein Bos1 – *S. cerevisiae* |  |  |  |
| An07g06030 |  | strong similarity to coatomer gamma subunit 2 copg2 – *H. sapiens* | **2.3** | **2.0** | **2.0** |
| An08g05570 | *SEC5* | similarity to secretory protein Sec5 – *S. cerevisiae* | **1.6** | **1.9** | **2.0** |
| **Glycosylation** | | | | | |
| An03g04410 | *ALG5* | similar to glucosyltransferase ALG5 – *S. cerevisiae* | **4.1** | **4.5** | **4.7** |
| An07g04190 | *WBP1* | strong similarity to subunit of oligosacharyltransferase complex– *S. cerevisiae* | **3.4** | **3.7** | **3.8** |
| **ERAD** | | | | | |
| An03g04340 |  | strong similarity to ER membrane translocation facilitator Sec61 – *Y. lipolytica* | **2.6** | **2.6** | **2.6** |
| An04g06990 |  | similarity to alpha 1,2-mannosidase IB – *H. sapiens* | **2.0** | **2.9** | **3.5** |
| An15g00640 | *derA* | strong similarity to hypothetical protein GABA-A receptor epsilon subunit – *C. elegans* | **4.0** | **6.0** | **6.4** |
| **Other** | | | | | |
| An01g02280 |  | hypothetical protein | **2.0** | **2.1** | **2.2** |
| An01g08420 |  | strong similarity to calcium-binding protein precursor cnx1p – *S. pombe* | **2.8** | **3.0** | **3.1** |
| An02g02260 | *YMR155w* | strong similarity to hypothetical membrane protein YMR155w – *S. cerevisiae* | **3.3** | **4.0** | **4.0** |
| An02g12050 | *VMA21* | similarity to V-ATPase assembly protein Vma21 – *S. cerevisiae* | **2.4** | **3.0** | **3.0** |
| An02g14940 |  | strong similarity to human transmembrane protein HTMPN-23 – *H. sapiens* | **3.6** | **4.2** | **4.5** |
| An04g02050 |  | similarity to hypoxia-induced protein – *H. sapiens* | **1.6** | **1.6** | **1.7** |
| An03g04720 |  | strong similarity to serine threonine protein kinase SNF1 – *C. carbonum* | **1.5** | **1.5** | **1.6** |
| An04g04080 |  | similarity to hypothetical negative acting factor related protein – *N. crassa* | **1.8** | **2.3** | **2.5** |
| An04g06310 |  | strong similarity to hypothetical protein CAB91735.2 – *N. crassa* | **1.9** | **1.8** | **1.8** |
| An04g07430 |  | similarity to hypothetical protein encoded by An07g09430 – *A. niger* | **2.4** | **4.0** | **4.4** |
| An05g01890 |  | strong similarity to hypothetical protein related to host-specific AK-toxin Akt2 B23L21.350 – *N. crassa* | **1.5** | **1.6** | **1.9** |
| An05g02360 |  | strong similarity to hypothetical protein encoded by An18g01530 – *A. niger* | **1.8** | **2.1** | **2.3** |
| An07g03880 | *pepC* | serine proteinase *pepC* – *A. niger* | **1.6** | **2.0** | **2.1** |
| An07g09170 |  | strong similarity to breast cancer resistance protein 1 BCRP1 – *M. musculus* | **1.5** | **1.7** | **1.7** |
| An08g04120 |  | similarity to hypothetical mold-specific protein MS8 – *A. capsulatus* | **1.8** | **2.3** | **2.4** |
| An11g07530 |  | similarity to protein Notchless – *D. melanogaster* | **2.7** | **2.7** | **2.9** |
| An12g02450 |  | strong similarity to alpha-glucan synthase mok1p - *Schizosaccharomyces pombe* | **1.8** | **1.7** | **1.8** |
| An12g08700 |  | hypothetical protein | **2.1** | **2.9** | **3.1** |
| An14g00190 |  | weak similarity to hypothetical protein At2g17590 – *A. thaliana* | **1.5** | **1.9** | **2.0** |
| An16g09150 |  | strong similarity to hypothetical protein BM-021 – *H. sapiens* | **2.1** | **2.4** | **2.8** |
| An17g02340 | *SES1* | strong similarity to cytosolic serine--tRNA ligase Ses1 – *S. cerevisiae* | **1.5** | **1.5** | **1.5** |
| An18g03660 | *PRK1* | similarity to ser/thr protein kinase Prk1 – *S. cerevisiae* | **1.4** | **1.4** | **1.6** |
| An18g03920 |  | strong similarity to defender against apoptotic cell death DAD1 – *H. sapiens* | **2.8** | **3.4** | **3.7** |
| An18g04260 |  | similarity to secreted protein HNTME13 – *H. sapiens* | **6.8** | **8.0** | **8.5** |
| An18g06290 |  | strong similarity to calcium P-type ATPase nca-1 – *N. crassa* | **2.1** | **1.9** | **2.0** |

*Not significantly differentially expressed
