# Supplementary material for: Genome-wide expression analysis upon constitutive activation of the HacA bZIP transcription factor in Aspergillus niger reveals a coordinated cellular response to counteract ER stress
Source: BMC Genomics. 2012 Jul 30;13:350. doi: 10.1186/1471-2164-13-350 (PMC3472299; doi:10.1186/1471-2164-13-350)
Supplement: Additional file 8 — GO analysis of biological processes enriched in the up-regulated set of genes in HacACA. Subset of all differentially expressed genes (Additional file 3). [file 1471-2164-13-350-S8.doc]

Additional file 8: GO analysis of biological processes enriched in the up-regulated set of genes in HacA^CA^.

| **GO term** | **Description** | **FDR** | **# genes enriched** | **# all genes** | **Genes enriched** |
| --- | --- | --- | --- | --- | --- |
| GO:0006890 | retrograde vesicle-mediated transport, Golgi to ER | 1.89E-05 | 9 | 17 | An02g07090,An02g05870,An12g04830,An01g14250,An15g01510,An07g06030,An16g02460,An16g05370,An09g04170, |
| GO:0031204 | posttranslational protein targeting to membrane, translocation | 2.01E-05 | 6 | 7 | An02g01510,An03g04340,An01g11630,An16g08830,An01g13070,An11g04180, |
| GO:0006490 | oligosaccharide-lipid intermediate assembly | 2.01E-05 | 6 | 7 | An02g12630,An18g05910,An04g08820,An14g05910,An18g02360,An06g01100, |
| GO:0090114 | COPII-coated vesicle budding | 6.73E-05 | 6 | 8 | An02g01690,An15g01520,An16g03320,An04g00360,An01g04040,An08g10650, |
| GO:0007035 | vacuolar acidification | 1.56E-03 | 4 | 5 | An04g05310,An02g02020,An17g01550,An08g06750, |
| GO:0006493 | protein amino acid O-linked glycosylation | 1.56E-03 | 4 | 5 | An07g10350,An16g08490,An11g09890,An14g03910, |
| GO:0030433 | ER-associated protein catabolic process | 3.67E-03 | 7 | 20 | An08g09000,An18g06220,An05g00880,An03g06880,An02g05890,An04g01720,An11g04180, |
| GO:0006616 | SRP-dependent cotranslational protein targeting to membrane, translocation | 3.75E-03 | 4 | 6 | An03g04340,An01g11630,An16g08830,An11g04180, |
| GO:0006986 | response to unfolded protein | 7.34E-03 | 6 | 17 | An05g00880,An03g02770,An11g04180,An01g13220,An08g01480,An01g06550, |
| GO:0006897 | endocytosis | 9.90E-03 | 13 | 70 | An18g03660,An07g08830,An07g10420,An12g00120,An17g02290,An16g08470,An01g07330,An15g01510,An02g03460,An05g00200,An15g04490,An04g07040,An02g07780, |
| GO:0015786 | UDP-glucose transport | 1.19E-02 | 3 | 4 | An03g06940,An11g02020,An18g04260, |
| GO:0048199 | vesicle targeting, to, from or within Golgi | 2.39E-02 | 3 | 5 | An15g01520,An01g04040,An16g05370, |
| GO:0006751 | glutathione catabolic process | 2.39E-02 | 3 | 5 | An08g04260,An11g11180,An13g01300, |
| GO:0006901 | vesicle coating | 2.39E-02 | 3 | 5 | An15g01520,An01g04040,An16g05370, |
| GO:0006465 | signal peptide processing | 2.39E-02 | 3 | 5 | An01g00560,An09g05420,An16g07390, |
| GO:0030384 | phosphoinositide metabolic process | 2.54E-02 | 7 | 29 | An13g00110,An01g14140,An09g01240,An14g00900,An11g06770,An01g12990,An04g02480, |
| GO:0006895 | Golgi to endosome transport | 3.91E-02 | 4 | 11 | An01g07330,An08g01410,An07g08220,An11g04750, |
| GO:0051345 | positive regulation of hydrolase activity | 4.11E-02 | 3 | 6 | An04g00360,An08g10570,An01g06550, |
| GO:0008610 | lipid biosynthetic process | 4.18E-02 | 15 | 104 | An08g00560,An15g00630,An14g03360,An15g01460,An04g00600,An02g09910,An07g09690,An11g06770,An09g00620,An01g07640,An01g14140,An14g00900,An01g12990,An13g00040,An04g05250, |
