# Supplementary material for: Genome-wide expression analysis upon constitutive activation of the HacA bZIP transcription factor in Aspergillus niger reveals a coordinated cellular response to counteract ER stress
Source: BMC Genomics. 2012 Jul 30;13:350. doi: 10.1186/1471-2164-13-350 (PMC3472299; doi:10.1186/1471-2164-13-350)
Supplement: Additional file 9 — GO analysis of biological processes enriched in the down-regulated set of genes in HacACA. Subset of all differentially expressed genes (Additional file 3). [file 1471-2164-13-350-S9.doc]

Additional file 9: GO analysis of biological processes enriched in the down-regulated set of genes in HacA^CA^.

| **GO term** | **Description** | **FDR** | **# genes enriched** | **# all genes** | **Genes enriched** |
| --- | --- | --- | --- | --- | --- |
| GO:0006544 | glycine metabolic process | 6.39E-03 | 4 | 8 | An08g03070,An08g04390,An15g03260,An14g01150, |
| GO:0008645 | hexose transport | 6.39E-03 | 4 | 8 | An02g14380,An02g03540,An11g01100,An16g04160, |
| GO:0006012 | galactose metabolic process | 6.39E-03 | 6 | 20 | An09g00260,An11g01120,An02g03590,An16g04160,An11g10890,An02g11320, |
| GO:0006096 | glycolysis | 7.63E-03 | 6 | 21 | An04g02090,An07g04300,An08g02260,An11g02550,An02g14380,An16g05420, |
| GO:0006979 | response to oxidative stress | 1.56E-02 | 14 | 110 | An18g01170,An02g07930,An07g03980,An06g01660,An04g04870,An16g06100,An02g05830,An08g02310,An02g11320,An16g00920,An11g10890,An02g12140,An11g02040,An15g03220, |
| GO:0005982 | starch metabolic process | 1.70E-02 | 4 | 11 | An04g06920,An01g10930,An11g03340,An04g06910, |
| GO:0046185 | aldehyde catabolic process | 1.70E-02 | 4 | 11 | An01g07030,An08g07290,An10g00510,An17g01530, |
| GO:0006071 | glycerol metabolic process | 2.19E-02 | 5 | 19 | An11g01120,An10g00510,An17g01530,An11g03110,An01g06970, |
| GO:0019679 | propionate metabolic process, methylcitrate cycle | 2.25E-02 | 3 | 6 | An04g05620,An16g07110,An15g01920, |
| GO:0006094 | gluconeogenesis | 2.30E-02 | 6 | 28 | An04g02090,An07g04300,An08g02260,An11g02550,An02g14380,An16g05420, |
| GO:0005984 | disaccharide metabolic process | 2.45E-02 | 5 | 20 | An04g06920,An01g10930,An01g01540,An11g10990,An01g10350, |
| GO:0006083 | acetate metabolic process | 2.45E-02 | 5 | 20 | An04g05620,An11g02550,An12g04640,An16g07110,An08g07290, |
| GO:0006740 | NADPH regeneration | 2.86E-02 | 4 | 13 | An16g05420,An11g02040,An02g12140,An08g07290, |
| GO:0019321 | pentose metabolic process | 3.17E-02 | 7 | 40 | An12g00030,An07g02210,An15g05450,An01g06970,An02g12140,An04g05860,An11g02040, |
| GO:0006567 | threonine catabolic process | 3.23E-02 | 3 | 7 | An08g07290,An15g03260,An17g01530, |
| GO:0006099 | tricarboxylic acid cycle | 3.50E-02 | 5 | 22 | An14g04400,An07g02160,An08g10530,An12g07850,An15g01920, |
| GO:0015812 | gamma-aminobutyric acid transport | 4.47E-02 | 4 | 15 | An02g09540,An16g02000,An14g01850,An03g00430, |
| GO:0033609 | oxalate metabolic process | 4.51E-02 | 3 | 8 | An07g08390,An10g00510,An17g01530, |
