# Supplementary material for: Genome-wide expression analysis upon constitutive activation of the HacA bZIP transcription factor in Aspergillus niger reveals a coordinated cellular response to counteract ER stress
Source: BMC Genomics. 2012 Jul 30;13:350. doi: 10.1186/1471-2164-13-350 (PMC3472299; doi:10.1186/1471-2164-13-350)
Supplement: Additional file 10 — Expression values of selected genes related to enriched GO terms of ER associated processes. Subset of all differentially expressed genes (Additional file 3). [file 1471-2164-13-350-S10.doc]

Additional file 10: Expression values of selected genes related to enriched GO terms of ER associated processes.

| **Gene ID** | **Gene name: *A. niger* or *S. cerevisiae*** | **Description** | **Fold change** | | | | | | **GO-term** |
| --- | --- | --- | --- | --- | --- | --- | --- | --- | --- |
|  |  |  | **HacA^CA-1^/**  **HacA^WT^** | **HacA^CA-2^/**  **HacA^WT^** | **HacA^CA-3^/**  **HacA^WT^** | **HacA^CA-2^/**  **HacA^CA-1^** | **HacA^CA-2^/**  **HacA^CA-3^** | **HacA^CA-3^/**  **HacA^CA-1^** | **Biological Process** |
| **Signal recognition particle** | | | | | | | | | |
| An01g02800 |  | strong similarity to signal recognition particle 68K protein SRP68 – *C. lupus* | 1.4 | 1.3 | 1.3 | -1.1 | 1.0 | -1.1 | * |
| An04g06890 |  | similarity to 72-kD protein of the signal recognition particle SRP72 – *C. lupus* | 1.5 | 1.2 | 1.2 | -1.2 | 1.0 | -1.1 | * |
| An01g10070 | *SEC65* | strong similarity to signal recognition particle chain Sec65 – *S. cerevisiae* | **1.9** | **1.9** | **1.8** | 1.0 | 1.0 | 1.0 | * |
| An15g06470 |  | similarity to signal sequence receptor alpha chain – *C. lupus familiaris* | **1.6** | **1.5** | 1.5 | -1.1 | -1.1 | -1.1 | * |
| An07g05800 |  | similarity to signal recognition particle protein srp14 | 1.6 | 1.7 | 1.7 | 1.1 | 1.0 | 1.1 | * |
| An09g06320 | *SRP54* | similarity to signal recognition particle 54K protein SRP54 – *S. cerevisiae* | 1.2 | 1.2 | 1.1 | -1.1 | 1.0 | -1.1 | * |
| **Signal peptidase cleavage** | | | | | | | | | |
| An01g00560 | *SEC11* | strong similarity to signal peptidase subunit Sec11 – *S. cerevisiae* | **3.4** | **4.5** | **4.7** | 1.3 | 1.1 | 1.4 | GO:0006465 |
| An16g07390 | *SPC2* | strong similarity to signal peptidase subunit Spc2 – *S. cerevisiae* | **3.2** | **4.0** | **4.6** | 1.2 | 1.2 | 1.5 | GO:0006465 |
| An09g05420 |  | similarity to signal peptidase subunit Spc3 – *S. cerevisiae* | **4.7** | **5.4** | **5.9** | 1.1 | 1.1 | 1.2 | GO:0006465 |
| **SRP dependent translocation** | | | | | | | | | |
| An03g04340 |  | strong similarity to ER membrane translocation facilitator Sec61 – *Y. lipolytica* | **2.6** | **2.6** | **2.6** | 1.0 | 1.0 | 1.0 | GO:0031204  GO:0006465 |
| An01g03820 | *SBH2* | strong similarity to ER protein-translocation complex subunit Sbh2 – *S. cerevisiae* | 1.9 | **2.3** | **2.3** | 1.2 | 1.0 | 1.2 | * |
| An01g11630 | *SSS1* | strong similarity to translocation complex component Sss1 – *S. cerevisiae* | **2.6** | **2.9** | **3.0** | 1.1 | 1.0 | 1.1 | GO:0031204  GO:0006465 |
| An02g01510 | *SEC62* | strong similarity to component of the ER protein translocation machinery Sec62 – *S. cerevisiae* | **1.9** | **2.4** | **2.5** | 1.2 | 1.0 | 1.3 | GO:0031204 |
| An01g13070 | *SEC63* | strong similarity to signal recognition particle receptor Sec63 – *S. cerevisiae* | **3.9** | **5.0** | **5.3** | 1.3 | 1.1 | 1.3 | GO:0031204 |
| An16g08830 | *SEC71* | strong similarity to component of ER protein-translocation  subcomplex Sec71 - *S. cerevisiae* | **2.7** | **3.0** | **3.1** | 1.1 | 1.0 | 1.2 | GO:0031204  GO:0006465 |
| An15g01670 |  | strong similarity to signal sequence receptor alpha subunit  SRP101 – *Y. lipolytica* | 1.2 | 1.4 | 1.4 | 1.2 | 1.0 | 1.2 | * |
| **Protein folding ER** | | | | | | | | | |
| An04g02020 | *cypB* | strong similarity to cyclophilin cypB – *A. nidulans* | **2.9** | **3.5** | **3.5** | **1.2** | 1.0 | **1.2** | * |
| An11g04180 | *bipA* | dnaK-type molecular chaperone bipA – *A. niger* | **4.6** | **4.5** | **4.7** | 1.0 | 1.1 | 1.0 | GO:0031204  GO:0030433  GO:0006465  GO:0006986 |
| An02g14800 | *pdiA* | protein disulfide isomerase A pdiA – *A. niger* | **2.8** | **3.1** | **3.1** | 1.1 | 1.0 | **1.1** | * |
| An18g02020 | *tigA* | disulfide isomerase tigA – *A. niger* | **3.2** | **3.2** | **3.3** | 1.0 | 1.0 | 1.0 | * |
| An01g04600 | *prpA* | PDI related protein A prpA – *A. niger* | **4.6** | **4.3** | **4.3** | -1.1 | 1.0 | -1.1 | * |
| An01g00160 | *hacA* | Hac1 b-zip transcription factor | 1.1 | 1.4 | 1.4 | 1.3 | 1.0 | 1.3 | * |
| An02g05890 | *epsA* | strong similarity to protein disulfide-isomerase pdi1 - *C. elegans* | **1.7** | **1.7** | **1.7** | 1.0 | 1.0 | 1.0 | GO:0030433 |
| An16g07620 | *eroA* | strong similarity to endoplasmatic reticulum oxidising protein Ero1 – *S. cerevisiae* | **4.9** | **5.7** | **6.2** | 1.2 | 1.1 | **1.3** | * |
| An18g04260 |  | UDP-Glc/Gal endoplasmic reticulum nucleotide sugar transporter | **6.8** | **8.0** | **8.5** | **1.2** | 1.1 | **1.2** | * |
| An08g07810 |  | similarity to flavin adenine dinucleotide synthase Fad1 – *S. cerevisiae* | **1.4** | 1.0 | 1.1 | **-1.2** | 1.1 | **-1.2** | * |
| An08g06370 |  | strong similarity to GTP-cyclohydrolase II RIB1 – *P. guilliermondii* | 1.1 | 1.1 | 1.1 | 1.0 | 1.0 | 1.0 | * |
| An12g06490 |  | similarity to N-oxide-forming dimethylaniline monooxygenase FMO1 – *H. sapiens* | 1.1 | 1.0 | 1.0 | 0.9 | 1.0 | -1.1 | * |
| An18g06470 |  | strong similarity to DnaJ-like protein MTJ1 – *M. musculus* | **2.8** | **2.9** | **3.1** | 1.0 | 1.1 | 1.1 | * |
| An05g00880 | *SCJ1* | strong similarity to dnaJ protein homolog Scj1 – Saccharomyces cerevisiae | **5.1** | **4.9** | **5.1** | 1.0 | 1.0 | 1.0 | GO:0030433  GO:0006986 |
| An01g06670 |  | CypB strong similarity to peptidyl-prolyl isomerase FKBP-21 – *N. crassa* | **1.9** | **1.9** | **1.8** | 1.0 | 1.0 | 1.0 |  |
| An01g13220 | *LhsA* | strong similarity to 150 kDa oxygen regulated protein ORP150 –*R. norvegicus* | **7.8** | **7.8** | **8.1** | 1.0 | 1.0 | 1.0 | GO:0006986 |
| **ER Quality Control** | | | | | | | | | |
| An15g01420 | *CWH41* | similar to glucosidase I CWH41 – *S. cerevisiae* | **4.2** | **4.2** | **4.3** | 1.0 | 1.0 | 1.0 | * |
| An09g05880 | *ROT2* | similar to alpha-glucosidase II ROT2 - *S. cerevisiae* | **3.4** | **2.9** | **3.1** | -1.1 | 1.0 | -1.1 | * |
| An13g00620 |  | similar to beta subunit of an ER alpha-glucosidase – *S. cerevisiae* | **3.2** | **3.1** | **3.4** | 1.0 | 1.1 | 1.0 | * |
| An07g06430 | *KRE5* | similar to glucosyltransferase KRE5 – *S. cerevisiae* | **4.5** | **4.0** | **4.0** | -1.1 | 1.0 | -1.1 | * |
| An18g06220 | *MNS1* | similar to alpha-mannosidase MNS1 – *S. cerevisiae* | **4.2** | **4.7** | **5.0** | 1.0 | 1.1 | 1.1 | GO:0030433 |
| An04g06990 |  | strong similarity to alpha 1,2-mannosidase IC – *H.sapiens* | **2.0** | **2.9** | **3.5** | 1.2 | 1.2 | **1.8** | * |
| An12g00340 | *HTM1* | similar to mannosidase HTM1 – *S. cerevisiae* | **3.2** | **2.9** | **3.1** | -1.1 | 1.1 | 1.0 | * |
| **ER-associated protein catabolic process** | | | | | | | | | |
| An08g09000 |  | strong similarity to ubiquitin like protein Dsk2 | **1.8** | **1.7** | **1.9** | 1.0 | 1.1 | 1.1 | GO:0030433 |
| An03g06880 | *PEP1* | strong similarity to carboxypeptidase Y-sorting protein | **2.4** | **2.4** | **2.6** | 1.0 | 1.1 | 1.1 | GO:0030433 |
| An04g01720 |  | similarity to DnaJ protein SIS1 -Cryptococcus curvatus; | **1.8** | **2.3** | **2.2** | 1.2 | 1.0 | 1.2 | GO:0030433 |
| **Unfolded Protein Response** | | | | | | | | | |
| An03g02770 |  | phosphoesterase involved in downregulation of the UPR | **1.4** | **1.4** | **1.6** | 1.0 | 1.1 | 1.1 | GO:0006986 |
| An08g01480 | *TRL1* | strong similarity to tRNA ligase Trl1 –S. cerevisiae | **1.7** | **2.1** | **2.2** | 1.2 | 1.1 | 1.3 | GO:0006986 |
| An01g06550 | *IRE1* | strong similarity to protein kinase Ire1 –S. cerevisiae | **2.0** | **2.1** | **2.1** | 1.0 | 1.0 | 1.0 | GO:0006986  GO:0051345 |

* Not present in GO-list; GO:0006465: signal peptide processing; GO:0031204: posttranslational protein targeting to membrane translocation; GO:0030433: ER-associated protein catabolic process; GO:0006986: response to unfolded protein; GO:0051345: positive regulation of hydrolase activity. Values in bold represent a significant fold change with a FDR<0.005.
