# Supplementary material for: Genome-wide expression analysis upon constitutive activation of the HacA bZIP transcription factor in Aspergillus niger reveals a coordinated cellular response to counteract ER stress
Source: BMC Genomics. 2012 Jul 30;13:350. doi: 10.1186/1471-2164-13-350 (PMC3472299; doi:10.1186/1471-2164-13-350)
Supplement: Additional file 11 — Expression values of selected genes related to enriched GO terms associated with vesicle transport within the cell. Subset of all differentially expressed genes (Additional file 3). [file 1471-2164-13-350-S11.doc]

Additional file 11: Expression values of selected genes related to enriched GO terms associated with vesicle transport within the cell.

| **Gene ID** | **Gene name: *A. niger* or *S. cerevisiae*** | **Description** | **Fold change** | | | | | | **GO-term** |
| --- | --- | --- | --- | --- | --- | --- | --- | --- | --- |
|  |  |  | **HacA^CA-1^/**  **HacA^WT^** | **HacA^CA-2^/**  **HacA^WT^** | **HacA^CA-3^/**  **HacA^WT^** | **HacA^CA-2^/**  **HacA^CA-1^** | **HacA^CA-2^/**  **HacA^CA-3^** | **HacA^CA-3^/**  **HacA^CA-1^** | **Biological Process** |
| **Golgi to ER retrograde transport (COPI)** | | | | | | | | | |
| An02g07090 |  | strong similarity to ASNA1 product arsenite translocating ATPase - *Homo sapiens* | **1.6** | **1.7** | **1.6** | 1.0 | 1.0 | 1.0 | GO:0006890 |
| An12g04830 | *RET3* | strong similarity to coatomer protein zeta chain Ret3 - *Saccharomyces cerevisiae* | **1.8** | **2.3** | **2.3** | 1.2 | 1.0 | 1.2 | GO:0006890 |
| An02g05870 |  | strong similarity to coatomer beta subunit copB2 - *Homo sapiens* [putative frameshift] | **1.9** | **1.5** | **1.5** | -1.2 | 1.0 | -1.2 | GO:0006890 |
| An01g14250 |  | strong similarity to delta subunit of the coatomer delta-coat protein CopD - *Bos taurus* | **1.8** | **2.0** | **2.0** | 1.1 | 1.0 | 1.1 | GO:0006890 |
| An15g01510 | *DRS2* | strong similarity to P-type ATPase Drs2 - *Saccharomyces cerevisiae* | **2.1** | **1.8** | **1.9** | -1.1 | 1.0 | -1.1 | GO:0006890 |
| An07g06030 |  | strong similarity to coatomer gamma subunit 2 copg2 - *Homo sapiens* | **2.3** | **2.0** | **2.0** | -1.1 | 1.0 | -1.1 | GO:0006890 |
| An16g05370 | *GLO3* | similarity to zinc-finger protein Glo3 - *Saccharomyces cerevisiae* | **2.3** | **2.4** | **2.5** | 1.0 | 1.0 | 1.1 | GO:0006890  GO:0006901  GO:0048199 |
| An09g04170 | *SLY1* | strong similarity to protein Sly1 - *Saccharomyces cerevisiae* | **2.6** | **3.0** | **3.0** | 1.2 | 1.0 | 1.2 | GO:0006890 |
| An08g01250 |  | weak similarity to COPI-interacting protein 7 CIP7 - *Arabidopsis thaliana* | **2.2** | **1.9** | **2.0** | -1.1 | 1.0 | -1.1 | ***** |
| An08g06330 |  | strong similarity to epsilon-COP - *Cricetulus griseus* | **2.0** | **2.1** | **2.2** | 1.1 | 1.0 | 1.1 | * |
| An08g03690 | *SRF2* | strong similarity to ADP-ribosylation factor. GTPase of the Rassuperfamily. Arf2p - *Saccharomyces cerevisae* | 1.2 | 1.4 | **1.4** | 1.1 | 1.1 | 1.2 | ***** |
| **COPII ER to Golgi** | | | | | | | | | |
| An02g01690 | *SEC31* | strong similarity to the p150 component of the COPII coat of secretory pathway vesicles Sec31 - *Saccharomyces cerevisiae* | **2.6** | **2.7** | **2.6** | 1.0 | 1.0 | 1.0 | GO:0090114 |
| An15g01520 | *SEC16* | vesicle coat protein Sec16 - *Saccharomyces cerevisiae* | **2.6** | **2.4** | **2.4** | -1.1 | 1.0 | -1.1 | GO:0090114  GO:0006901  GO:0048199 |
| An16g03320 | *SEC24* | strong similarity to transport protein Sec24A - *Saccharomyces cerevisiae* | **3.0** | **2.5** | **2.6** | -1.2 | 1.0 | -1.1 | GO:0090114 |
| An04g00360 | *SEC13* | strong similarity to transport vesicle formation protein Sec13p - *Saccharomyces cerevisiae* | **2.1** | **2.1** | **2.1** | 1.0 | 1.0 | 1.0 | GO:0090114 |
| An01g04040 | *SAR1p* | SARA strong similarity to GTPase. GTP-binding protein of the ARF family - *Saccharomyces cerevisae* | **1.4** | **1.5** | **1.5** | 1.1 | 1.0 | 1.1 | GO:0090114  GO:0006901  GO:0048199 |
| An08g10650 | *SEC24* | strong similarity to transport protein Sec24p - *Saccharomyces cerevisiae* | **2.7** | **2.2** | **2.3** | -1.2 | 1.0 | -1.1 | GO:0090114 |
| An08g03270 | *SEC26* | strong similarity to beta-COP Sec26p - *Saccharomyces cerevisiae* | **1.9** | **2.1** | **2.0** | 1.1 | 1.0 | 1.1 | * |
| An01g04730 | *SEC23* | SEC23 (YPR181c) – COPII complex SU GTPase-activating protein | **1.8** | 1.7 | **1.7** | -1.1 | 1.1 | 1.0 | * |
| An08g03590 | *EMP24* | EMP24 (YGL200c) – COPII vesicle membrane component | **2.7** | **3.1** | **3.1** | 1.2 | 1.0 | 1.2 | * |
| An04g01780 | *ERP1* | ERP1 (YAR002c-a) – COPII vesicle component | **2.9** | **3.5** | **3.7** | 1.2 | 1.1 | 1.3 | * |
| An09g05490 | *ERP3* | ERP3 (YDL018c) – p24 family protein | **2.3** | **2.8** | **3.0** | 1.2 | 1.1 | 1.3 | * |
| An04g08830 | *EMP47* | EMP47 (YFL048c) – COPII vesicle membrane component | **2.9** | **3.0** | **3.1** | 1.0 | 1.0 | 1.1 | * |
| An08g03960 | *ERV29* | ERV29 (YGR284c) – glycoprotein cargo receptor | **2.8** | **3.5** | **3.4** | 1.2 | 1.0 | 1.2 | * |
| An02g04250 |  | similarity to protein p58 - *Rattus norvegicus* | **3.5** | **4.2** | **4.3** | 1.2 | 1.0 | 1.2 | * |
| An03g04940 | *ERV41* | ERV41 (YML067c) – involved in COPII vesicle fusion | **4.8** | **4.6** | **4.9** | 1.0 | 1.1 | 1.0 | * |
| An01g04320 | *ERV46* | ERV46 (YAL042w) – involved in COPII vesicle fusion | **3.9** | **4.8** | **4.6** | 1.2 | 1.0 | 1.2 | * |
| **Golgi to endosome transport** | | | | | | | | | |
| An01g07330 |  | similarity to brefeldin A-inhibited guanine nucleotide-exchange protein 2 - *Homo sapiens* | **2.0** | **1.8** | **1.9** | -1.1 | 1.1 | 1.0 | GO:0006895 |
| An08g01410 | *SFT2* | strong similarity to ER-Golgi transport protein Sft2 - *Saccharomyces cerevisiae* | **2.0** | **2.5** | **2.6** | 1.2 | 1.1 | 1.3 | GO:0006895 |
| An07g08220 |  | strong similarity to clathrin associated epsin 2A - *Homo sapiens* | **1.7** | **1.5** | **1.4** | -1.1 | 1.0 | -1.2 | GO:0006895 |
| An11g04750 | *dopA* | strong similarity to developmental regulator of asexual and sexual reproduction dopA - *Aspergillus* | **2.2** | **2.2** | **2.3** | 1.0 | 1.0 | 1.1 | GO:0006895 |
| An07g08220 |  | strong similarity to clathrin associated epsin 2A - *Homo sapiens* | **1.7** | **1.5** | **1.4** | -1.1 | 1.0 | -1.2 | * |
| An16g03420 | *SYS1* | similarity to multicopy suppressor Sys1 - *Saccharomyces cerevisiae* | 1.3 | **1.8** | **1.8** | 1.3 | 1.0 | 1.4 | ***** |
| **Endocytosis** | | | | | | | | | |
| An18g03660 | *PRK1* | similarity to ser/thr protein kinase Prk1 - *Saccharomyces cerevisiae* | **1.4** | **1.4** | **1.6** | 1.0 | 1.1 | 1.1 | GO:0006897 |
| An07g08830 | *YAP180* | strong similarity to adaptor protein Yap180 -*Saccharomyces cerevisiae* | **1.5** | **1.7** | **1.7** | 1.1 | 1.0 | 1.1 | GO:0006897 |
| An07g10420 | *CDC50* | strong similarity to cell division cycle protein Cdc50 - *Saccharomyces cerevisiae* | **1.5** | **1.7** | **1.9** | 1.1 | 1.1 | 1.2 | GO:0006897 |
| An12g00120 | *SWH1* | strong similarity to hypothetical oxysterol binding protein homologue Swh1 - *Saccharomyces cerevisiae* | **1.5** | **1.5** | **1.6** | 1.0 | 1.0 | 1.0 | GO:0006897 |
| An17g02290 | *MYO2* | strong similarity to myosin Myo2 -*Saccharomyces cerevisiae* | **1.7** | **1.9** | **2.0** | 1.1 | 1.0 | 1.2 | GO:0006897 |
| An16g08470 |  | similarity to hypothetical cell growth regulator OS-9 - *Homo sapiens* | **5.2** | **5.4** | **5.7** | 1.0 | 1.1 | 1.1 | GO:0006897 |
| An01g07330 |  | similarity to brefeldin A-inhibited guanine nucleotide-exchange protein 2 - *Homo sapiens* | **2.0** | **1.8** | **1.9** | -1.1 | 1.1 | 1.0 | GO:0006897 |
| An15g01510 | *DRS2* | strong similarity to P-type ATPase Drs2 -Saccharomyces cerevisiae | **2.1** | **1.8** | **1.9** | -1.1 | 1.0 | -1.1 | GO:0006897 |
| An02g03460 |  | (BAR) domain of *Saccharomyces cerevisiae* Golgi vesicle protein of 36 kDa and similar proteins | **2.1** | **2.4** | **2.6** | 1.2 | 1.1 | 1.2 | GO:0006897 |
| An05g00200 | *ARK1* | strong similarity to ankyrin repeat-containing protein Akr1 - *Saccharomyces cerevisiae* | **2.4** | **2.3** | **2.3** | 1.0 | 1.0 | 1.0 | GO:0006897 |
| An15g04490 | *myoA* | strong similarity to myosin I myoA -*Aspergillus nidulans* | **1.7** | **1.6** | **1.6** | -1.1 | 1.0 | -1.1 | GO:0006897 |
| An04g07040 | *CLC1* | strong similarity to clathrin light chain Clc1 - *Saccharomyces cerevisiae* | **1.6** | **1.7** | **1.7** | 1.1 | 1.0 | 1.1 | GO:0006897 |
| An02g07780 | *ARF6* | strong similarity to ADP-ribosylation factor 6 ARF6 - *Saccharomyces cerevisiae* | **1.6** | **1.8** | **1.9** | 1.2 | 1.0 | 1.2 | GO:0006897 |

* Not present in GO-list; GO:0006890: retrograde vesicle-mediated transport. Golgi to ER; GO:0006901: Vesicle coat; GO:0048199: Vesicle targeting to, from or within Golgi; GO:0090114: COPII-coated vesicle budding; GO:0006895: Golgi to endosome transport. GO:0006897: Endocytosis. Values in bold represent a significant fold change with a FDR<0.005.
