# Supplementary material for: Genome-wide expression analysis upon constitutive activation of the HacA bZIP transcription factor in Aspergillus niger reveals a coordinated cellular response to counteract ER stress
Source: BMC Genomics. 2012 Jul 30;13:350. doi: 10.1186/1471-2164-13-350 (PMC3472299; doi:10.1186/1471-2164-13-350)
Supplement: Additional file 12 — Expression values of selected genes related to enriched GO terms associated with glycosylation processes. Subset of all differentially expressed genes (Additional file 3) [file 1471-2164-13-350-S12.doc]

Additional file 12: Expression values of selected genes related to enriched GO terms associated with glycosylation processes.

| **Gene ID** | **Gene name: *A. niger* or *S. cerevisiae*** | **Description** | **Fold change** | | | | | | **GO-term** |
| --- | --- | --- | --- | --- | --- | --- | --- | --- | --- |
|  |  |  | **HacA^CA-1^/**  **HacA^WT^** | **HacA^CA-2^/**  **HacA^WT^** | **HacA^CA-3^/**  **HacA^WT^** | **HacA^CA-2^/**  **HacA^CA-1^** | **HacA^CA-2^/**  **HacA^CA-3^** | **HacA^CA-3^/**  **HacA^CA-1^** | **Biological Process** |
| **Oligosaccharide-lipid intermediate assembly** | | | | | | | | | |
| An02g12630 | *ALG6* | strong similarity to glucosyltransferase ALG6 - *Saccharomyces cerevisiae* | **2.9** | **2.8** | **2.9** | 1.0 | 1.0 | 1.0 | GO:0006490 |
| An18g05910 |  | strong similarity to probable glycosyl transferase SPCC330.08 - *Schizosaccharomyces pombe* | **6.7** | **6.9** | **7.3** | 1.0 | 1.1 | 1.1 | GO:0006490 |
| An04g08820 | *ALG8* | strong similarity to glucosyltransferase ALG8 - *Saccharomyces cerevisiae* | **2.8** | **2.5** | **2.4** | -1.1 | 1.0 | -1.1 | GO:0006490 |
| An14g05910 | *ALG2* | similar to mannosyltransferase ALG2 - *Saccharomyces cerevisiae* | **4.3** | **5.1** | **5.4** | 1.2 | 1.1 | 1.3 | GO:0006490 |
| An18g02360 | *ALG3* | similar to mannosyltransferase ALG3 - *Saccharomyces cerevisiae* | **3.8** | **4.6** | **4.7** | **1.2** | 1.0 | **1.2** | GO:0006490 |
| An06g01100 |  | strong similarity to mannosyltransferase mntA - *Dictyostelium discoideum* | **2.3** | **2.5** | **2.5** | 1.1 | 1.0 | 1.1 | GO:0006490 |
| An16g04330 | *DPM1* | mannose phospho-dolichol synthase DPMA- *Saccharomyces cerevisiae* | **2.8** | **3.5** | **3.6** | **1.3** | 1.0 | **1.3** | * |
| An01g05200 | *DPM2* | strong similarity to dolichyl-phosphate mannosyltransferase DPM2 | **2.0** | **3.1** | **3.3** | **1.6** | 1.0 | **1.7** | * |
| An14g00270 |  | weak similarity to dolichol-phosphate-mannose  synthase DPM3 - Homo sapiens | **1.7** | **2.1** | **2.1** | **1.3** | 1.0 | **1.3** | * |
| An03g04410 | *ALG5* | similar to glucosyltransferase ALG5 - *Saccharomyces cerevisiae* | **4.1** | **4.5** | **4.7** | 1.1 | 1.0 | 1.1 | * |
| An02g03240 | *ALG7* | similar to N-acetylglucosaminephosphotransferase ALG7- *Saccharomyces cerevisiae* | **5.7** | **7.5** | **7.9** | 1.3 | 1.1 | **1.4** | * |
| An02g14940 | *RFT1* | similar to flippase RFT1 - *Saccharomyces cerevisiae* | **3.6** | **4.2** | **4.5** | **1.2** | 1.1 | **1.2** | * |
| An08g07020 | *ALG9* | similarity to mannosyl transferase ALG9 - *Saccharomyces cerevisiae* | **2.6** | **2.4** | **2.5** | -1.1 | 1.0 | 1.0 | * |
| An04g03960 | *SEC59* | strong similarity to dolichol kinase Sec59 - *Saccharomyces cerevisiae* | **1.5** | **1.3** | **1.3** | -1.1 | 1.0 | -1.1 | * |
| An04g03130 |  | strong similarity to putative flipping of Dol-P-Man/Glc | **1.3** | **1.5** | **1.6** | 1.2 | 1.0 | **1.2** | * |
| An01g08460 | *ALG12* | strong similarity to mannosyltransferase ALG12 *-Saccharomyces cerevisiae* | **3.0** | **2.5** | **2.6** | -1.2 | 1.0 | -1.1 | GO:0000009 |
| An02g02980 | *ALG10* | strong similarity to glucosyltransferase ALG10 - *Saccharomyces cerevisiae* | **2.8** | **2.7** | **2.4** | 1.0 | -1.1 | -1.1 | * |
| **Oligosaccharyl transferase complex** | | | | | | | | | |
| An02g14560 | *OST1* | strong similarity to subunit of oligosacharyltransferase complex | **3.6** | **3.6** | **3.7** | 1.0 | 1.0 | 1.0 | * |
| An07g04190 | *WBP1* | strong similarity to subunit of oligosacharyltransferase complex | **3.4** | **3.7** | **3.8** | 1.1 | 1.0 | 1.1 | * |
| An18g03920 | *OST2* | strong similarity to subunit of oligosacharyltransferase complex | **2.8** | **3.4** | **3.7** | **1.2** | 1.1 | **1.3** | * |
| An02g14930 | *OST3* | strong similarity to subunit of oligosacharyltransferase complex | **2.7** | **3.0** | **3.2** | **1.1** | 1.0 | **1.2** | * |
| An16g08570 | *SST2* | strong similarity to subunit of oligosacharyltransferase complex | **3.3** | **3.3** | **3.5** | 1.0 | 1.1 | 1.1 | * |
| An08g07485** |  | strong similarity to subunit of oligosacharyltransferase complex |  |  |  |  |  |  | * |
| **UDP-glucose transport** | | | | | | | | | |
| An03g06940 |  | similarity to UPD-GlcNAc transporter Golgi MNN2-2 - *Kluyveromyces lactis* | **2.3** | **2.7** | **2.7** | 1.2 | 1.0 | 1.2 | GO:0015786 |
| An11g02020 |  | similarity to glucose-6-phosphate/phosphate-translocator GPT - *Zea mays* | **1.8** | **1.8** | **1.9** | 1.0 | 1.0 | 1.1 | GO:0015786 |
| An18g04260 |  | similarities to some probable UDP-galactose transporters | **6.8** | **8.0** | **8.5** | 1.2 | 1.1 | 1.2 | GO:0015786 |
| An08g10400 |  | strong similarity to UDP-Gal transporter | 1.4 | **1.5** | 1.4 | 1.0 | 1.0 | 1.0 | * |
| An17g02140 |  | strong similarity to GDP-Man transporter | 1.3 | 1.4 | 1.4 | 1.1 | 1.0 | 1.1 | * |
| An11g02020 |  | strong similarity to GDP-Man transporter | **1.8** | **1.8** | **1.9** | 1.0 | 1.0 | 1.1 | * |
| **Protein amino acid O-linked glycosylation** | | | | | | | | | |
| An07g10350 | *pmtA* | protein O-mannosyl transferase pmtA - Aspergillus niger | **2.3** | **2.5** | **2.6** | 1.1 | 1.0 | 1.1 | GO:0006493 |
| An16g08490 | *PMT4* | strong similarity to dolichyl-phosphate-D-mannose--protein O-mannosyltransferase Pmt4 - *Saccharomyces cerevisiae* | **2.1** | **1.9** | **2.0** | -1.1 | 1.1 | 1.0 | GO:0006493 |
| An11g09890 |  | strong similarity to mannosyltransferase 1 PMT1 - *Candida albicans* | **3.1** | **3.0** | **2.9** | 1.0 | 1.0 | -1.1 | GO:0006493 |
| An14g03910 |  | strong similarity to alpha-1,2-mannosyltransferase kre2 - *Candida albicans* | **2.1** | **2.3** | **2.5** | 1.1 | 1.1 | 1.2 | GO:0006493 |
| **GPI anchor biosynthesis** | | | | | | | | | |
| An16g03530 | *GPI1* | Protein involved in the synthesis of GlcNAc-PI. the first intermediate in the synthesis of (GPI) anchors | **1.7** | **1.9** | **1.9** | 1.1 | 1.0 | 1.1 | * |
| An02g13570 | *GPI2* | Protein involved in the synthesis of GlcNAc-PI. the first intermediate in the synthesis of (GPI) anchors | **1.5** | **1.6** | **1.5** | 1.0 | 1.0 | 1.0 | * |
| An01g09910 | *GPI3* | Protein involved in the synthesis of GlcNAc-PI. the first intermediate in the synthesis of (GPI) anchors | 1.1 | **1.3** | **1.3** | 1.2 | 1.0 | 1.2 | * |
| An02g09230 | *GPI15* | Protein involved in the synthesis of GlcNAc-PI. the first intermediate in the synthesis of (GPI) anchors | 1.1 | 1.0 | 1.1 | 1.0 | 1.0 | 1.0 | * |
| An16g01530 | *GPI19* | Protein involved in the synthesis of GlcNAc-PI. the first intermediate in the synthesis of (GPI) anchors | **1.4** | **1.3** | **1.3** | -1.1 | 1.0 | 1.0 | * |
| An14g06640 | *GPI12* | ER membrane protein involved in the second step of glycosylphosphatidylinositol (GPI) anchor assembly. the de-N-acetylation of the N-acetylglucosaminylphosphatidylinositol intermediate; | **1.7** | **1.7** | **1.6** | 1.0 | 1.0 | 1.0 | * |
| An01g12990 | *GWT1* | Protein involved in the inositol acylation of glucosaminyl phosphatidylinositol (GlcN-PI) to form glucosaminyl(acyl)phosphatidylinositol (GlcN(acyl)PI). an intermediate in the biosynthesis of glycosylphosphatidylinositol (GPI) anchors. third step | **2.7** | **3.0** | **3.4** | 1.1 | 1.1 | **1.3** | * |
| An12g01880 | *GPI14* | Glycosylphosphatidylinositol-alpha 1.4 mannosyltransferase I. involved in GPI anchor biosynthesis. requires Pbn1p for function. forth step | **1.4** | **1.4** | **1.5** | 1.0 | 1.1 | 1.1 | * |
| An17g00780 | *PBN1* | Essential component of glycosylphosphatidylinositol-mannosyltransferase I. | 1.0 | -1.1 | -1.1 | 1.0 | 1.0 | 1.0 | * |
| An14g00900 | *MCD4* | Protein involved in glycosylphosphatidylinositol (GPI) anchor synthesis; multimembrane-spanning protein that localizes to the endoplasmic reticulum; step 5 | **2.1** | **1.9** | **1.9** | -1.1 | 1.0 | -1.1 | * |
| An16g01530 | *GPI18* | mannosyltransferase that transfers the second mannose in glycosylphosphatidylinositol biosynthesis. step 6 | **1.4** | **1.3** | **1.3** | -1.1 | 1.0 | 1.0 | * |
| An04g04110 | *GPI10* | Integral membrane protein involved in glycosylphosphatidylinositol (GPI) anchor synthesis; putative alpha 1.2 mannosyltransferase required for addition of the third mannose onto the GPI core structure. step 7 | **1.5** | **1.3** | **1.3** | -1.1 | 1.0 | -1.1 | * |
| An10g00480 | *GPI11* | ER membrane protein involved in a late step of glycosylphosphatidylinositol (GPI) anchor assembly; involved in the addition of phosphoethanolamine to the multiply mannosylated GPI intermediat. step 9 | **1.3** | 1.1 | 1.1 | **-1.1** | 1.0 | **-1.2** | * |
| An09g02800 | *GPI13* | ER membrane localized phosphoryltransferase that adds phosphoethanolamine onto the third mannose residue of the glycosylphosphatidylinositol (GPI) anchor precursor. step 9 | **1.2** | -1.1 | -1.1 | **-1.4** | 1.0 | **-1.2** | * |
| An04g05100 | *GPI13* | ER membrane localized phosphoryltransferase that adds phosphoethanolamine onto the third mannose residue of the glycosylphosphatidylinositol (GPI) anchor precursor. step 9 | 1.0 | 1.1 | 1.0 | 1.0 | -1.1 | -1.1 | * |
| An08g10720 | *GAA1* | Subunit of the GPI (glycosylphosphatidylinositol):protein transamidase complex | **1.4** | 1.0 | 1.0 | **-1.4** | 1.0 | **-1.4** | * |
| An04g02650 | *GAB1* | Subunit of the GPI (glycosylphosphatidylinositol):protein transamidase complex | **3.0** | **3.3** | **3.5** | 1.1 | 1.1 | 1.2 | * |
| An11g06770 | *GPI16* | Subunit of the GPI (glycosylphosphatidylinositol):protein transamidase complex | **2.4** | **2.2** | **2.3** | -1.1 | 1.1 | 1.0 | * |
| An07g09270 | *GPI17* | Subunit of the GPI (glycosylphosphatidylinositol):protein transamidase complex | 1.1 | 1.1 | 1.2 | 1.0 | 1.0 | 1.0 | * |
| An01g13530 | *GPI8* | Subunit of the GPI (glycosylphosphatidylinositol):protein transamidase complex | **1.5** | **1.6** | **1.6** | 1.0 | 1.0 | 1.1 | * |
| An15g00030 | *ScLas21* | Integral plasma membrane protein involved in the synthesis of the glycosylphosphatidylinositol (GPI) core structure; mutations affect cell wall integrity | **1.6** | **1.6** | **1.6** | 1.0 | 1.0 | 1.0 | * |

* Not present in GO-list; GO:0006490: oligosaccharide-lipid intermediate assembly; GO:0000009 alpha-1.6-mannosyltransferase activity; GO:0006493 protein amino acid O-linked glycosylation. Values in bold represent a significant fold change with a FDR<0.005.
