# Supplementary material for: Genome-wide expression analysis upon constitutive activation of the HacA bZIP transcription factor in Aspergillus niger reveals a coordinated cellular response to counteract ER stress
Source: BMC Genomics. 2012 Jul 30;13:350. doi: 10.1186/1471-2164-13-350 (PMC3472299; doi:10.1186/1471-2164-13-350)
Supplement: Additional file 13 — Expression values of selected genes related to enriched GO terms associated with lipid metabolic processes. Subset of all differentially expressed genes (Additional file 3). [file 1471-2164-13-350-S13.doc]

Additional file 13: Expression values of selected genes related to enriched GO terms associated with lipid metabolic processes.

| **Gene ID** | **Gene name: *A. niger* or *S. cerevisiae*** | **Description** | **Fold change** | | | | | | **GO-term** |
| --- | --- | --- | --- | --- | --- | --- | --- | --- | --- |
|  |  |  | **HacA^CA-1^/**  **HacA^WT^** | **HacA^CA-2^/**  **HacA^WT^** | **HacA^CA-3^/**  **HacA^WT^** | **HacA^CA-2^/**  **HacA^CA-1^** | **HacA^CA-2^/**  **HacA^CA-3^** | **HacA^CA-3^/**  **HacA^CA-1^** | **Biological Process** |
| **Lipid metabolism** | | | | | | | | | |
| An08g00560 |  | strong similarity to phosphatidyl-N-methylethanolamine N-methyltransferase Opi3 | **1.7** | **2.0** | **1.8** | 1.2 | -1.1 | 1.1 | GO:0008610 |
| An15g00630 | *SUR1* | strong similarity to sphingolipid metabolism Sur1 - *Saccharomyces cerevisiae* | **1.9** | **2.0** | **2.1** | 1.0 | 1.1 | 1.1 | GO:0008610 |
| An14g03360 |  | strong similarity to choline-transport mutant SCT1 supressor protein | **1.7** | **1.7** | **1.7** | 1.0 | 1.0 | 1.0 | GO:0008610 |
| An15g01460 | *CWH8* | strong similarity to Cwh8 - *Saccharomyces cerevisiae* | **2.5** | **3.1** | **3.3** | 1.2 | 1.1 | 1.4 | GO:0008610 |
| An04g00600 | *LAG1* | strong similarity to hypothetical ER-to-Golgi transporter Lag1 - *Saccharomyces cerevisiae* | **2.1** | **2.4** | **2.4** | 1.2 | 1.0 | 1.2 | GO:0008610 |
| An07g09690 |  | strong similarity to sterol C-24(28) reductase sts1p - *Schizosaccharomyces pombe* | **1.7** | **1.8** | **1.7** | 1.0 | 1.0 | 1.0 | GO:0008610 |
| An09g00620 |  | similarity to estradiol 17-beta-dehydrogenase HSD17B1 - *Rattus norvegicus* | **2.8** | **4.2** | **4.1** | 1.5 | 1.0 | 1.5 | GO:0008610 |
| An02g09910 | *FEN1* | strong similarity to fatty acid elongase Fen1 - *Saccharomyces cerevisiae* | **1.7** | **1.6** | **1.6** | -1.1 | 1.0 | 1.0 | GO:0008610 |
| An04g05250 | *RER2* | strong similarity to undecaprenyl phosphate synthase Rer2 from patent WO200121650-A2 - *Saccharomyces cerevisiae* | **3.1** | **3.9** | **4.2** | 1.3 | 1.1 | 1.3 | GO:0008610 |
| An13g00040 |  | 1-acylglycerol-3-phosphate O-acyltransferase | **2.2** | **2.8** | **2.8** | 1.3 | 1.0 | 1.3 | GO:0008610 |
| An01g07640 |  | conserved hypothetical protein similar to alkaline phytoceramidase | **2.1** | **2.4** | **2.5** | 1.1 | 1.0 | 1.2 | GO:0008610 |
| An01g14140 |  | strong similarity to CDPdiacylglycerol--inositol 3-phosphatidyltransferase | **2.4** | **3.2** | **3.2** | 1.3 | 1.0 | 1.4 | GO:0008610 |
| An14g00900 | *MCD4* | strong similarity to glycosylphosphatidylinositol anchor synthesis protein Mcd4 - *Saccharomyces cerevisiae* | **2.1** | **1.9** | **1.9** | -1.1 | 1.0 | -1.1 | GO:0008610 |
| An01g12990 | *GWT1* | GPI anchor biosynthesis protein Gwt1 | **2.7** | **3.0** | **3.4** | 1.1 | 1.1 | 1.3 | GO:0008610 |
| An02g13410 |  | similar to acetyl-coenzyme A transporter AT-1 - *Homo sapiens* | **6.1** | **6.4** | **6.6** | 1.0 | 1.0 | 1.1 | * |
| An08g04990 |  | strong similarity to carnitine acetyl transferase FacC - *Emericella nidulans* | **2.7** | **2.5** | **2.5** | -1.1 | 1.0 | -1.1 | * |
| An04g01320 |  | strong similarity fatty acid desaturase from patent WO9846764-A1 - *Homo sapiens* | **3.1** | **3.1** | **3.3** | 1.0 | 1.1 | 1.1 | * |
| An01g06800 | *YPC1* | strong similarity to alkaline ceramidase Ypc1p - *Saccharomyces cerevisiae* | **6.1** | **10.5** | **11.4** | **1.7** | 1.1 | **1.9** | * |
| An16g06350 |  | strong similarity to delta(6)-desaturase - *Mucor rouxii* | **3.3** | **4.5** | **5.2** | 1.4 | 1.1 | 1.6 | * |
| An03g06410 | *ERG25* | strong similarity to methyl sterol oxidase ERG25 - *Saccharomyces cerevisiae* | **4.2** | **6.4** | **7.3** | 1.5 | 1.1 | 1.7 | * |
| An11g03230 | *ERG5* | strong similarity to cytochrome P450 erg5 - *Saccharomyces cerevisiae* | 1.5 | **1.6** | **1.7** | 1.1 | 1.0 | 1.1 | * |
| An04g02050 |  | weak similarity to insulin induced protein 1 INSIG1 - *Homo sapiens* | **1.6** | **1.6** | **1.7** | 1.0 | 1.1 | 1.1 | * |
| An03g02820 | *CVT17* | strong similarity to putative lipase Cvt17p - *Saccharomyces cerevisiae* | **1.8** | **2.0** | **2.1** | 1.1 | 1.1 | 1.2 | * |
| An14g01590 |  | strong similarity to sterol transmethylase ERG6 - *Candida albicans* | 1.4 | **1.4** | **1.4** | 1.0 | 1.0 | 1.0 | * |
| An16g08180 |  | similarity to palmitylated serine/threonine kinase PKL12 - *Mus musculus* | **1.8** | **1.9** | **2.1** | 1.0 | 1.1 | 1.1 | * |
| An04g07120 | *FRM2* | similarity to protein FRM2 involved in fatty acid regulation - *Saccharomyces cerevisiae* | **1.7** | **1.8** | **1.8** | 1.1 | 1.0 | 1.1 | * |
| An07g06770 |  | strong similarity to delta-6 desaturase - *Mucor rouxii* | **1.8** | **1.8** | **2.0** | 1.0 | 1.1 | 1.1 | * |
| An15g05840 | *UPC2* | weak similarity to regulatory protein UPC2 - *Saccharomyces cerevisiae* | **2.0** | **1.8** | **1.8** | -1.1 | 1.0 | -1.1 | * |
| An07g09190 | *FAT1* | strong similarity to very long-chain fatty acyl-CoA synthetase FAT1 - *Saccharomyces cerevisiae* | 1.7 | **2.5** | **2.7** | 1.5 | 1.1 | 1.6 | * |
| **Phosphoinositide metabolism** | | | | | | | | | |
| An13g00110 |  | strong similarity to 1-phosphatidylinositol 4-kinase Stt4 | **1.8** | **1.9** | **2.0** | 1.1 | 1.0 | 1.1 | GO:0030384 |
| An09g01240 |  | strong similarity to phospholipase B - | **2.1** | **1.8** | **1.8** | -1.1 | 1.0 | -1.2 | GO:0030384 |
| An04g02480 |  | strong similarity to lipid phosphoinositide phosphatase Sac1 | **1.9** | **1.8** | **2.0** | 1.0 | 1.1 | 1.1 | GO:0030384 |
| An11g06770 |  | GPI transamidase component Gpi16 | **2.4** | **2.2** | **2.3** | -1.1 | 1.1 | 1.0 | GO:0008610  GO:0030384 |
| An04g00340 | *ITR2* | strong similarity to myo-inositol transport protein ITR2 - *Saccaromyces cerevisiae* | 2.2 | 3.0 | 3.4 | 1.3 | 1.1 | 1.5 | * |
| An14g06640 |  | strong similarity to N-acetylglucosaminyl-phosphatidylinositol deacetylase PIGL - *Rattus norvegicus* | 1.7 | 1.7 | 1.6 | 1.0 | 1.0 | 1.0 | * |

* Not present in GO-list ; GO:0008610: lipid biosynthetic process; GO:0030384: phosphoinositide metabolic process. Values in bold represent a significant fold change with a FDR<0.005.
