# Supplementary material for: Genome-wide expression analysis upon constitutive activation of the HacA bZIP transcription factor in Aspergillus niger reveals a coordinated cellular response to counteract ER stress
Source: BMC Genomics. 2012 Jul 30;13:350. doi: 10.1186/1471-2164-13-350 (PMC3472299; doi:10.1186/1471-2164-13-350)
Supplement: Additional file 14 — Expression values of selected genes related to the GO terms “hydrolase activity”, “glutathione catabolic processes” and “vacuolar acidification”. Subset of all differentially expressed genes (Additional file 3). [file 1471-2164-13-350-S14.doc]

Additional file 14: Expression values of selected genes related to the GO terms “hydrolase activity”, “glutathione catabolic processes” and “vacuolar acidification”.

| **Gene ID** | **Gene name: *A. niger* or *S. cerevisiae*** | **Description** | **Fold change** | | | | | | **GO-term** |
| --- | --- | --- | --- | --- | --- | --- | --- | --- | --- |
|  |  |  | **HacA^CA-1^/**  **HacA^WT^** | **HacA^CA-2^/**  **HacA^WT^** | **HacA^CA-3^/**  **HacA^WT^** | **HacA^CA-2^/**  **HacA^CA-1^** | **HacA^CA-2^/**  **HacA^CA-3^** | **HacA^CA-3^/**  **HacA^CA-1^** | **Biological Process** |
| **Hydrolase activity** | | | | | | | | | |
| An04g00360 | *SEC13* | strong similarity to transport vesicle formation protein Sec13 - *Saccharomyces cerevisiae* | **2.1** | **2.1** | **2.1** | 1.0 | 1.0 | 1.0 | GO:0051345 |
| An08g10570 |  | strong similarity to hypothetical membrane protein YLR386w- *Saccharomyces cerevisiae* | **1.5** | **1.5** | **1.5** | 1.0 | 1.0 | 1.0 | GO:0051345 |
| **Glutathione catabolic process** | | | | | | | | | |
| An08g04260 |  | glutamine amidotransferase | **1.8** | **2.1** | **2.4** | 1.2 | 1.1 | 1.3 | GO:0006751 |
| An11g11180 |  | glutamate carboxypeptidase | **1.5** | **1.5** | **1.5** | 1.0 | 1.0 | 1.0 | GO:0006751 |
| An13g01300 |  | strong similarity to gamma-glutamyl transpeptidase GGT1 | **1.6** | **1.7** | **1.7** | 1.1 | 1.0 | 1.1 | GO:0006751 |
| **Vacuolar acidification** | | | | | | | | | |
| An04g05310 | *VHP1* | strong similarity to vacuolar H(+)-transporting ATPase subunit Vph1 - *Saccharomyces* | **1.5** | **1.8** | **1.7** | 1.2 | 1.0 | 1.2 | GO:0007035 |
| An02g02020 |  | strong similarity to vacuolar H(+)-transporting ATPase subunit B Vma2 | **1.6** | **1.6** | **1.5** | 1.0 | 1.0 | 0.9 | GO:0007035 |
| An17g01550 | *NHA2* | strong similarity to Na+-H+ antiporter Nha2 - *Saccharomyces cerevisiae* | **3.2** | **3.6** | **3.8** | 1.1 | 1.0 | 1.2 | GO:0007035 |
| An08g06750 | *RAV1* | strong similarity to regulator protein Rav1 -*Saccharomyces cerevisiae* | **1.9** | **2.0** | **2.0** | 1.1 | 1.0 | 1.0 | GO:0007035 |

GO:0051345: positive regulation of hydrolase activity; GO:0006751: glutathione catabolic process; GO:0007035: vacuolar acidification. Values in bold represent a significant fold change with a FDR<0.005.
