# Supplementary material for: Genome-wide expression analysis upon constitutive activation of the HacA bZIP transcription factor in Aspergillus niger reveals a coordinated cellular response to counteract ER stress
Source: BMC Genomics. 2012 Jul 30;13:350. doi: 10.1186/1471-2164-13-350 (PMC3472299; doi:10.1186/1471-2164-13-350)
Supplement: Additional file 15 — Expression values of selected down-regulated genes related to enriched GO terms. Subset of all differentially expressed genes (Additional file 3). [file 1471-2164-13-350-S15.doc]

Additional file 15: Expression values of selected down-regulated genes related to enriched GO terms.

| **Gene ID** | **Gene name: *A. niger* or *S. cerevisiae*** | **Description** | **Fold change** | | | | | | **GO-term** |
| --- | --- | --- | --- | --- | --- | --- | --- | --- | --- |
|  |  |  | **HacA^CA-1^/**  **HacA^WT^** | **HacA^CA-2^/**  **HacA^WT^** | **HacA^CA-3^/**  **HacA^WT^** | **HacA^CA-2^/**  **HacA^CA-1^** | **HacA^CA-2^/**  **HacA^CA-3^** | **HacA^CA-3^/**  **HacA^CA-1^** | **Biological Process** |
| **Glycolysis/Gluconeogenesis** | | | | | | | | | |
| An04g02090 | *pyc* | pyruvate carboxylase pyc - *Aspergillus niger* | **-2** | **-2** | **-2** | 1.0 | 1.0 | 1.1 | GO:0006096  GO:0006094 |
| An07g04300 |  | strong similarity to 3-methylcrotonyl-CoA carboxylase (MCC) biotin-containing alpha subunit MCCA - *Homo sapiens* | **-1.7** | **-2** | **-2** | 1.0 | 1.0 | -1.1 | GO:0006096  GO:0006094 |
| An08g02260 | *pgkA* | strong similarity to phosphoglycerate kinase pgkA - *Aspergillus nidulans* | **-1.4** | **-1.7** | **-1.7** | -1.1 | -1.1 | -1.2 | GO:0006096  GO:0006094 |
| An11g02550 |  | strong similarity to phosphoenolpyruvate carboxykinase KlPck1 - *Kluyveromyces lactis* | **-3.3** | **-3.3** | **-3.3** | 1.1 | 1.0 | 1.1 | GO:0006096  GO:0006094  GO:0006083 |
| An02g14380 | hxk | hexokinase hxk - *Aspergillus niger* | **-1.7** | **-1.7** | **-1.7** | 1.0 | 1.0 | 1.0 | GO:0006096  GO:0006094  GO:0008645 |
| An16g05420 | *PGI1* | strong similarity to glucose-6-phosphate isomerase Pgi1 - *Saccharomyces cerevisiae* | **-1.7** | **-1.4** | **-1.7** | 1.0 | -1.1 | -1.1 | GO:0006096  GO:0006094  GO:0006740 |
| **Alcohol catabolic/metabolic process** | | | | | | | | | |
| An02g12140 | *gsdA* | glucose-6-phosphat 1-dehydrogenase gsdA - *Aspergillus niger* | **-1.4** | **-1.7** | **-1.7** | 1.0 | 1.0 | 1.0 | GO:0019321  GO:0006979  GO:0006740 |
| An02g01140 | *facB* | weak similarity to acetate regulatory gene FacB - *Aspergillus niger* | **-2.5** | **-2** | -1.7 | 1.3 | 1.1 | 1.4 | * |
| An12g00030 | *SOR1* | strong similarity to L-iditol 2-dehydrogenase Sor1 - *Saccharomyces cerevisiae* | **-2.5** | **-2** | **-1.7** | 1.4 | 1.1 | 1.5 | GO:0019321 |
| An04g02610 |  | strong similarity to succinate-semialdehyde dehydrogenase NAD(P)+ gabD - *Escherichia coli* | **-2** | -1.7 | -1.7 | 1.3 | -1.1 | 1.1 | * |
| An02g02060 |  | strong similarity to alcohol dehydrogenase of patent EP 0845532-A 19 | **-2** | **-2** | **-2** | 1.1 | 1.0 | 1.1 | * |
| An04g05620 | *facA* | strong similarity to acetate--CoA ligase facA - *Aspergillus nidulans* | **-2** | **-2** | **-2.5** | 1.0 | -1.1 | -1.1 | GO:0006083  GO:0019679 |
| An12g04640 |  | similarity to sequence 28 from patent WO0032789 - *Mangifera indica* | **-1.7** | **-1.4** | **-1.7** | 1.1 | -1.1 | 1.0 | GO:0006083 |
| An16g07110 | *ACH1* | strong similarity to acetyl-CoA hydrolase Ach1 - *Saccharomyces cerevisiae* | **-5** | **-5** | **-5** | 1.1 | 1.0 | 1.0 | GO:0006083 |
| An01g07030 |  | strong similarity to 2-ketoaldonate reductase yiaE - *Escherichia coli* | **-1.7** | **-2** | **-2** | -1.1 | -1.1 | -1.2 | GO:0019679 |
| An10g00510 | *SFA1* | strong similarity to alcohol dehydrogenase Sfa1 - *Saccharomyces cerevisiae* | **-1.7** | **-1.7** | **-1.7** | 1.0 | 1.0 | 1.0 | GO:0046185  GO:0006071  GO:0033609 |
| An17g01530 | *adhA* | alcohol-dehydrogenase adhA from patent WO8704464-A - *Aspergillus niger* | **-5** | **-5** | **-5** | 1.1 | 1.0 | 1.1 | GO:0046185  GO:0006071  GO:0006567  GO:0033609 |
| An07g08390 |  | strong similarity to carboxyphosphonoenolpyruvate mutase - *Streptomyces hygroscopicus* | **-1.7** | **-1.7** | **-1.7** | 1.1 | -1.1 | 1.0 | GO:0033609 |
| **Carboxylic Acid Metabolism** | | | | | | | | | |
| An07g02160 | *MDH1* | strong similarity to mitochondrial malate dehydrogenase Mdh1 - *Saccharomyces cerevisiae* | **-1.4** | **-1.7** | **-1.7** | -1.1 | 1.0 | -1.2 | GO:0006099 |
| An08g10530 | *ACO1* | strong similarity to mitochondrial aconitate hydratase Aco1 - *Saccharomyces cerevisiae* | **-1.4** | **-2** | **-2.5** | -1.2 | -1.1 | -1.2 | GO:0006099 |
| An12g07850 |  | strong similarity to fumarate hydratase fumR - *Rhizopus oryzae* | **-1.4** | **-2** | **-2** | -1.2 | 1.0 | -1.2 | GO:0006099 |
| An14g04400 | *SDH2* | strong similarity to succinate dehydrogenase iron-sulfur protein subunit Sdh2 - *Saccharomyces cerevisiae* | **-1.7** | **-1.7** | **-2** | -1.1 | 1.0 | -1.2 | GO:0006099 |
| An15g01920 | *mcsA* | strong similarity to methylcitrate synthase mcsA - *Aspergillus nidulans* | **-5** | **-3.3** | **-3.3** | 1.1 | 1.1 | 1.2 | GO:0006099 GO:0019679 |
| An01g11320 |  | similarity to dopa decarboxylase 3 DDC3 patent WO9960136-A1 - *Aspergillus oryzae* | **-5** | **-2.5** | **-2.5** | **2.0** | 1.1 | **2.2** | * |
| An05g00990 |  | strong similarity to malate dehydrogenase mdh - *Methanothermus fervidus* | -1.4 | -1.4 | **-1.7** | 1.0 | -1.1 | -1.1 | * |
| An11g00530 |  | strong similarity to ATP citrate lyase - *Homo sapiens* | -1.4 | **-1.7** | **-1.7** | -1.2 | 1.0 | -1.2 | * |
| **Carbon Metabolic/Catabolic Process** | | | | | | | | | |
| An08g03070 | *GCV1* | strong similarity to glycine decarboxylase subunit T Gcv1 - *Saccharomyces cerevisiae* | **-2.5** | **-2.5** | **-2.5** | -1.2 | 1.2 | 1.0 | GO:0006544 |
| An08g04390 | *FUN40* | strong similarity to glycine decarboxylase subunit H Fun40 - *Saccharomyces cerevisiae* | **-2.5** | **-2.5** | **-2.5** | -1.1 | 1.0 | -1.1 | GO:0006544 |
| An15g03260 |  | strong similarity to threonine aldolase - *Ashbya gossypii* | **-2** | **-1.7** | **-1.7** | 1.2 | 1.1 | 1.3 | GO:0006544  GO:0006567 |
| An14g01150 |  | strong similarity to glycine dehydrogenase component P - *Pisum sativum* | **-2.5** | **-3.3** | **-2.5** | -1.2 | 1.1 | -1.1 | GO:0006544 |
| An09g00260 | *aglC* | alpha-galactosidase aglC - *Aspergillus niger* | **-10** | **-10** | **-10** | -1.1 | 1.0 | -1.1 | GO:0006012 |
| An11g01120 |  | strong similarity to NADPH-dependent aldehyde reductase - *Sporobolomyces salmonicolor* | **-2** | **-2.5** | **-3.3** | -1.2 | -1.1 | -1.4 | GO:0006012  GO:0006071 |
| An02g03590 | *GAL7* | strong similarity to UDP-glucose-hexose-1-phosphate uridylyltransferase Gal7 - *Saccharomyces cerevisiae* | **-2.5** | **-2.5** | **-2.5** | -1.1 | 1.1 | 1.0 | GO:0006012 |
| An16g04160 | *GAL1* | strong similarity to galactokinase Gal1 - *Saccharomyces cerevisiae* | **-1.7** | **-1.4** | **-1.4** | 1.1 | 1.0 | 1.1 | GO:0006012  GO:0008645 |
| An11g10890 | *GAL10* | similarity to UDP-glucose 4-epimerase Gal10 - *Saccharomyces cerevisiae* | **-2** | **-2** | **-2** | 1.1 | 1.0 | 1.1 | GO:0006012  GO:0006979 |
| An02g11320 | *GAL10* | similar to UDP-glucose epimerase GAL10 | **-2.5** | **-3.3** | **-3.3** | 1.0 | 1.0 | -1.1 | GO:0006012  GO:0006979 |
| An04g06920 | *AgdA* | extracellular alpha-glucosidase - *Aspergillus niger* | **-5** | **-10** | **-10** | -2.5 | -1.1 | **-2.5** | * |
| An01g10930 | *AgdB* | Putative α–glucosidase - agdB - *Aspergillus niger* | **-10** | **-10** | **-10** | -1.4 | 1.0 | -1.4 | GO:0005982  GO:0005984 |
| An11g03340 | *AamA* | acid alpha-amylase - *Aspergillus niger* | **-370** | **-50** | **-50** | -1.4 | 1.0 | -1.4 | GO:0005982 |
| An09g03100 | *AmyA* | strong similarity to alpha-amylase precursor AMY - *Aspergillus shirousamii* | **-5** | **-5** | **-5** | -1.1 | 1.0 | -1.1 | * |
| An03g06550 | *GlaA* | glucan 1,4-alpha-glucosidase glaA - *Aspergillus niger* | **-10** | **-25** | **-25** | -2.5 | -1.1 | **-3.3** | * |
| An04g06910 | *AmyR* | transcription regulator of maltose utilization amyR - *Aspergillus niger* | **-3.3** | **-3.3** | **-3.3** | -1.1 | 1.0 | -1.1 | GO:0005982 |
| An01g01540 |  | strong similarity to alpha,alpha-trehalase treA - *Aspergillus nidulans* | **-3.3** | **-3.3** | **-3.3** | -1.2 | 1.0 | -1.1 | GO:0005984 |
| An11g10990 | *TPP* | strong similarity to trehalose-6-phosphate phosphatase TPP from patent WO200116357-A2 - *Saccharomyces cerevisiae* | **-2.5** | **-2.5** | **-3.3** | 1.0 | -1.1 | -1.1 | GO:0005984 |
| An01g10350 | *lacA* | strong similarity to secreted beta-galactosidase lacA - *Aspergillus niger* | **-2** | **-2.5** | **-2.5** | -1.1 | 1.0 | -1.1 | GO:0005984 |
| An11g03110 |  | strong similarity to methanol dehydrogenase Mdh - *Bacillus methanolicus* | **-2** | **-1.7** | **-1.7** | 1.1 | 1.0 | 1.1 | GO:0006071 |
| An04g04890 |  | strong similarity to glycerol kinase GK - *Mus musculus* | **-2.5** | **-1.7** | **-1.7** | 1.4 | 1.1 | 1.5 | * |
| An07g02210 | *PRPS1* | strong similarity to ribose-phosphate pyrophosphokinase Prps1 - *Saccharomyces cerevisiae* | **-2** | **-1.7** | **-2** | 1.1 | -1.1 | 1.0 | GO:0019321 |
| An15g05450 |  | strong similarity to NADPH-dependent carbonyl reductase S1 - *Candida magnoliae* | **-2.5** | **-2.5** | **-2.5** | -1.1 | 1.0 | 1.0 | GO:0019321 |
| An01g06970 | *ARA1* | strong similarity to D-arabinose dehydrogenase Ara1 - *Saccharomyces cerevisiae* | **-1.7** | **-2** | **-2** | -1.1 | 1.0 | 1.0 | GO:0019321 GO:0006071 |
| An04g05860 | *PRS2* | strong similarity to ribose-phosphate pyrophosphokinase Prs2 - *Saccharomyces cerevisiae* | **-1.7** | **-1.7** | **-1.7** | 1.0 | 1.0 | 1.0 | GO:0019321 |
| An11g02040 | *GND1* | strong similarity to phosphogluconate dehydrogenase Gnd1 - *Saccharomyces cerevisiae* | **-1.4** | **-1.7** | **-1.7** | -1.1 | -1.1 | -1.2 | GO:0019321 GO:0006970 GO:0006740 |
| An01g05360 |  | strong similarity to 42 kDa endochitinase Tham-ch - *Trichoderma hamatum* | **-2** | **-2** | **-2** | 1.0 | 1.1 | 1.1 | * |
| An16g03330 |  | weak similarity to the endo-1,4-betaxylanase gene product CAA93120.1 - *Ascochyta pisi* | **-2** | -1.2 | -1.2 | **1.7** | 1.1 | **1.9** | * |
| An01g03480 |  | strong similarity to sorbitol dehydrogenase gutB - *Bacillus subtilis* | **-2.5** | **-1.7** | **-1.7** | 1.7 | 1.0 | **1.8** | * |
| An02g11360 |  | strong similarity to the putative endo alpha-1,4 polygalactosaminidase precusor gene - *Pseudomonas sp* | **-3.3** | **-3.3** | **-3.3** | 1.0 | 1.1 | 1.1 | * |
| An12g07450 | *RGT2* | strong similarity to glucose sensor RGT2 - *Saccharomyces cerevisiae* | **-5** | **-10** | **-10** | -1.4 | 1.0 | -1.4 | * |
| An18g05160 |  | strong similarity to phosphoacetylglucosamine mutase AGM1 - *Candida albicans* | **-1.7** | **-1.7** | **-2** | 1.1 | -1.1 | 1.0 | * |
| An07g08720 | *TSL1* | strong similarity to 123K chain alpha,alpha-trehalose-phosphate synthase (UDP-forming) TSL1 - *Saccharomyces cerevisiae* | **-2.5** | **-3.3** | **-2.5** | -1.1 | 1.0 | -1.1 | * |
| An12g09130 |  | similarity to glucanase ZmGnsN3 of patent WO200073470-A2 - *Zea mays* | **-1.7** | **-1.4** | **-1.7** | 1.1 | 1.0 | 1.0 | * |
| An03g05940 | *GFA1* | strong similarity to glutamine--fructose-6-phosphate transaminase gfa1 - *Saccharomyces cerevisiae* | **-2.5** | -1.4 | -1.4 | 1.7 | 1.0 | **1.7** | * |
| An12g00100 |  | weak similarity to putative polysaccharide synthase homolog cap3B - *Streptococcus pneumoniae* | **-1.7** | **-1.4** | **-1.7** | 1.0 | 1.0 | 1.0 | * |
| An02g10310 |  | strong similarity to glycogen synthase - *Neurospora crassa* | **-2.5** | **-2.5** | **-2.5** | 1.0 | 1.0 | 1.0 | * |
| An08g06350 |  | strong similarity to phosphomannose isomerase manA - *Aspergillus nidulans* | **-1.4** | **-1.7** | **-1.7** | 0.9 | 1.0 | 0.9 | * |
| **Transporters** | | | | | | | | | |
| An02g03540 | *HXT3* | strong similarity to hexose transporter Hxt3 - *Saccharomyces cerevisiae* | **-2** | **-2** | **-2** | -1.1 | 1.1 | 1.0 | GO:0008645 |
| An11g01100 |  | strong similarity to high-affinity glucose transporter HGT1 - *Kluyveromyces lactis* | **-5** | **-5** | **-5** | -1.1 | 1.0 | -1.1 | GO:0008645 |
| An02g09540 | *HNM1* | strong similarity to choline permease Hnm1 - *Saccharomyces cerevisiae* | **-5** | **-3.3** | **-3.3** | 1.6 | 1.0 | 1.6 | GO:0015812 |
| An16g02000 |  | strong similarity to GABA permease gabA - *Aspergillus nidulans* | **-5** | **-3.3** | **-3.3** | 1.3 | -1.1 | 1.2 | GO:0015812 |
| An14g01850 | *UGA4* | strong similarity to GABA permease Uga4 - *Saccharomyces cerevisiae* | **-1.7** | **-2** | **-1.7** | -1.1 | 1.0 | -1.1 | GO:0015812 |
| An03g00430 |  | strong similarity to permease involved in fumonisin degradation from patent WO200105980-A1 - *Exophiala spinifera* | **-1.4** | **-1.4** | **-1.4** | 1.0 | 1.0 | 1.1 | GO:0015812 |
| **Response to oxidative stress** | | | | | | | | | |
| An18g01170 |  | strong similarity to poly(ADP-ribose) polymerase NAP protein from patent WO200004173-A1 - *Zea mays* | **-3.3** | **-3.3** | **-3.3** | -1.1 | 1.0 | -1.2 | GO:0006979 |
| An02g07930 |  | strong similarity to precursor of linoleate diol synthase - *Gaeumannomyces graminis* | **-3.3** | **-3.3** | **-3.3** | 1.0 | 1.0 | 1.0 | GO:0006979 |
| An07g03980 |  | strong similarity to osmotic sensitivity MAP Kinase OSM1 - *Pyricularia grisea* | **-1.7** | **-1.7** | **-2** | 1.0 | 1.0 | 1.0 | GO:0006979 |
| An06g01660 |  | strong similarity to thioredoxin peroxidase PMP20 - *Mus musculus* | **-2.5** | **-2.5** | **-2.5** | 1.0 | -1.1 | -1.1 | GO:0006979 |
| An04g04870 | *SOD2* | strong similarity to superoxide dismutase Sod2 - *Saccharomyces cerevisiae* | **-1.4** | **-1.7** | **-1.7** | -1.1 | -1.1 | -1.1 | GO:0006979 |
| An16g06100 |  | similarity to glutathione S-transferase GST1 - *Ascaris suum* | **-2.5** | **-2** | **-2** | 1.3 | 1.1 | 1.4 | GO:0006979 |
| An02g05830 |  | strong similarity to mannitol-1-phosphate 5-dehydrogenase mtlD - *Streptococcus mutans* | **-2.5** | **-2.5** | **-2** | 1.2 | 1.0 | 1.2 | GO:0006979 |
| An08g02310 |  | similarity to HC-toxin non-ribosomal peptide synthase HTS1 - *Cochliobolus carbonum* | **-2.5** | **-2.5** | **-2.5** | -1.1 | 1.1 | 1.0 | GO:0006979 |
| An16g00920 |  | strong similarity to peroxisomal membrane protein PMP20 - *Candida boidinii* | **-2.5** | **-2.5** | **-2.5** | -1.1 | 1.1 | 1.0 | GO:0006979 |
| An15g03220 | *GRX4* | strong similarity to member of the subfamily of yeast glutaredoxins Grx4 - *Saccharomyces cerevisiae* | **-1.4** | **-1.7** | **-1.7** | -1.1 | 1.0 | -1.1 | GO:0006979 |
|  |  |  |  |  |  |  |  |  |  |

GO:0006096: glycolysis; GO:0006094: gluconeogenesis; GO:0006083: acetate metabolic process; GO:0008645: hexose transport; GO:0006740: NADPH regeneration; GO:0019321: pentose metabolic process; GO:0006979: response to oxidative stress; GO:0006083: acetate metabolic process; GO:0019679: propionate metabolic process; GO:0046185: aldehyde catabolic process; GO:0006071: glycerol metabolic process; GO:0033609: oxalate metabolic process; GO:0006567: threonine catabolic process; GO:0006099: tricarboxylic acid cycle; GO:0006544: glycine metabolic process; GO:0006012: galactose metabolic process; GO:0005982: starch metabolic process; GO:0005984: disaccharide metabolic process; GO:0015812: gamma-aminobutyric acid transport. Values in bold represent a significant fold change with a FDR<0.005.
