# Supplementary material for: Genome-wide expression analysis upon constitutive activation of the HacA bZIP transcription factor in Aspergillus niger reveals a coordinated cellular response to counteract ER stress
Source: BMC Genomics. 2012 Jul 30;13:350. doi: 10.1186/1471-2164-13-350 (PMC3472299; doi:10.1186/1471-2164-13-350)
Supplement: Additional file 16 — Commonly induced and repressed genes in the HacACA strain and A. niger strains treated with DTT and Tunicamycin and expressing tPA. Subset of all differentially expressed genes (Additional file 3) and Guillemette’ study [37]. [file 1471-2164-13-350-S16.doc]

Additional file 16: Commonly induced and repressed genes in the HacA^CA^ strain (this study) and *A. niger* strains treated with DTT and Tunicamycin and expressing tPA [37].

| **Category** | **Gene ID** |
| --- | --- |
| **Induced** | |
| Protein folding | An01g13220*, An02g14800*, An01g04600*, An01g08420*, An11g04180*, An16g07620*, An18g02020, An04g02020, An11g11250, An05g00880 |
| Translocation/signal peptidase complex | An03g04340, An01g13070, An16g08830, An17g00090, An01g03820, An01g11630, An01g10070, An04g06890, An16g07390, An09g05420*, An01g00560, An15g06470 |
| Glycosylation | An14g05910, An18g02360, An03g04410*, An02g03240, An07g06430, An07g04190, An02g14560, An18g03920, An02g14930, An16g08570, An18g04260*, An18g06220, An12g00340, An09g05880, An13g00620, An15g01420, An16g04330, An04g05250, An01g05200, An02g14940 |
| Vesicle trafficking/transport | An03g04940*, An01g04320, An04g08830, An02g02640, An14g00210, An02g08450, An02g05870, An07g07340, An09g04170, An02g11990, An08g00290, An08g06780 |
| Proteolytic degradation | An16g06750, An08g09000, An01g12720 |
| Lipid/inositol metabolism | An02g13410*, An11g02990, An08g00560, An02g07610, An16g01820, An07g09840, An16g09180, An12g03150, An19g00320 |
| Transcription | An07g03760 |
| Translation | An14g06610, An04g08580 |
| Stress related | An12g03580, An01g14100 |
| Cell cycle an DNA processing | An01g08170 |
| Other | An11g04750, An14g07030 |
| Unclassified | An08g03960*, An08g03970, An09g00650*, An14g02470, An16g08470, An08g08090, An04g02250, An08g00900, An14g01990, An08g04260, An07g10280, An09g06130, An08g03970, An15g01680, An18g06120, An14g06550, An18g06740, An11g10800, An18g01690, An02g02260 |
| **Repressed** | |
| Protein fate | An06g01610, An13g02540, An02g00960, An04g1440 |
| Vesicle trafficking/transport | An09g02930 |
| Lipid metabolism | An16g01880, An03g03550, An02g09540, An16g06090 |
| Amino acid metabolism | An15g06700, An01g14730, An07g04300 |
| Phosphate metabolism | An12g01910 |
| C-compound and carbohydrate metabolism | An19g00100, An16g06800, An02g11320, An09g00260, An01g01540 |
| Metabolism of vitamins, cofactors and prosthetic groups | An01g04250 |
| Cellular transport | An01g01620, An12g10320, An07g06240, An08g04670, An16g06740, An03g00680 |
| Cell rescue, defense and virulence | An18g00980, An16g05920 |
| Nucleotide/nitrogen and sulfur metabolism | An10g00800, An18g01220 |
| Other | An16g05910, An15g06140 |
| Unclassified | An02g06440, An14g01820, An02g08050, An16g03330, An08g02300, An02g08330, An03g03530, An03g00840, An08g07150, An11g01660, An18g01000, An12g02660, An13g01520, An16g05930, An02g00120 |

* genes induced in all the conditions (HacA^CA^, DTT, Tunicamycin and tPA)
