# Supplementary material for: Genome-wide expression analysis upon constitutive activation of the HacA bZIP transcription factor in Aspergillus niger reveals a coordinated cellular response to counteract ER stress
Source: BMC Genomics. 2012 Jul 30;13:350. doi: 10.1186/1471-2164-13-350 (PMC3472299; doi:10.1186/1471-2164-13-350)
Supplement: Additional file 17 — Expression values of genes related to extracellular proteases production. [file 1471-2164-13-350-S17.doc]

Additional File 17. Expression values of genes related to extracellular proteases production.

| **Gene ID** | **Gene name: *A. niger*** | **Description** | **Fold change** | | | | | |
| --- | --- | --- | --- | --- | --- | --- | --- | --- |
|  |  |  | **HacA^CA-1^/**  **HacA^WT^** | **HacA^CA-2^/**  **HacA^WT^** | **HacA^CA-3^/**  **HacA^WT^** | **HacA^CA-2^/**  **HacA^CA-1^** | **HacA^CA-2^/**  **HacA^CA-3^** | **HacA^CA-3^/**  **HacA^CA-1^** |
| An14g04710 | *pepA* | aspartic proteinase aspergillopepsin I pepA – *A. niger* | -1.1 | -1.1 | -1.1 | 1.0 | 1.0 | 1.1 |
| An01g00530 | *pepB* | proteinase aspergillopepsin II – *A.niger* | 1.0 | 1.0 | 1.0 | 1.0 | -1.1 | 1.1 |
| An09g03780 | *pepD* | subtilisin-like serine protease pepD – *A. niger* | 1.1 | 1.1 | 1.0 | -1.1 | 1.0 | -1.1 |
| An07g0830 | *pepF* | cyclophilin-like peptidyl prolyl cis-trans isomerase cypA *– A. niger* | -1.1 | **-1.2** | **-1.2** | 1.0 | 1.0 | 1.0 |
| An04g06940 | *prtT* | transcriptional activator of proteases PrtT – *A. niger* | **-1.4** | **-1.2** | **-1.2** | 1.1 | 1.0 | 1.2 |

Values in bold represent a significant fold change with a FDR<0.005.
